# Deciphering complex breakage-fusion-bridge genome rearrangements with Ambigram

**DOI:** 10.1038/s41467-023-41259-w

**Published:** 2023-09-08

**Authors:** Chaohui Li, Lingxi Chen, Guangze Pan, Wenqian Zhang, Shuai Cheng Li

**Affiliations:** Department of Computer Science, City University of Hong Kong, Hong Kong, China

**Keywords:** Software, Genome assembly algorithms

## Abstract

Breakage-fusion-bridge (BFB) is a complex rearrangement that leads to tumor malignancy. Existing models for detecting BFBs rely on the ideal BFB hypothesis, ruling out the possibility of BFBs entangled with other structural variations, that is, *complex BFBs*. We propose an algorithm Ambigram to identify complex BFB and reconstruct the rearranged structure of the local genome during the cancer subclone evolution process. Ambigram handles data from short, linked, long, and single-cell sequences, and optical mapping technologies. Ambigram successfully deciphers the gold- or silver-standard complex BFBs against the state-of-the-art in multiple cancers. Ambigram dissects the intratumor heterogeneity of complex BFB events with single-cell reads from melanoma and gastric cancer. Furthermore, applying Ambigram to liver and cervical cancer data suggests that the BFB mechanism may mediate oncovirus integrations. BFB also exists in noncancer genomics. Investigating the complete human genome reference with Ambigram suggests that the BFB mechanism may be involved in two genome reorganizations of *Homo Sapiens* during evolution. Moreover, Ambigram discovers the signals of recurrent foldback inversions and complex BFBs in whole genome data from the 1000 genome project, and congenital heart diseases, respectively.

## Abstract (web summary)

Breakage-fusion-bridge (BFB) is a mechanism that leads to complex genome rearrangements in multiple cancers. Here, the authors develop a computational method for identifying these events, even when further complicated by additional structural variations.

## Introduction

**Fig. 1:**
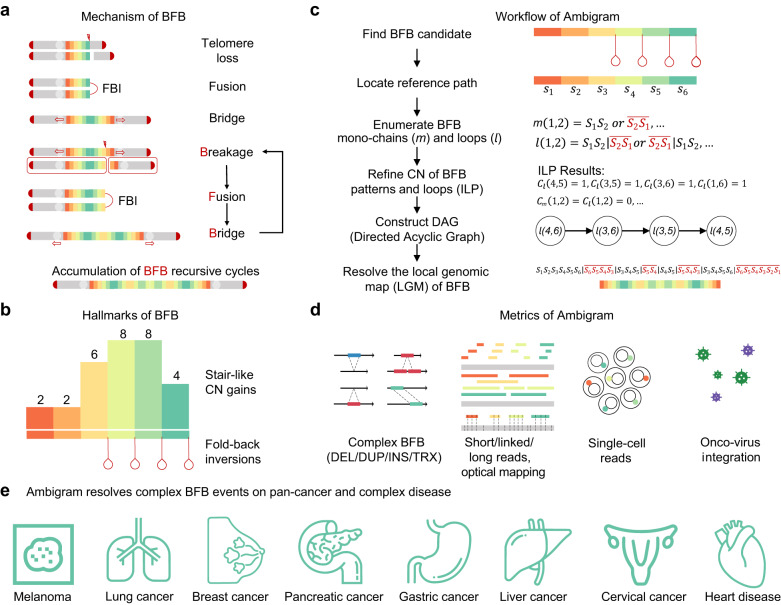
Schematic overview. **a** The mechanism of BFB. **b** The hallmarks of BFB. **c** Workflow of Ambigram. **d** Metrics of Ambigram. **e** Summary of Ambigram applications. All anatomy images with free licenses are provided by Freepik. BFB breakage-fusion bridge, FBI fold-back inversion, CN copy number, ILP integer linear programming, DEL deletion, DUP duplication, INS insertion, TRX translocation.

Breakage-fusion-bridge (BFB) is a mechanism that leads to complex genome rearrangements in multiple cancers^1–13^. The rearrangement of BFB is mediated by the recursive cycles of BFB^14–16^ (Fig. 1a). A BFB cycle begins with the fold-back inversion (FBI) of two sister chromatids due to the lack of telomeres during DNA replication, resulting in two-centromeres in the fused bridge. When two centromeres are stretched to opposite poles in the anaphase, the two sister chromatids are split with a double-strand break on the bridge between two centromeres, unnecessarily the same as the previous fusion site. Since each daughter cell contains chromatids without telomeres, another BFB cycle may start again. Repetition of BFB cycles contributes to a surge in stair-like copy number (CN) amplifications and FBIs^15–18^. The above depicts a *perfect BFB* event that is solely driven by *reverse complementary FBI*, where the genomic positions of two breakpoints of a reverse complementary FBI are the same. However, some BFB events involve *imperfect FBI* whose breakpoint positions are different, resulting in the loss of DNA segments near the breakpoints^4^. Furthermore, studies reported that structure variations (SVs) such as deletion, duplication, insertion, and translocation could be involved during BFB cycles outside the FBI breakpoints^13,19–21^. In this study, we coin the BFB rearrangement beyond perfect BFB as *complex BFB* rearrangement.

As the BFB process delivers anaphase bridges and dicentric chromosomes, investigators detected it using classical cytogenetic techniques in the early time^10^. However, these BFB cytogenetic signatures are not directly discernible from high-throughput DNA sequencing reads. Most studies inferred the consistency of a specific observation with BFB events from sequencing reads by two distinct hallmarks^3–9,11,12,16^: (i) oscillating CN with exponential or stair-like gains; and ii) enrichment of FBIs with the fold-back direction of head-to-head (FBI-hh) or tail-to-tail (FBI-tt) (Fig. 1b). However, a pattern consistent with the two hallmarks does not imply that BFB yields the pattern.

Leveraging the “palindrome” or “ambigram” nature of the BFB-induced *local genomic map*, i.e., the rearranged structure of the local genome, researchers started to utilize well-established algorithms to mathematically expand CNs or FBIs into possible BFB paths for array comparative genomic hybridization (aCGH) or pair-end sequencing (PE) data (Kinsella et al.^22^, BFBFinder^23,24^, and Greenman et al.^25,26^, Table 1). However, these mathematical models have limitations in interpreting real-world data for the following reasons. (i) These models solely focus on perfect BFB, ruling out the possibility of other deletion, duplication, insertion, or translocation in complex BFB rearrangements^13,19–21^. (ii) These models lag behind recent advances in linked read sequencing^27,28^, long-read sequencing^29,30^, and optical mapping alignment^31^. Linked read, long read, and optical mapping data offer larger than 100 kb linkage of the DNA fragment that could accelerate the detection of the accurate and long complex BFB local genomic map. (iii) Single-cell sequencing^32–35^ could facilitate investigation of the intratumor heterogeneity of complex BFB at single-cell resolution while existing methods are incompatible. Recently, the community has developed computational pipelines to detect and resolve complex somatic genome rearrangements, including complex BFB (AmpliconArchitect + AmpliconClassifier^19^, AmpliconReconstructor^20^, and LINX^21^, Table 1). However, these pipelines merely support short sequencing reads or optical mapping data.

**Table 1:** Summary of BFB detection tools

Tool	LGM-resolved	Feature					Experiment data protocol						
		CN	BP	FBI	DEL/DUP INS/TRX	Virus	aCGH	PE	10x	PB	ONT	OM	SC
Kinsella et al.^22^	*✓*	*✓*	–	–	–	–	*✓*	–	–	–	–	–	–
BFBFinder^23,24^	*✓*	*✓*	–	+	–	–	*✓*	*✓*	–	–	–	–	–
Greenman et al.^25,26^	*✓*	*✓*	*✓*	–	–	–	–	*✓*	–	–	–	–	–
AA + AC^19^	–	*✓*	*✓*	*✓*	*✓*	+	–	*✓*	–	–	–	–	–
AR^20^	*✓*	*✓*	*✓*	*✓*	*✓*	+	–	–	–	–	–	*✓*	–
LINX^21^	*✓*	*✓*	*✓*	*✓*	*✓*	+	–	*✓*	–	–	–	–	–
Ambigram	*✓*	*✓*	*✓*	*✓*	*✓*	*✓*	–	*✓*	*✓*	*✓*	*✓*	*✓*	*✓*

"*✓*” for available and “–” for not applicable. The FBI feature of Zakov et al. is marked by “+” since Zakov et al. solves the BFB using the CN count vector first, then uses the FBI fraction to verify the confidence of the BFB. Greenman et al. solely use FBI breakpoints, neglecting the head-to-head or tail-to-tail direction of the FBI. The virus feature of the complex rearrangement detection pipelines AA + AC, AR, and LINX are indicated by “+” because they resolve BFB and virus integration individually rather than correlating BFB and viral integration into one evolution process. *BFB* breakage-fusion-bridge, *LGM* local genomic map, *CN* copy number, *BP* breakpoint, *FBI* fold-back inversion, *DEL* deletion, *DUP* duplication, *INS* insertion, *TRX* translocation, *aCGH* array comparative genomic hybridization, *PE* paired-end, *10x* 10x linked-reads, *PB* PacBio, *ONT* Oxford Nanopore, *OM* optical mapping, *SC* single cell, *AA* AmpliconArchitect, *AC *AmpliconClassifier, *AR* AmpliconReconstructor.

In this work, to overcome the limitations above, we propose Ambigram, a graph algorithm to detect the complex BFB and reconstruct the underlying local genomic map during the evolution process. Ambigram deciphers the complex BFB event that involves deletion, duplication, insertion, and translocation. Ambigram resolves gold- or silver-standard complex BFB events from various data protocols, encompassing short, linked, long, or optical mapping data from multiple cancers, including melanoma, lung, breast, and pancreatic cancer. Incorporating linkages from linked read, long read, or optical mapping data boosts the efficacy of BFB reconstruction in melanoma and lung cancer. Ambigram is robust with various sequencing depths and tumor purities as well. Furthermore, applying the tool to single-cell reads suggests that melanoma and gastric cancers may have intratumor heterogeneity of complex BFBs. Ambigram demonstrates that the BFB mechanism can mediate oncovirus integrations, including seven HBV integrations in four liver cancer samples and three HPV integrations in three cervical cancer samples, leading to truncation of the tumor suppressor *FHIT* or amplification of oncogenes *MUC12*, *BORA*, *DIS3*, *PIBF*, or *ZNFs*. Although BFB is proposed as a mechanism that leads to complex genome rearrangements in cancers. We find BFB signals from non-cancer data. We suggest that two genome reorganizations of *Homo Sapiens* during evolution may be carried out by the BFB mechanism after investigating the complete human genome reference. Ambigram discovers 85 recurrent FBIs or complex BFB polymorphisms (occurring in more than 5% of the samples in the cohort) in 923 whole genomes from the 1000 Genome Project (1000GP), directly truncating 32 genes, including *APP*, *C4BPA*, and *SUGCT*. We apply Ambigram to 330 congenital heart disease (CHD) probands and 612 of their parents, with 923 1000GP samples as unaffected controls. We observe the recurrent FBIs or complex BFB polymorphisms across the cohorts. The three genes that are more frequently (>50%) involving FBIs are *APP*, *C4BPA*, and *BORCS5*. We find that *PTPRQ*, *PUS7*, *ITPRID1*, *PLEKHB2*, *IL1RAPL1,* and *EXT1* that carry FBI or complex BFB polymorphisms are significantly (*p* value <1e-5) related to CHD.

## Results

### Overview of Ambigram

Given the collection of SVs derived from genome sequencing data, we first identify the set of BFB candidate SVs that contain FBIs (Fig. 1a–c). Then, we locate a local genome region that includes all the SV breakpoints of a candidate BFB event. This region of the genome forms the *reference path* of that candidate BFB event, and we partition it into continuous segments by breakpoints. Based on the mathematical model that we designed to represent BFB, Ambigram finds all possible BFB *mono-chains* and *loops* (Methods). Then, Ambigram employs integer linear programming (ILP) to estimate the CN configuration of BFB mono-chains and loops that best match the BFB CN pattern. Next, we utilize the CN configuration to construct a *BFB directed acyclic graph* (DAG). The DAG has the mono-chains and loops as vertices. We connect the vertices *u* and *v* by a directed edge if *v* is a child entity of *u* (Methods). Finally, the *BFB path*, i.e., the local genomic map of BFB is constructed by a walk guided by the DAG. Ambigram owns several merits (Fig. 1d). Ambigram deals with complex BFB events that involve other types of SV, such as deletion, duplication, insertion, and translocation, which are common in practice. Furthermore, Ambigram is compatible with multiple data protocols, including Illumina pair-end (PE) short reads, 10x Genomics linked reads, Pacific Biosciences (PB) long reads, Oxford Nanopore (ONT) long reads, Bionano optical mapping (OM) alignment, and single-cell reads. Additionally, Ambigram can also resolve potential BFB paths mediated by oncovirus integration. This study demonstrates that Amibgram resolves complex BFB events in multiple cancers and complex diseases (Fig. 1e).

### Benchmarking Ambigram with in silico data

**Fig. 2:**
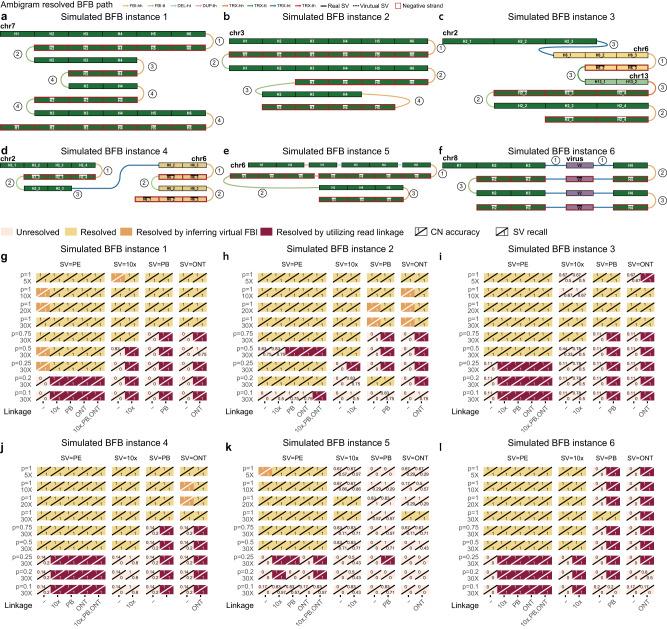
In silico experiments. **a**–**f** The BFB paths of simulated instances 1–6. Detailed captions are in Supplementary Figs. 1–6. The number *n* in a circle denotes that the SV comes from chromosome duplication in the *n*-th BFB cycle. **g**–**l** Results derived by Ambigram for simulated BFB instances 1–6 with various sequencing protocols, depths, and purities. “-” means that the inputs of Ambigram are SVs called from one sequencing protocol (SV=PE, SV=10x, SV=PB, or SV=ONT) and ground truth CNs. “Resolved” means all SVs and CNs from the inferred BFB path are matched with those of ground truths, otherwise “Unresolved''. “Resolved by inferring the virtual FBI” signifies Ambigram resolves the BFB path by recovering the undetected FBIs in low sequencing depth and tumor purity scenarios. “Resolved by utilizing read linkage” means that Ambigram cannot resolve the BFB path with CNs and detected SVs, while it can resolve the path after incorporating the linked or long-read linkage from 10x, PB, or ONT data. The CN accuracy is measured by the number of segments with correctly inferred CNs divided by the total segment number. The SV recall measures the portion of the SVs inferred correctly, that is, the number of ground truth SVs inferred correctly by the tool over the total number of ground truth SVs; Note that “Resolved” implies CN accuracy = 1, SV precision = 1, SV recall = 1, and SV F1-score= 1. BFB breakage-fusion-bridge, FBI-hh fold-back inversion with head-to-head direction, FBI-tt fold-back inversion with tail-to-tail direction, DEL deletion, DUP duplication, INS insertion. TRX translocation. SV structure variation. PE paired-end. 10x 10x linked-reads. PB PacBio. ONT Oxford Nanopore.

We designed six in silico BFB instances covering one perfect BFB and five complex BFBs. The first instance simulates a perfect BFB event on chr7 with four BFB cycles that reverse complementary FBI fused DNA segments $H6-\overline{H6},H2-\overline{H2},H4-\overline{H4}$, and $H3-\overline{H3}$ orderly (Fig. 2a). The second instance simulates a complex BFB that involves imperfect FBIs with loss of DNA segments at breakpoints; it is formed by four BFB cycles with two reverse complementary FBIs and two imperfect FBIs on chr3 (Fig. 2b). The third and fourth instances simulate BFB events involving insertion and translocation (Fig. 2c–d). Then, we designed a complex BFB event that covered duplication and insertion outside the FBI breakpoints as the fifth instance (Fig. 2e). The sixth instance event simulates the virus integration scenario (Fig. 2f). The detailed descriptions of these BFB events are in Supplementary Fig. 1–6.

We simulated the local genome sequencing reads according to the six BFB paths to verify the compatibility of different protocols (PE, 10x, PB, and ONT). The tumor purity is profoundly shifting in clinical samples. Thus, we evaluated the impact of tumor purity on BFB path reconstruction. We randomly mixed BFB path reads and reference path reads with a depth of 30× and tumor purities of 100%, 75%, 50%, 25%, 20%, and 10% for the PE, 10x, PB, and ONT data, respectively. Sequencing depth is also a significant factor, as it is a trade-off between the sensitivity of BFB detection and the cost of sequencing. To evaluate the effect of sequencing depth on deciphering the BFB path, we sampled 100% pure tumor reads at depths of 30×, 20×, 10×, and 5×.

We ran Ambigram to reconstruct the six in silico BFB instances using the ground truth CN profiles of segments, and the SVs called from PE, 10x, PB, and ONT reads independently. We regard the BFB event as fully resolved if all CNs and SVs from the inferred BFB path are matched with those of ground truths; that is CN accuracy = 1, SV precision=1, SV recall = 1, and SV F1-score = 1, otherwise, unresolved. Ambigram successfully resolved the BFB paths with purity 100% and sequencing depth ranging from 5× to 30× for all protocol data of six events (Fig. 2g–l and Supplementary Fig. 7); exception happens on the third instance (10× data) and the fifth instance (10×, PB, and ONT data). Due to the complexity of the designed instances and sequencing noise from low coverage, the SV callers (Methods) were unable to call enough ground truth SVs to allow Ambigram to infer the exact BFB path (Supplementary Fig. 8). Furthermore, Ambigram loses sensitivity while tumor purity decreases when sequencing depth is set to 30× because ground truth SV supporting reads are reduced in low purity, especially in BFB scenario 5 (Fig. 2k and Supplementary Fig. 8). Notably, Ambigram can infer virtual FBIs and SVs that are undetected due to low depth and purity to form BFB paths, instances resolved by inferring virtual FBI are colored with orange in Fig. 2g–l. Furthermore, in addition to SV and CN information, Ambigram can incorporate read linkage evidence from single- or multiple-sequencing protocols to infer the BFB paths. Figure 2g–l shows that by adding linked or long-read linkage information (colored with crimson), five BFB instances are resolved in purity 0.1 scenarios.

BFBFinder, AmpliconArchitect + AmpliconClassifier, AmpliconReconstructor, and LINX are cutting-edge BFB detection tools with executables (Table 2). However, AmpliconArchitect + AmpliconClassifier and LINX failed the trials of 100% purity and 30× PE BAM files following the GitHub guides. AmpliconReconstructor requires the breakpoint graph from AmpliconArchitect as input, which makes it inapplicable. Therefore, we can only benchmark Ambigram with BFBFinder. We fit the ground truth CN profiles of the BFB paths of each chromosome independently since BFBFinder only takes segment CN profiles from single chromosomes (Method). In Supplementary Fig. 9, BFBFinder successfully resolves the perfect BFB event (the first instance). Even though BFBFinder resolves the BFB path of the third and fourth instances at the single chromosome level, it cannot assemble the BFB path after translocation. As for the second and fifth instances, the inferred BFB paths from BFBFinder are inconsistent with ground truths, as BFBFinder neglects the possibility of imperfect FBI, duplication, and deletion. The sixth simulated instance is inapplicable for BFBFinder as it involves virus integration.

**Table 2:** Summary of gold-standard datasets to benchmark Ambigram with the state-of-the-art

Dataset	Standard	Cancer type	Protocol	Data format	AA + AC	AR	LINX	Ambigram
In silico instances 1–6	Gold	–	PE	LGS	x	–	x	*✓*
In silico instances 1–6	Gold	–	10x/PB/ONT	LGS	–	–	–	*✓*
In silico instances 1–6	Gold	–	PE+10x/PB/ONT	LGS	–	–	–	*✓*
COLO829 instance 1	Gold	Melanoma	PE	WGS	+	–	*✓*	*✓*
COLO829 instance 2	Gold	Melanoma	PE	WGS	x	–	+	*✓*
COLO829 instances 1–2	Gold	Melanoma	10x/PB/ONT	WGS	–	–	–	*✓*
COLO829 instances 1–2	Gold	Melanoma	PE+10x/PB/ONT	WGS	–	–	–	*✓*
COLO829 instances 1–2	Gold	Melanoma	PE+OM	WGS	–	*✓*	–	*✓*
Input	In silico instances						COLO829 instances	
Ground Truth CN	1	2	3	4	5	6	1	2
BFBFinder	*✓*	x	+	+	x	–	+	+

"*✓*” means the BFB events are detected and resolved. “x” means the BFB events are unresolved after running the program. “–” means not applicable. The COLO829 instance 1 on AA + AC and COLO829 instance 2 on LINX are marked with “+” as the tool only detects the event but does not provide the BFB path. Even though BFBFinder resolves the BFB path of BFB instances 3–4 and COLO829 instances 1–2 at single chromosome, it cannot assemble the BFB path after translocation, so we marked these instances as “+”. *PE* paired-end, *10x* 10x linked-reads, *PB* PacBio, *ONT* Oxford Nanopore, *OM*optical mapping, *LGS* local genome sequencing. *WGS* whole genome sequencing. *AA* AmpliconArchitect. *AC* AmpliconClassifier. *AR* AmpliconReconstructor.

Last, we simulated 410 BFB paths without generating the sequencing reads. The BFB paths own varying numbers of segments (ranging from 6 to 15), FBIs (ranging from 2 to 7), total segment CN (ranging from 13 to 105), and largest segment CN (ranging from 3 to 11). After fitting the CN profile and SV profile of BFB paths, Ambigram demonstrates higher CN accuracy, SV Precision, SV Recall, and SV F1-score compared to BFBFinder (Supplementary Fig. 10).

### Evaluation of short, linked, or long-read data on cancers

Valle-Inclan et al. have manually verified two groups of ground truth somatic SVs that complex BFBs may cause in the COLO829 melanoma cell line^13,21^. The underlying complex BFBs are not only supported by FBIs and oscillation of CN but are also involved with deletion, insertion, and translocation. Here, we assessed Ambigram against the state-of-the-art in deciphering the two gold-standard complex BFB events using PE reads with 100% purity and 98× depth.

**Fig. 3:**
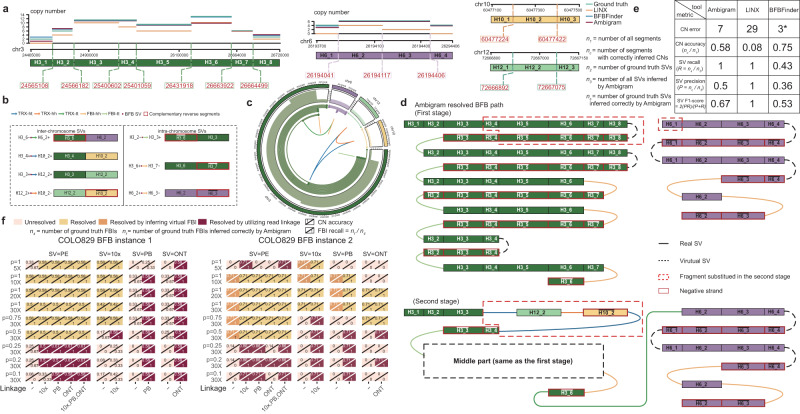
COLO829 instance 1 - complex BFB on chr3, chr6, chr10, and chr12. **a** The SV breakpoints split the local genome regions of chr3, chr6, chr10, and chr20 into 8, 4, 3, and 3 segments, respectively. **b** List of SVs and the segments they connect. **c** CIRCOS diagram of the complex BFB. **d** Ambigram resolved the BFB path with 98x depth and 100% purity PE sequencing data. In the first stage, six BFB cycles happen on chr3 with details shown in Supplementary Fig. 11. At the same time, chr6 undergoes four BFB cycles. In the second stage, an inter-chromosomal rearrangement inserts fragments of chr10 and chr12 into the BFB path on chr3. Another translocation connects two BFB paths on chr3 and chr6, constructing the result of the complex BFB event. **e** The total CN error, CN accuracy, SV recall, SV precision, and SV F1-score derived by Ambigram, LINX, and BFBFinder compared to ground truth. The total CN error is the sum of all segment copy number differences between the output and ground truth. BFBFinder is marked with “*” as BFBFinder merely accepts segment CN profiles from a single chromosome, so we fit the ground truth CN profiles of BFB paths of chr3 and chr6 separately. **f** Results derived by Ambigram with various sequencing protocols, depths, and purities. “-” means that the inputs of Ambigram are SVs called from one sequencing protocol (SV=PE, SV=10x, SV=PB, or SV=ONT) and ground truth CNs. “Resolved” means that inferred BFB path includes all ground truth FBIs, otherwise “Unresolved''. “Resolved by inferring the virtual FBI” signifies Ambigram resolves the BFB path by recovering the undetected FBIs in low sequencing depth and tumor purity scenarios. “Resolved by utilizing read linkage” means that Ambigram cannot resolve the BFB path with CNs and detected SVs, while it can resolve the path after incorporating the linked or long-read linkage from 10x, PB, or ONT data. BFB breakage-fusion-bridge. FBI-hh fold-back inversion with head-to-head direction. FBI-tt fold-back inversion with tail-to-tail direction. DEL deletion, DUP duplication, INS insertion, TRX translocation, SV structure variation, PE paired-end, 10x 10x linked-reads, PB PacBio, ONT Oxford Nanopore.

The first complex BFB event has three FBIs and four translocation SVs, integrating segments from chr3, chr6, chr10, and chr12 (Fig. 3a–c). Ambigram inferred that this complex BFB event had undergone two stages (Fig. 3d). In summary, chr3 and chr6 encounter six and four BFB cycles in the first stage separately. We depicted the detailed evolution process of the chr3 BFB cycles in Supplementary Fig. 11. In the second stage, segments of chr10 and chr12 are inserted into the BFB path on chr3 through inter-chromosomal rearrangements, and another translocation links the BFB paths of chr3 and chr6 together, contributing to the final BFB path. LINX suggested the whole BFB event consists of four BFB cycles, followed by a genome doubling (Supplementary Fig. 12)^13,21^. Ambigram resolved the BFB path superior to LINX in terms of CN, as Ambigram yields a copy number difference of seven from ground truth, while LINX produced 29; Ambigram has CN accuracy of 0.58 while LINX has CN accuracy of 0.08 (Fig. 3e). As AmpliconArchitect + AmpliconClassifier neglect the translocation between chr3 and chr6, it interprets the event with two independent CN amplicons (Supplementary Fig. 13a). Amplicon 1 covers complex CN gains in the local regions of chr3, chr10, and chr12. Amplicon 2 represents the CN gains on the local region of chr6. Even though amplicon 1 is detected as the BFB event, the BFB path is unresolved. BFBFinder merely accepts segment CN profiles from a single chromosome, so we fit the ground truth CN profiles of BFB paths of chr3 and chr6, separately (Supplementary Fig. 14a). Although BFBFinder had a smaller CN difference to ground truth and larger CN accuracy, it failed to assemble the complex BFB event with translocation and produced the lowest SV precision, SV recall, and SV F1-score compared to Ambigram and LINX (Fig. 3e).

The second complex BFB event occurs in chr15 and consists of one FBI and four translocations, leading to templated insertion from chr6 and chr20 (Supplementary Fig. 15a–c). Ambigram successfully resolved it with two stages, chr15 undergoes four BFB cycles in the first stage, and then translocation occurs in chr6, chr15, and chr20 (Supplementary Fig. 15d). LINX detected the event but provided no resolved BFB path. AmpliconArchitect + AmpliconClassifier detected the complex CN gains in the local regions of chr6, chr13, chr15, and chr20. However, it was not classified as a BFB event (Supplementary Fig. 13b). BFBFinder, like the first instance, obtained a lower CN error from single chromosomes but was unable to assemble the final BFB paths with the translocations (Supplementary Fig. 14b and Supplementary Fig. 15e).

We additionally evaluated Ambigram using a range of sequencing depths and purities (Table 2). We gathered COLO829 and COLO829BL PE short reads, 10x linked reads, PB long reads, and ONT long reads. We arbitrarily mixed COLO829 and COLO829BL reads with tumor purities of 100%, 75%, 50%, 25%, 20%, and 10%. Further, we sampled 100% pure tumor reads at depths of 30×, 20×, 10×, and 5×. We ran Ambigram to reconstruct the two complex BFB instances using the segment CN profiles curated by Valle-Inclan et al., and the SVs called from PE, 10×, PB, and ONT reads independently. We consider the BFB event as resolved in Fig. 3f and Supplementary Fig. 16 if the inferred BFB path includes all ground truth FBIs (FBI recall = 1), otherwise unresolved; the scenarios resolved by inferring virtual FBI and utilizing read linkage are colored with orange and crimson, respectively. Ambigram successfully resolved complex BFB path with purity 100% and sequencing depth higher than 20× from all data protocols for both events (Fig. 3f). Ambigram becomes less sensitive as depth or purity decreases, as the supporting reads of ground truth SVs are diminished in low depth or purity (Fig. 3f and Supplementary Fig. 17a–c). Ambigram inferred virtual FBIs that were undetected due to low depth and purity to form BFB paths in the second BFB instance (Fig. 3f and Supplementary Fig. 17a). Furthermore, by adding linked or long-read linkage information, Both BFB instances are resolved in depth 5× or purity 0.1 scenarios (Fig. 3f).

We further recruited three datasets, lung cancer cell line HCC827, breast cancer sample PD4875, and pancreatic cancer sample PD3641. The local genomic map of the BFB event in HCC827, PD4875, and PD3641 has been separately inferred by AmpliconReconstructor^20^, Greenman et al.^25^, and BFBFinder^23^. We consider the three BFB events inferred by state-of-the-art tools as silver-standard and check if Ambigram can resolve them (Table 3). With configuration on SVs and CNs, we successfully resolved the local BFB paths on all samples. The existence of a BFB event with four FBIs in HCC827 has been detected by AmpliconArchitect + AmpliconClassifier with PE short read data^19^ (Supplementary Fig. 18a–c). AmpliconReconstructor further inferred the underlying BFB path is derived from four BFB cycles by incorporating linkage information from optical mapping (OM) data^19^. Ambigram resolved the extract BFB path as AmpliconReconstructor with PE or PE plus OM data (Supplementary Fig. 18d). Moreover, when CN configuration and only one FBI are fitted into Ambigram, it successfully inferred the three virtual FBIs and resolved the BFB path (Supplementary Fig. 18e–f). For PD4875, Ambigram efficiently reconstructed the complex BFB path with translocation based on the information from SV and CN (Supplementary Fig. 19a), the same as Greenman et al. For PD3641, Ambigram resolved a BFB path that underwent five BFB cycles while BFBFinder interpreted it with seven BFB cycles (Supplementary Fig. 19b). We argue that according to the theory of Occam’s razor^36^, Ambigram is better than BFBFinder as it uses less information to interpret this BFB event.

**Table 3:** Summary of silver-standard datasets to benchmark Ambigram with the state-of-the-art

Dataset	Standard	Cancer type	Protocol	Data format	Originally inferred by	Ambigram
HCC827	Silver	Lung	PE	SV+CN	AA + AC	*✓*
HCC827	Silver	Lung	PE+OM	SV+CN	AA + AC + AR	*✓*
PD4875	Silver	Breast	PE	SV+CN	Greenman et al.	*✓*
PD3641	Silver	Pancreatic	PE	SV+CN	BFBFinder	*✓*

"*✓*” means the BFB events are detected and resolved. *BFB* breakage-fusion-bridge, *PE* paired-end, *OM* optical mapping, *SV+CN* called structure variation and copy number, *AA* AmpliconArchitect, *AC* AmpliconClassifier, *AR* AmpliconReconstructor.

### Intratumor heterogeneity of complex BFBs in single-cell data

Velazquez et al. performed single-cell DNA sequencing of 1,475 COLO829 cells. Although the CN profiles of single cells in COLO829 remain largely homogeneous (Supplementary Fig. 20a), Velazquez et al. demonstrated the heterogeneity of SVs in COLO829, leading to the evolution of its subclones^33^. Here, we check whether the two complex BFB instances discussed before exhibit heterogeneity in the subclone resolution. With hierarchical clustering, we labeled single cells with six major subclones (A-F) (Supplementary Fig. 20a). For the first complex BFB instance, no subclone agrees with bulk sequencing data due to the loss of FBIs and translocation. For the second complex BFB instance, there is an agreement of the local genomic map of BFB detected by subclones A, D, E, and F with the bulk sequencing data (Supplementary Fig. 15 and Supplementary Fig. 21). However, subclones B and C exhibit heterogeneity in the evolution stage two with loss of translocations, leading to different local genomic maps of BFB (Supplementary Fig. 21).

We further applied Ambigram to single-cell DNA data of gastric cancer mkn45^32^, which showed ten major subclones with distinct CN profiles (Supplementary Fig. 20b), to check for the presence of complex BFB events and the absence of intratumor heterogeneity in detected BFB. We independently detected five BFB events that demonstrated varying subclonal heterogeneity on five chromosomes (chr1, chr3, chr11, chr12, and chr15). The BFB events in chr1 have three and one FBIs, yielding eight and six BFB cycles in subclones A and M8, respectively (Supplementary Fig. 22). Genes *WDR47*, *TAF13*, and *TMEM167B* demonstrate two more CNs in subclone A than in M8. The BFB event in chr3 consists of four FBIs that split the local region of chr3 in subclone M9 into nine segments. Ambigram resolved the local genomic map that includes seven BFB cycles, which amplified the gene *CNOT10* (H5-H9) four times (Supplementary Fig. 23a). However, the local region of chr3 in subclone M4 is partitioned into five segments by two FBIs. Compared to the BFB event in M9, the M4 BFB path only contains three BFB cycles, with gene *CNOT10* (H5-H9) being diploid (Supplementary Fig. 23b). In subclone M8, the local genome of chr11 involves eight BFB cycles with four FBIs (Supplementary Fig. 24). Besides, the local region in subclone A shows eight BFB cycles with three FBIs. Finally, the genome region in subclone M6 undergoes three BFB cycles with two FBIs (Supplementary Fig. 25). The occurrence times of BFB cycles in subclones M8, A, and M6 progressively decrease, resulting in the CNs of the carrying genes *KIAA1549L*, *CD59*, *FBXO3*, *LMO2*, *CAPRIN1*, and *NAT10* also decreasing progressively. Regarding the BFB event in chr12, subclone M8 undergoes nine BFB cycles with four FBIs, leading to high amplification (CN ≥ 14) of genes *RASSF3*, *GNS*, *TBC1D30*, and *WIF1* (Supplementary Fig. 26). Compared to subclone M8, the local regions of both subclones M7 and M6 undergo seven BFB cycles missing one different FBI. (Supplementary Fig. 27). With fewer BFB cycles, the genes have less than 10 CNs. In the local region of chr15, subclones A and M7 contain five and three FBIs, and both of them undergo eight BFB cycles (Supplementary Fig. 28). Segments H2-H3 covering genes *SEMA4B*, *CIB1*, *GDPGP1*, *TTLL13*, and *NGRN* have nine and seven CNs in A and M7, respectively. *CRTC3* has five CNs in subclones A and M7. Segments H5-H8 covering genes *GABARAPL3*, *ZNF774*, and *IQGAP1* have thirteen and eight CNs in subclones A and M7, respectively.

### Ambigram resolves oncovirus integration by complex BFB path

Jia et al. have developed a conjugate graph-based algorithm FuseSV^37^ to decipher the local genomic map of oncovirus integrations for hepatocellular carcinomas (HCC)^38^. Here, we investigated the nine integration events in three HCC samples and identified seven events that had FBIs. We illustrated five instances of HBV interaction that may be interpreted by complex BFB events.

HBV integrations in chr1 of the HCC sample 101T occur on oncogene *CGN*. The VITs and SVs partition the local genome region and the virus genome into five segments (H1-H5 and V1-V5), respectively (Supplementary Fig. 29a–c). FuseSV inferred that the local genomic map includes one copy of alleles of normal order (H1-H5) and one copy of an allele with a human inversion and short HBV segments (Supplementary Fig. 29e). Ambigram revealed that the BFB event occurred in two stages (Supplementary Fig. 29d). In the first stage, chr1 undergoes two BFB cycles. The first BFB cycle occurs when a sister chromatid is replicated, and the H5 segment is fused with its reverse complement. Then the second BFB cycle starts when the double-strand breaks off at reverse segment $\overline{H4}$. A duplication is reproduced, and reverse segment $\overline{H4}$ is fused with segment H2. In the second stage, the HBV segments V3 and V4 are inserted into the area between the reverse segment $\overline{H4}$ and segment H2 through the integration of HBV, leading to the final local genomic map of this complex BFB event.

For the other four integration events (260T_chr1, 260T_chr10, 261T_chr5, and 261T_chr16), FuseSV came to the same conclusion that the local genomic map of virus integration is the deletion of human segments by inserting short linear or reversed HBV segments (Supplementary Fig. 30–33). FuseSV explained the high amplification of HBV fragments, such as possible ecDNAs or linear DNA outside the local genome^37^. With Ambigram, we showed the possibility of virus-induced complex BFB events. In summary, the human genome may integrate with the virus genome, and then the virtual contig formed undergoes several round BFB cycles on virus segments. In the next stage, another virus integration occurs to replace some segments on the BFB local genomic map (Supplementary Fig. 30–33).

**Fig. 4:**
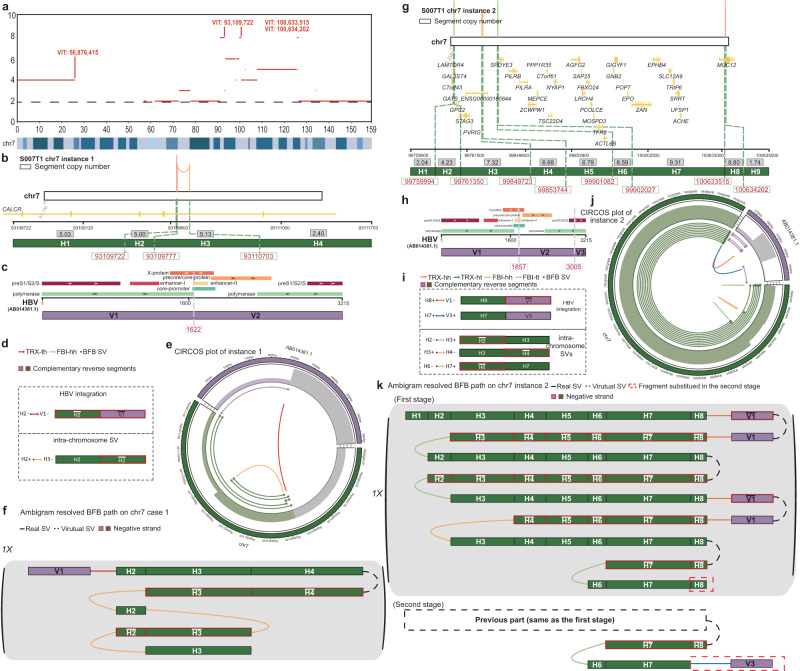
Two complex BFB instances involving HBV integration and genes *CALCR*, *MUC12*, *etc*., on chr7 of HCC S0007T1. **a** VITs and CN profiles of chr7 in S0007T1 - VIT positions are indicated by red arrows, and the CN profile is illustrated by red lines with the chr7 ideogram. **b** S0007T1 BFB instance 1 - the SV breakpoints split the local genome region into four segments. The light yellow and light red lines on the top denote the relative positions of SV breakpoints on chr7, and the gray box shows the average CN of segments. The middle layer shows gene annotation. **c** S0007T1 BFB instance 1 - the VIT breakpoint splits the virus genome into two segments. The top layer shows functional annotation. **d** S0007T1 BFB instance 1 - list of VITs, SVs, and the segments connected by them. **e** CIRCOS diagram of S0007T1 BFB instance 1. **f** S0007T1 BFB instance 1 - Ambigram resolved BFB path. Segment V1 of HBV is connected to segment H2 on chr7. Then three BFB cycles happen on the HBV-integrated local genomic map. **g** S0007T1 BFB instance 2 - the SV breakpoints split the local genome region into nine segments. The vertical lines on the top denote the relative positions of SV breakpoints on chr7, and the gray box shows the average CN of segments. The middle layer shows gene annotation. **h** S0007T1 BFB instance 2 - the VIT breakpoints split the virus genome into three segments. The top layer shows functional annotation. **i** S0007T1 BFB instance 2 - list of VITs, SVs, and the segments connected by them. **j** CIRCOS diagram of S0007T1 BFB instance 2. **k** S0007T1 BFB instance 2 - Ambigram resolved BFB path. This complex BFB event consists of two stages. In the first stage, the HBV reverse segment $\overline{V1}$ is connected to segment H8 on chr7. Then the HBV-integrated local genomic map undergoes six BFB cycles. In the second stage, another HBV integration connects segment H7 to segment V3, which replaces the tail of the BFB path.

Next, we investigated an HCC sample S0007T1^39^ that contained four VITs and stair-like CN profiles on chr7 (Fig. 4a). We found two possible complex BFB instances. In the first BFB instance, the SV and VIT breakpoints split the local human genome and the virus genome region into four and two segments, respectively (Fig. 4b-d). Ambigram inferred that segment V1 of HBV is first connected to segment H2 on chr7, leading to a truncation of the gene *CALCR*. Then three BFB cycles occur in the HBV-integrated local genomic map (Fig. 4f). In the second BFB instance, the SV and VIT breakpoints split the local human genome and the region of the viral genome into nine and three segments (Fig. 4g-i). We inferred that this complex BFB event consists of two stages. In the first stage, the HBV segment H8 on chr7 is connected to the reverse segment $\overline{V1}$. Then the HBV-integrated local genomic map undergoes six BFB cycles, leading to the gain of CN of genes *SPDYE3*, *AGFG2*, *MUC12*, *etc*. In the second stage, another HBV integration connects segment H7 to segment V3, which replaces the tail of the BFB path and indicates the end of the HBV integration BFB event (Fig. 4i).

Furthermore, we found that the BFB mechanism might mediate three HPV integration events in cervical cancer. HELA and SIHA cell lines are two well-known cervical cancer cell lines associated with HPV16 interactions^40^. Ambigram detects an integration of HPV in chr3 of the HELA cell line that truncates the gene *FHIT* (Supplementary Fig. 34). Tumor suppressor alterations *FHIT* are common signs of carcinogenesis^41^. For the SIHA cell line, the complex BFB that involves the integration of HPV into chr13 leads to the amplification of the gene *DACH1*, *MZT1*, *BORA*, *DIS3*, and *PIBF1* (Supplementary Fig. 35). *BORA*, *DIS3*, *PIBF* have been reported to be carcinogenic in multiple cancers^42–44^. Ambigram also detected a complex BFB event involving HPV integration in chr19 of the cervical cancer sample^45^, causing amplification of the ZNF and LILR gene families (Supplementary Fig. 36). ZNFs are oncogenic in cancer progression^46^.

### BFB may drive the genome reorganizations of *Homo Sapiens*

**Fig. 5:**
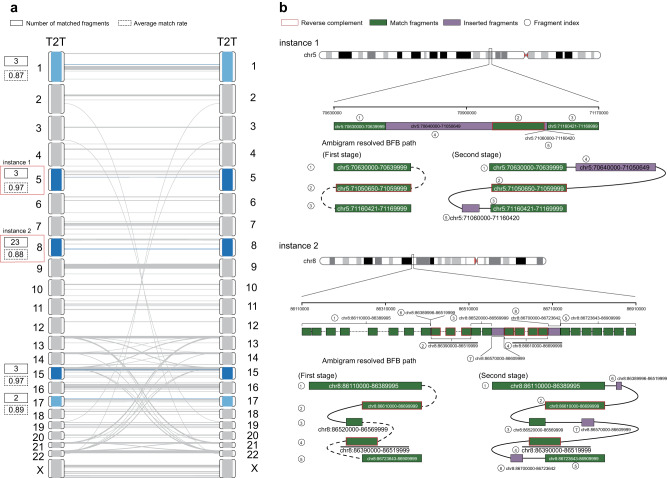
Two T2T local genomic regions where the genomic reorganization may have been carried out by the BFB mechanism during evolution. **a** The genome synteny analysis of T2T. Lines and curves indicate match fragments on the complete human genome, which are aligned by MUMmer4^64^. Gray regions have few reverse matches, which BFB cannot explain. Five local regions colored blue have considerable reverse matches and are further aligned with themselves by our algorithm (Supplementary Methods, Algorithm 5). Regions with darker blue have a higher match rate or more match fragments. Red boxes represent two local regions possibly derived from complex BFB events. **b** Ambigram resolves BFB evolution processes on T2T local genome regions of chr5 and chr8, respectively. The first instance consists of two stages. In the first stage, the local region undergoes two BFB cycles, contributing to two forward fragments and one reverse fragment. In the second stage, two fragments colored purple are inserted into the BFB path, composing the final genomic sequence in T2T. Similarly, the second instance consists of two stages too. Firstly, the local region on chr8 undergoes three BFB cycles, leading to three forward fragments and two reverse segments. Moreover, three fragments colored purple are inserted into the BFB path, contributing to the final genomic sequence in T2T.

Recently, Nurk et al. have published a complete sequence of a human genome (T2T-CHM13) that fixes gaps and issues in the reference genome GRCh38^47^. The complete T2T genome facilitates the possibility of inferring the evolution process of *Homo sapiens*. Here, we conducted a genome synteny analysis of T2T (Fig. 5a and Methods). As a result, we identified two local genomic regions in which the BFB mechanism may have carried out genomic reorganization during the evolution into *Homo sapiens* (Fig. 5b). The first region is chr5:70,630,000–71,169,999, which can be divided into five genomic fragments in sequence. The first and second genomic fragments are reverse complementary to each other, and the third fragment is the same as the first. Ambigram resolved the BFB evolution process in two stages. In the first stage, the local region undergoes two BFB cycles, contributing to two forward fragments and one reverse fragment. In the second stage, two purple fragments are inserted into the BFB path, composing the final genomic sequence in T2T. The BFB mechanism may also explain the formation of the genome region that spans chr8:86,110,000–86,909,999, involving eight genomic fragments. The green fragments are either reverse complements or forward-matching sequences. Similarly, Ambigram deciphers that the BFB evolution process has two stages. First, the green fragments are concatenated with three BFB cycles. Second, the purple fragments are inserted in the BFB path, which constitutes the final genomic sequence in T2T.

### Ambigram detects recurrent FBIs and complex BFBs in 1000GP

We applied Ambigram to 923 healthy whole genome data from the 1000 Genome Project (1000GP)^48^ to investigate the presence of germline BFB. We observed 85 recurrent FBI polymorphisms (occurring in more than 5% of samples in the cohort) in 1000GP, directly truncating 32 genes (Supplementary Fig. 37a). The top three genes that carry FBI polymorphisms are *APP*, *C4BPA*, and *SUGCT* with a frequency of 91%, 52%, and 49%. Among the 32 FBI hotspot genes, 11 genes are associated with BFB events involving at least two FBIs (Supplementary Fig. 37b). The top three genes are *APP*, *PCDH15*, and *CNTNAP5* with a frequency of 49%, 33%, and 26%.

### Ambigram detects CHD-related recurrent FBIs and complex BFBs

Congenital heart disease (CHD) is a substantial cause of neonatal death. As large chromosomal rearrangements and copy number variations have been reported to be the genetic pathogenesis of CHD^49–51^, the complex BFB events are likely related to CHD. Here, we investigated the possible complex BFB events from 330 CHD probands and 612 of their parents^52^, with 923 whole genome data from 1000GP as unaffected controls^48^.

**Fig. 6:**
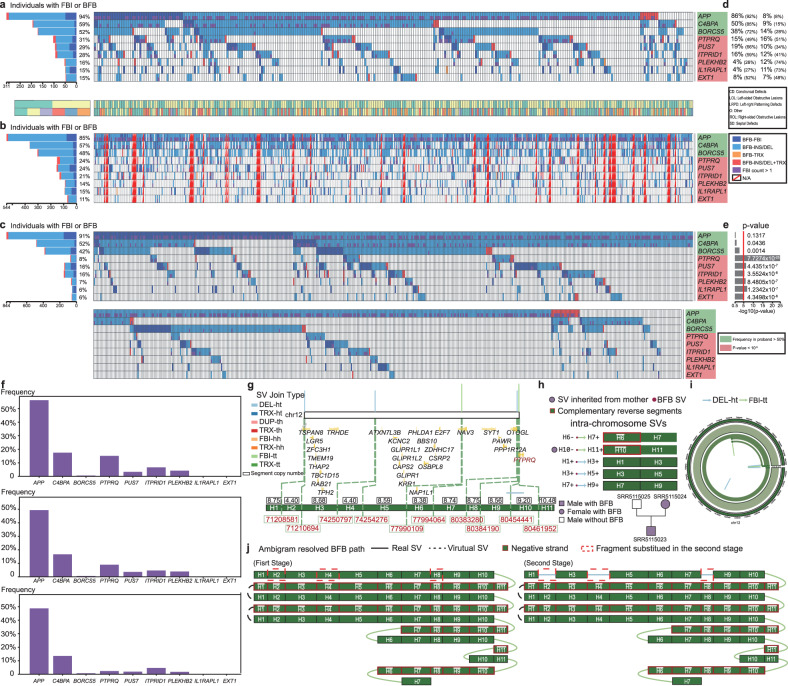
BFB analysis in CHD probands, relatives, and controls. **a** The recurrent FBI landscape in the CHD probands with gender and phenotypic information. The landscape is plotted by https://bio.oviz.org/demo-project/analyses/landscape^68^. **b** The recurrent FBI landscape in the CHD parents. The CHD probands are vertically aligned with their parents. **c** The recurrent FBI landscape in the 1000GP cohort. **d** The ratio of recurrent FBI polymorphisms inherited or de novo. **e** The enrichment *p* value of genes carrying FBI polymorphisms in probands against 1000GP controls. We used the one-sided Chi-square test without adjustments. **f** The frequency of genes carrying BFB event (FBI count > 1) in CHD probands, relatives, and controls. **g** The FBI and SV breakpoints split the local genome region of chr12 into 11 segments. The vertical lines above show the positions of SVs on chr12, and the middle layer shows gene annotation. The black box shows the average CN of segments. **h** List of SVs and the segments connected by them. The head-to-tail (ht) deletion and tail-to-tail (tt) FBIs are colored light blue and light green, respectively. The reverse complementary segments have a red border. The family tree on the right shows the inherited relationship between parents and the child, and samples colored purple undergo BFB events in the local region, while the sample without color does not have signs of a BFB event. The SV labeled with a purple circle is the FBI inherited from the mother sample SRR5115024 to the proband sample SRR5115023. **i** CIRCOS diagram of the complex BFB. **j** Ambigram resolved the BFB path in sample SRR5115023. This complex BFB event consists of two stages. In the first stage, the local region on chr12 undergoes seven BFB cycles. In the second stage, three SVs happen between segments H1 and H3, H3 and H5, and H7 and H9, deleting parts of the BFB path.

First, we observed recurrent FBI polymorphisms (occurring in >5% of samples in a cohort) across CHD probands (n=71), their parents (*n* = 89), and 1000GP controls (*n* = 85). The top three genes that carry recurrent FBI polymorphisms are *APP*, *C4BPA*, and *BORCS5* (Fig. 6a–c and Supplementary Table 1). The frequency of FBIs occurring in *APP* is 94%, 85%, and 91% in the proband, relative, and control cohorts, respectively. The prevalence of FBIs in *C4BPA* is 59%, 57%, and 52% in the proband, relative, and control groups, respectively. The occurrence of FBIs harboring in *BORCS5* is 31%, 48%, and 42% in the proband, relative, and control cohorts, respectively. Among these detected FBIs, most are associated with BFB events involving at least two FBIs (Fig. 6f) or complex BFB events involving insertion or deletion (Fig. 6a–c). These recurrent FBI polymorphisms can either be inherited or de novo, with the inherited ones predominating (Fig. 6d). 91%, 85%, and 72% of the recurrent FBI polymorphisms occurring in *APP*, *C4BPA*, and *BORCS5* probands are inherited from their parents. Although *APP* and *C4BPA* are associated with heart disease (Supplementary Table 2), we showed that these recurrent FBI polymorphisms in the two genes were non-causative factors of CHD.

Next, we detected six genes carrying recurrent FBI polymorphisms that significantly (*p* value <1e-5, Chi-square test) enriched in CHD probands compared to healthy controls from 1000GP. These genes are *PTPRQ*, *PUS7*, *ITPRID1*, *PLEKHB2*, *IL1RAPL1*, and *EXT1* (Fig. 6e and Supplementary Table 1). Around 30% population in CHD probands exhibit FBIs or complex BFB events that harbor the three genes (*PTPRQ*: 31%, *PUS7*: 29%, and *ITPRID1*: 28%). In their relatives, the frequencies are 24%, 24%, and 21% for *PTPRQ*, *PUS7*, and *ITPRID1*, respectively. However, the frequency of occurrences decreases significantly in 1000GP controls, especially for *PTPRQ* with 8% frequency (*p* value: 7.7274e-25). Then, around 15% population in CHD probands exhibit FBIs or complex BFB events harboring the three genes (*PLEKHB2*: 16%, *IL1RAPL1*: 15%, and *EXT1*: 15%). In their relatives, the frequencies are 14%, 15%, and 11%. In the controls, the frequencies are 7%, 6%, and 6%. The above results suggest that *PTPRQ*, *PUS7*, *ITPRID1*, *PLEKHB2*, *IL1RAPL1*, and *EXT1* might carry recurrent FBI or BFB polymorphisms associated with CHD. *PTPRQ*, *PLEKHB2*, *IL1RAPL1*, and *EXT1* are predicted to be involved in biological processes such as heart and large blood vessel development, as well as cardiac phenotypes such as arrhythmias, valve diseases, and cardiomyopathy (Supplementary Table 2). The proportion of CHD-related de novo recurrent FBI polymorphisms has increased or even dominated (Fig. 6d). For example, 74% and 73% of recurrent FBI polymorphisms occurring in *PLEKHB2* and *IL1RAPL1* of the proband occur de novo.

Finally, we demonstrated how Ambigram constructed the local genomic map of a complex BFB event associated with *PTPRQ* in the male proband SRR5115023. This complex BFB event involved two FBIs and three deletions. Ambigram inferred that the formation of complex BFB consists of two stages (Fig. 6g–j). In the first stage, the local region on chr12 undergoes seven BFB cycles. In the second stage, three deletions occur. An FBI occurs in *PTPRQ*, which may cause its loss of functionality. The pedigree analysis showed that the FBI which links segments H10 and H11 in the proband SRR5115023 is inherited from his mother SRR5115024. For the other five genes *APP*, *C4BPA*, *BORCS5*, *PUS7*, and *ITPRID1*, we also present a complex BFB event and its hereditary situation separately (Supplementary Fig. 38–42).

## Discussion

BFB is a complex rearrangement worth noting that leads to tumor malignancy. Supported by its two hallmarks, stairs-like CN gains and FBI enrichment, most studies claimed the occurrence of BFB events without resolving the exact BFB path, that is, the evolution process of BFB^3–9,11,12,16^.

In recent years, modeling the “palindrome” or “ambigram” nature of BFB-induced local genomic map given by CN profiles or FBIs from a mathematical point of view has become a growing interest (Table 1). Kinsella et al. claimed the first formal mathematical formulation of the BFB mechanism: the BFB segment path is a palindrome string - successive inverted prefix duplications of a string, e.g., abccba^22^. They proposed an exponential-time algorithm to determine whether a given copy number count vector fits into a path of the BFB palindrome^22^. Zakov et al. then developed BFBFinder to speed up Kinsella’s model to linear time, which fits the copy number count vector by folding BFB palindrome collections^23^. BFBFinder was further enhanced by making the model tolerant to noisy copy number count vectors and predicting all possible segment count vectors and associated BFB architectures^24^. Greenman et al.^25^ modeled the evolution space of BFB cycles with 2-d trees and stochastic folding, investigating the space of possible BFB paths. Greenman et al.^26^ then discussed the complexity of rearrangement evolution, including BFB cycles with an infinite-site model. Recently, computational pipelines to resolve complex structure rearrangement including complex BFB have emerged (Table 1). AmpliconArchitect + AmpliconClassifier^19^ and AmpliconReconstructor^20^ handle short reads or optical mapping data to detect focal amplicons. Shale et al. introduced LINX which utilizes clustering, chaining, and annotating schemes to resolve complex BFB in somatic genome rearrangements^21^.

Here, we proposed a graph algorithm, Ambigram, which has the following advantages over the above rivals in detecting BFBs and reconstructing their local genomic maps during evolution. First, Ambigram detects complex BFB events that carry deletion, duplication, insertion, and translocation, while mathematical models rely on the perfect BFB hypothesis that merely has FBIs (Tables 1 and 2). Second, Ambigram is compatible with PE, 10x, PB, ONT, and OM protocols. We showed that incorporating read linkage evidence from linked read, long read, or optical mapping data facilitated the efficacy of BFB reconstruction on in silico and real benchmarking. We also showed that Ambigram is robust with various sequencing depths and tumor purities. However, other models have only validated their efficacy in outdated aCGH or PE data, except AmpliconReconstructor supports OM data (Table 1). Third, Ambigram is comparable with single-cell data (Table 1). Ambigram can efficiently work out a series of BFB paths with subclone annotations that share similar DNA patterns whilst differentiating their unique SVs and CNs. We deciphered the intratumor heterogeneity on complex BFB events from single-cell sequencing of melanoma and gastric cancer data. Fourth, Ambigram interprets oncovirus integration (Table 1). We have demonstrated that the BFB cycle mechanism may mediate seven HBV integrations in four liver cancers and three HPV integrations in three cervical cancers. The BFB cycles truncate the tumor suppressor *FHIT* or amplify the oncogene *MUC12*, *BORA*, *DIS3*, *PIBF*, or *ZNFs*.

Although BFB is proposed as a mechanism that leads to complex genome rearrangements in cancers. We detected BFB signals from non-cancer data (Table 4). First, we suggested that two genome reorganizations of *Homo Sapiens* during evolution may be carried out by the BFB mechanism after investigating the complete human genome. Second, Ambigram discovered 85 recurrent FBI or complex BFB polymorphisms (occurring in more than 5% of samples in the cohort) in 1000GP, directly truncating 32 genes, including *APP*, *C4BPA*, and *SUGCT*. Third, Ambigram detected FBIs or BFBs in complex diseases. In a case study of 330 CHD probands and 612 relatives, with samples from 1000GP as controls, we observed recurrent FBI or complex BFB polymorphisms in the three cohorts. The three genes that are most frequently implicated in FBIs are *APP*, *C4BPA*, and *BORCS5*. We find that *PTPRQ*, *PUS7*, *ITPRID1*, *PLEKHB2*, *IL1RAPL1,* and *EXT1* that carry FBI or complex BFB polymorphisms are significantly (*p* value < 1e-5) related to CHD. The observed FBI or complex BFB polymorphisms can either be inherited or occur de novo.

**Table 4:** Summary of datasets to investigate potential BFB event with Ambigram

Dataset	Data Type	Protocol	Data Format	Ambigram
COLO829	Melanoma	SC	WGS	*✓*
mkn45	Gastric cancer	SC	WGS	*✓*
101T	Liver cancer+HBV	PE	SV+CN	*✓*
260T	Liver cancer+HBV	PE	SV+CN	*✓*
261T	Liver cancer+HBV	PE	SV+CN	*✓*
S0007T1	Liver cancer+HBV	PE	WGS	*✓*
HELA	Cervical cancer+HPV	PE	SV+CN	*✓*
SIHA	Cervical cancer+HPV	PE	SV+CN	*✓*
CRR046045	Cervical cancer+HPV	PE	WGS	*✓*
1000GP	Normal	PE	WGS	*✓*
CHD	Heart disease	PE	WGS	*✓*

*✓*means the BFB events are detected and resolved. *BFB* breakage-fusion-bridge. *SC* single cell, *PE* paired-end, *WGS* whole genome sequencing, *SV+CN* called structure variation and copy number in the local genome.

There are some concerns that we need to address. First, despite the fact that the time complexity of Ambigram with respect to the number of segments (*n*) is *O*(*n*^3^) (Methods and Supplementary Fig. 43a), we discovered that the worst instance scenario rarely occurs and the segment count is low in real BFB scenarios. In this study, we resolved 27 real BFB events and found that the minimum, Q1, median, Q3, and maximum segment count were 4, 7.5, 9, 11, and 18, respectively. The FBI count reached a median value of 2 and a maximum value of 10, the total CN of all segments reached a median value of 51 and a maximum value of 302, and the largest segment CN had a median value of 6 and a maximum value of 20 (Supplementary Fig. 43b). When running on the Ubuntu 20.04 platform with a 12th Gen Intel(R) Core(TM) i7-12700F (20 CPUs) and 32 GB RAM, the real trials were completed in 40 seconds with SV and CN setup as inputs (Supplementary Fig. 43c). Despite the relatively small segment count, these BFB events caused focal amplicons with high CN values. Additionally, for the 410 simulated BFB paths with varying numbers of segments (ranging from 6 to 15), FBIs (ranging from 2 to 7), total segment CN (ranging from 13 to 105), and largest segment CN (ranging from 3 to 11). Ambigram completed the task within ten seconds (Supplementary Fig. 43b, d).

Second, Ambigram is a computational model that resolves the exact local genome structures from possible complex BFB candidates. Even though we benchmarked Ambigram with six in silico BFB events, two gold-standard real BFB events, and three silver-standard BFB events that were originally inferred by state-of-the-art tools (Tables 2 and 3). The biological insight and the underlying evolution hypothesis derived from Ambigram still need independent validation from biological experiments. However, Ambigram is a cost-effective tool that can help scientists screen possible complex BFB hypotheses for subsequent wet-lab experiments to decipher novel insights on tumor and complex disease development.

## Methods

### Problem formulation

#### Segments and junctions

A *fold-back inversion* (FBI), the hallmark of BFB, serves as a linkage to connect two *segments* that are reverse complements in the reference genome. Thus, an FBI consists of two *breakpoints*, corresponding to one end of the two segments. FBI breakpoints from sequencing data partition the relevant local genome into an ordered set of segments *S* = 〈*s*_1_, *s*_2_,...,*s*_*n*_〉. Denote the reverse complement segment of *s*_*i*_ as $\overline{{s}_{i}}$; the reverse complements of *S* are often attributed to the BFB event. Each segment *s*_*i*_ and its reverse complement segment $\overline{{s}_{i}}$ can occur multiple times in a BFB event, and denote the number of occurrences or the copy number (CN) as *c*(*s*_*i*_).

Meanwhile, the linkage connecting segment *s*_*i*_ and its reverse complement $\overline{{s}_{i}}$ is referred to as *FBI junction*, denoted $({s}_{i},\overline{{s}_{i}})$. Besides, the CN of $({s}_{i},\overline{{s}_{i}})$ derived from the sequencing data is labeled $c({s}_{i},\overline{{s}_{i}})$. Consequently, denote a set of FBI junctions involved in a BFB event as *J*, where ∣*J*∣ = *m*.

#### BFB paths

We term the sequence of segments *s*_1_*s*_2_...*s*_*n*_ as *reference path R*, the original genome sequence before BFB occurs. Based on the mechanism of BFB (Fig. 1a), each BFB cycle undergoes two operations: (1) *fusion* of two sister chromatids due to the lack of telomeres during DNA replication; (2) *breakage* on the fused bridge of two centromeres in the anaphase. Starting with the reference path (*R*), a *BFB path* (*P*) is the local genomic map obtained after a sequence of fusion and breakage operations. Since a BFB path has a palindromic suffix, it is called a palindrome-like path. In the next cycle, *P* is fused with its reverse complementary path $\overline{P}$ derived from DNA replication. Here is an illustration of the BFB event with FBI junctions *J* occurring on *R* (Supplementary Fig. 44a):

$$\begin{array}{rcl} &&\hskip -160pt R=s_1s_2...s_n\\ &&\hskip -140pt\mathop{\longrightarrow}\limits^{{{{{{{\rm{Fusion}}}}}}}} R|{{\overline{R}}}=s_1s_2...s_n|{{\overline{s_n...s_2s_1}}}\\ && \hskip -142pt\mathop{\longrightarrow}\limits^{{{{{{{\rm{Breakage}}}}}}}} P_1=s_1s_2...s_n|\overline{s_{n}...s_{a+1}s_a}\\ &&\hskip -30pt {\overline{s_{a-1}...s_2s_1}},\quad\quad\quad\quad 1 \leq a\leq n\, {{{{{{\mathrm{and}}}}}}}\, (s_a,{{\overline{s_a}}})\in J\\ && \hskip -70pt\mathop{\longrightarrow}\limits^{{{{{{{\rm{Fusion}}}}}}}} s_1s_2...s_n|\overline{s_n...s_{a+1}s_a}|s_as_{a+1}...s_n|{{\overline{s_n...s_2s_1}}}\\ && \hskip -95pt\mathop{\longrightarrow}\limits^{{{{{{{\rm{Breakage}}}}}}}} P_2=s_1s_2...s_n|\overline{s_n...s_{a+1}s_a}|s_as_{a+1}...s_b\\ && \hskip -10pt {s_{b+1}s_{b+2}...s_n|{{\overline{s_n...s_2s_1}}}},\quad a\leq b \leq n\, {{{{{{\mathrm{and}}}}}}}\, (s_b,{{\overline{s_b}}})\in J\\ && \hskip 10pt \mathop{\longrightarrow}\limits^{{{{{{{\rm{Fusion}}}}}}}} P_2|{{\overline{P_2}}}\hfill\\ && \hskip -52pt\mathop{\longrightarrow}\limits^{{{{{{{\rm{Breakage}}}}}}}} P_3=s_1s_2...s_n|\overline{s_n...s_{a+1}s_a}|s_as_{a+1}...s_b|\overline{s_b...s_{c+1}s_c}\\ && \hskip 30pt {\overline{s_{c-1}...s_{a+1}s_a}|s_as_{a+1}...s_n|{{\overline{s_n...s_2s_1}}}},\quad a \leq c\leq b\, {{{{{{\mathrm{and}}}}}}}\, (s_c,{{\overline{s_c}}})\in J\\ && \hskip 10pt \mathop{\longrightarrow}\limits^{{{{{{{\rm{Fusion}}}}}}}} \ldots \hfill\end{array}$$

As shown above, each BFB cycle involves an FBI junction, while an FBI junction may occur more than once. For example, $({s}_{a},\overline{{s}_{a}})$ may occur in two consecutive BFB cycles when *a* = *b* (Supplementary Fig. 44b). The FBI junction $({s}_{i},\overline{{s}_{i}})$ in each BFB cycle, where fusion happens, corresponds to either FBI ${s}_{i}| \overline{{s}_{i}}$ or FBI $\overline{{s}_{i}}| {s}_{i}$ on the BFB path obtained after breakage. Each path has a palindromic suffix symmetric about the breakpoint where the FBI happens. As a result, we can get a BFB path with all *m* FBI junctions, which is the final palindrome-like path derived from the BFB event.

#### Mathematical formulation of BFB

Based on the palindromic properties of the BFB paths, we have formulated the following definitions:

1. A *mono-chainm*(*a*, *b*) = *s*_*a*_*s*_*a*+1_...*s*_*b*−1_*s*_*b*_ or $\overline{{s}_{b}{s}_{b-1}...{s}_{a+1}{s}_{a}}$ is a list of consecutive segments bounded by FBI breakpoints, where *a* and *b* are the indices of the start and end segments that meet *a* ≤ *b*. The *length* of mono-chain *m*(*a*, *b*) is the number of segments the mono-chain spans, defined as *b* − *a* + 1.
2. A *loop*$l(a,b)={s}_{a}{s}_{a+1}...{s}_{b}| \overline{{s}_{b}{s}_{b-1}...{s}_{a}}$ or $\overline{{s}_{b}{s}_{b-1}...{s}_{a}}| {s}_{a}{s}_{a+1}...{s}_{b}$ is a concatenation of two reverse complementary mono-chains, where *a* ≤ *b*. The *length* of loop *l*(*a*, *b*) is half of the number of segments that the loop spans, defined as *b* − *a* + 1.
3. An *entity* is a collective name that represents either a mono-chain or a loop, i.e., *e*(*a*, *b*) = *m*(*a*, *b*) or *l*(*a*, *b*). An entity *e*(*a*_1_, *b*_1_) is the *child entity (mono-chain or loop)* of the entity *e*(*a*_2_, *b*_2_), if and only if (*a*_1_ = *a*_2_*orb*_1_ = *b*_2_) and *b*_1_ − *a*_1_ < *b*_2_ − *a*_2_. Consequently, *e*(*a*_2_, *b*_2_) is *parent entity (mono-chain or loop)* of *e*(*a*_1_, *b*_1_).

Recall that a BFB path is a palindrome-like path obtained after a sequence of fusion and breakage operations on the reference path. As proved in Supplementary Methods, any BFB path can be constructed by integrating mono-chains and then inserting loops.

#### BFB DAG and BFB tree

With the above definitions and proofs in Supplementary Methods, BFB paths can be represented by a DAG *G*(*V*, *E*), where *V* is an entity set and *E* consists of directed edges that connect parent and child entities (Supplementary Fig. 45a). The DAG is named *BFB DAG*, which is a superset of a *BFB tree*$T(V,E{\prime} )$, where $E{\prime} \subseteq E$. A BFB tree connects all entities in *V* and uniquely corresponds to a BFB path (Supplementary Fig. 44c). Considering a DAG may have many BFB trees, we simplify the search space by only using topological orders to construct BFB trees and compose BFB paths (Supplementary Fig. 45b). As a result, given mono-chains and loops, we can construct a BFB DAG by connecting each pair of parent and child entities and reconstruct BFB paths by assembling the entities on BFB trees derived from the BFB DAG in topological orders (Supplementary Methods, Algorithm 2 and 3).

#### Adjusting CN configuration

As vibration occurred in sequencing, the measured read depth may comprise noises^37^. The CN configuration of segments and junctions may deviate from the underlying staircase-like CN pattern, which is the other hallmark of BFB. Therefore, we utilize integer linear programming (ILP) to derive an estimated CN configuration that best fits the BFB CN pattern by minimizing all differences between the observed and estimated CNs. As a result, given the segments, FBI junctions, and CN profiles in a tumor sample, we can calculate the estimated CNs of the entities by ILP. Then we construct a BFB DAG by connecting all pairs of parent and child entities with a CN larger than 0. Finally, we reconstruct the BFB paths by connecting the entities based on topological orders in the BFB DAG (Fig. 1c).

### The algorithm of Ambigram

#### Definition of SV, CSV, and BFB

Genomic structural variations (SVs) are the rearrangement of large DNA segments over 50 base pairs^53^. An SV serves as a junction to connect two segments that are not adjacent to each other in the reference genome. SV thus consists of two breakpoints corresponding to one end of the two segments. If the two segments are from the same chromosome, the type of junction can be head-to-tail deletion (DEL-ht), tail-to-head duplication (DUP-th), head-to-head fold-back inversion (FBI-hh), and tail-to-tail fold-back inversion (FBI-tt) (Supplementary Fig. 46a). If the two segments are from different chromosomes, the junction type can be head-to-tail translocation (TRX-ht), tail-to-head translocation (TRX-th), head-to-head translocation (TRX-hh), and tail-to-tail translocation (TRX-tt) (Supplementary Fig. 46b).

Most SVs do not occur independently but are caused by some catastrophic events or are mediated by one biological mechanism in a cell, leading to a combination of different types of SVs that links segments from different positions of the genome^15^. We term such an SV set containing at least two SVs as *complex structure variation* (CSV). If the biological mechanism is a series of BFB cycles, the resulting CSV contains merely a group of FBIs with two same breakpoints^14–16^. We term such a CSV as *perfect BFB*. Studies reported that deletion, duplication, insertion, and translocation could be involved during BFB cycles^13,21^, we term this CSV as *complex BFB*.

#### Finding BFB candidate SV sets

Given SVs derived from the whole genome sequencing data, we adopted a breadth-first search algorithm to group the SVs with near breakpoints into sets, such that SVs involved in the same CSV event are grouped into the same set (Supplementary Methods, Algorithm 1). We consider the SV set containing more than one FBI as the *BFB candidate SV set*, and the local genome regions involved with this set undergo a BFB event.

#### Estimating the CN configuration for the candidate BFB

##### Converting depth to observed CN

Given a candidate BFB, we obtained the observed CNs of related segments and junctions from the BFB candidate SV set based on tumor purity *ρ* and read depths called from the BAM file, as suggested in FuseSV^37^. Assume that the average ploidy of the tumor and normal is *P*_*t*_ and *P*_*n*_, respectively. The average depth of one BFB path *D*_*h*_ is ${D}_{h}=\frac{{D}_{g}}{\rho \times {P}_{t}+(1-\rho )\times {P}_{n}}$, where *D*_*g*_ represents the average sequencing depth of whole genome. With the average depth of one BFB path *D*_*h*_, we convert the read depths *D* covered in segments and junctions into their CN *C* by $C=\frac{D}{{D}_{h}}$.

##### Explanation of formulas

Given a BFB event involved with a set of segments *S* = 〈*s*_1_, *s*_2_,..., *s*_*n*_〉, Formula (1) defines the objective of ILP to minimize the disparities between the observed and estimated CNs of segment *s*_*i*_ and FBI junction $({s}_{i},\overline{{s}_{i}})$, which are denoted by *ε*_*i*_ and *ξ*_*i*,*i*_ for ∀*i* ∈ {1, 2,..., *n*}.

1 $$\min \mathop{\sum }\limits_{i=1}^{n}{\varepsilon }_{i}+\mathop{\sum }\limits_{i=1}^{n}{\xi }_{i,i}$$

We can obtain the observed CNs of segment *s*_*i*_ and FBI junction $({s}_{i},\overline{{s}_{i}})$ from the sequencing data, which are *c*(*s*_*i*_) and $c({s}_{i},\overline{{s}_{i}})$, respectively. Based on the mathematical formulation of BFB, the estimated CNs of the segments and junctions correspond to the number of times the entities occur in the resultant BFB path, which is defined as the CN of entity *e*(*a*, *b*), denoted by *c*_*e*_(*a*, *b*). Additionally, the CNs of mono-chain *m*(*a*, *b*) and loop *l*(*a*, *b*) are denoted by *c*_*m*_(*a*, *b*) and *c*_*l*_(*a*, *b*), respectively. Hence, we have the following ILP formulas that define two CN disparities respectively.

On the one hand, we denote the sets of mono-chains and loops that contain segment *s*_*i*_ by *M*_*i*_ = {*m*(*a*, *b*)∣*a* ≤ *i* ≤ *b*} and *L*_*i*_ = {*l*(*a*, *b*)∣*a* ≤ *i* ≤ *b*}, respectively. In addition, the corresponding sets of estimated CNs are defined as ${C}_{m}^{i}=\{{c}_{m}(a,b)| m(a,b)\in {M}_{i}\}$ and ${C}_{l}^{i}=\{{c}_{l}(a,b)| l(a,b)\in {L}_{i}\}$. A mono-chain *m*(*a*, *b*) ∈ *M*_*i*_ contributes *c*_*m*_(*a*, *b*) to the estimated CN of *s*_*i*_, while a loop *l*(*a*, *b*) ∈ *L*_*i*_ contributes 2 ⋅ *c*_*l*_(*a*, *b*) to the estimated CN of *s*_*i*_. Therefore, we have formula (2) to define the CN differences for ∀*s*_*i*_ ∈ *S*:

2 $$-{\varepsilon }_{i}\le \left(\mathop{\sum}\limits_{c\in {C}_{m}^{i}}c+\mathop{\sum}\limits_{c{\prime} \in {C}_{l}^{i}}2\cdot c{\prime} \right)-c({s}_{i})\le {\varepsilon }_{i}$$

On the other hand, we denote the set of mono-chain pairs, which are connected by FBI junction $({s}_{i},\overline{{s}_{i}})$, as *P*_*i*_ = {[*m*(*a*_1_, *b*_1_), *m*(*a*_2_, *b*_2_)]∣*b*_1_ − *a*_1_ > *b*_2_ − *a*_2_ and (*a*_1_ = *a*_2_ = *i or b*_1_ = *b*_2_ = *i*)}. Meanwhile, the set of loops that contains the FBI junction is defined as *L*_*i*,*i*_ = {*l*(*a*, *b*)∣*a* = *i or b* = *i*}. We suppose that a mono-chain pair in *P*_*i*_ contributes their average to the estimated CN of $({s}_{i},\overline{{s}_{i}})$. A loop *l*(*a*, *b*) contributes *c*_*l*_(*a*, *b*) to the estimated CN of $({s}_{i},\overline{{s}_{i}})$. Hence, we define two CN sets ${C}_{p}^{i}=\{\frac{1}{2}\cdot [{c}_{m}({a}_{1},{b}_{1})+{c}_{m}({a}_{2},{b}_{2})]\left|\right.[m({a}_{1},{b}_{1}),m({a}_{2},{b}_{2})]\in {P}_{i}\}$ and ${C}_{L}^{i,i}=\{{c}_{l}(a,b)| l(a,b)\in {L}_{i,i}\}$. As a result, we have formula (3) to define the CN disparities for all FBI junctions $({s}_{i},\overline{{s}_{i}})$, where ∀*i* ∈ {1, 2,...,*n*}:

3 $$-{\xi }_{i,i}\le \left(\mathop{\sum}\limits_{c\in {C}_{p}^{i}\cup {C}_{L}^{i,i}}c\right)-c({s}_{i},\overline{{s}_{i}})\le {\xi }_{i,i}$$

Additionally, we introduce several default constraints to the CN configuration of mono-chains and loops so that the adjusted CN profiles match the underlying BFB CN pattern (Supplementary Methods). Firstly, the ILP constraints guarantee each entity (except for the reference path) must have a parent entity in the result so that all entities generated by ILP can be integrated into a BFB DAG. Besides, we incorporate constraints on the CN differences of entities of multiple subclones for single-cell data to reconstruct several BFB paths with some similar entities. Furthermore, since the linked and long sequencing techniques deliver long-distance read linkages, which provide evidence of the possible connections among segments for a real BFB path, we append a linkage constraint if the linked or long reads are available.

#### Constructing BFB DAG

After calculating CNs of mono-chains and loops by ILP, we can construct a BFB DAG, where a vertex represents an entity and a directed edge links an entity to its child entity. Besides, each vertex is assigned a CN that indicates how many times the entity repeats in the resultant BFB path. Here are the main steps for creating a BFB DAG:

1. Choose all mono-chains and loops with CN larger than 0 as vertices, and sort all the entities in decreasing order of length.
2. For each mono-chain, link it to its child mono-chains and loops.
3. For each loop, if a mono-chain is a child of the loop, then link the loop to the mono-chain.
4. For each loop, link it to its child loops.

Furthermore, we extract a BFB tree that uniquely corresponds to a BFB path from the DAG (Supplementary Fig. 44c). As a result, we can reconstruct BFB paths by connecting all the vertices on the BFB tree.

#### Resolving BFB path

##### Compose a BFB path with topological order

According to the definition, a BFB DAG is a superset of BFB trees, which represent many possible BFB paths. For simplicity, we follow topological orders to construct BFB trees and compose BFB paths. After finding a topological order by Supplementary Methods, Algorithm 2, we follow it to iteratively concatenate each pair of parent and child entities. During the process of constructing a BFB path, there is a temporary BFB path *P* that is extended by an entity in each iteration. According to the topological order, we insert one entity into a proper position in *P*, which still keeps a palindromic suffix after the insertion. Eventually, if all entities are added to *P*, we get the final BFB path (Supplementary Methods, Algorithm 3). As multiple BFB paths can be derived from a BFB DAG, Ambigram outputs the BFB path constructed in the first topological order derived by Supplementary Methods, Algorithm 2 by default. Note that entities on the BFB path are arranged in decreasing order of length. Moreover, Ambigram provides an option to output BFB paths composed in all topological orders for users to investigate.

##### Tolerating BFB path with imperfect FBIs

In a real scenario, an imperfect FBI junction may connect a segment to its neighbor’s reverse complementary segment, i.e., $({s}_{i},\overline{{s}_{j}})$, leading to the loss of segments. To figure out a BFB path with imperfect FBI junctions, we assume these junctions to be perfect, considering $({s}_{i},\overline{{s}_{j}})$ as $({s}_{i},\overline{{s}_{i}})$, when calculating entity CNs by ILP and building a BFB DAG. After finding a perfect BFB path, Ambigram will adjust the FBI junctions by revising ${s}_{i}| \overline{{s}_{i}}$ to ${s}_{i}| \overline{{s}_{j}}$.

##### Tolerating BFB path with DEL, DUP, and INS

In a real scenario, a region undergoing BFB can involve deletion (DEL), duplication (DUP), and insertion (INS), except for FBI and translocation. After constructing a BFB path of a complex BFB event, Ambigram revised local genome regions of different chromosomes on the BFB path based on intra-chromosomal SVs involved with deletion, duplication, and insertion.

##### Temporal orders of BFB cycle and translocation

In practice, a BFB event can involve translocation (TRX) on multiple chromosomes. Therefore, we propose two modes in accordance with two temporal orders of the BFB cycles and translocation, BFB-to-TRX and TRX-to-BFB. BFB-to-TRX assumes that translocation occurs after the BFB cycles. In this mode, we construct the BFB path from each chromosome separately and then iteratively concatenate two or more BFB paths with translocation junctions (Supplementary Methods, Algorithm 4). TRX-to-BFB supposes that the translocation happens before the BFB cycles. Here, we first integrate chromosomes with translocation and then take the synthesis as a virtual contig to reconstruct BFB paths.

##### BFB path with virus integration

Ambigram can resolve potential BFB paths mediated with oncovirus integration since human-virus integration is analog to translocation if we take both human chromosomes and virus genomes as contigs. There is no credible evidence that the BFB mechanism causes the rearrangement of the viral genome; therefore, we adopt the TRX-to-BFB mode to resolve the local genomic map of virus integration. Virus segments are inserted into a human chromosome, then several BFB cycles take place on the virus-integrated chromosome.

### Remarks of Ambigram

Suppose the number of segments is *n*, and the time and space complexity of Ambigram is *O*(*n*^3^) and *O*(*n*^2^), respectively (Supplementary Methods).

### Experiment settings

#### In silico processing

We designed six instances: one perfect BFB path on a single chromosome, one BFB path with imperfect FBI, two BFB paths integrated with translocation, one BFB path with intra-chromosome SVs, and one BFB path with virus integration. To simulate different sequencing methods in a BFB event, we first generated the FASTA file of the ground truth BFB path and reference path with GRCh38 reference genome. Then we used simulators, including wgsim^54^, PBSIM^55^, and LRSim^56^, to generate FASTQ files that include simulated reads. Moreover, we aligned the simulated reads with the reference genome to generate BAM files using the corresponding read alignment software, including BWA^57^, NGMLR^58^, and Long Ranger^59^. Furthermore, we extracted SV information as VCF files from the BAM files with different SV calling software, SvABA^60^, and Sniffles^58^. All simulation tools are listed in Supplementary Methods, Table S1. To evaluate the impact of tumor purity, we randomly mixed reference path and BFB path BAM files with a depth of 30× and tumor purities of 100%, 75%, 50%, 25%, 20%, and 10% for PE, 10×, ONT, and PB data, respectively. Furthermore, we sampled pure BFB path reads in BAM files at depths of 30×, 20×, 10×, and 5× to assess the impact of sequencing depth on deciphering BFB paths.

We also simulated 410 BFB paths for measuring the running time of Ambigram. Starting with a random reference path, we concatenated the path with its reverse complementary path and cut off a random number of tailing segments. Each BFB path was simulated iteratively in this way, given a segment count and FBI count. Besides, we added minor duplications onto a BFB path with a probability of 30%.

#### COLO829 processing

We downloaded the melanoma COLO829 cell line from PRJEB27698^13^ that provides diverse read information in BAM files derived from PE, PB, ONT, and 10x sequencing methods (with reference genome GRCh37-lite). To simulate varying tumor purity and sequencing depth, we used the same method of processing simulated data to merge tumor samples with normal samples and sample BAM files with various depths, respectively. Then we used the corresponding SV calling software (Supplementary Methods and Table S1) to extract SV information from these processed reads and select the SV sets containing FBIs involved with the two curated BFB instances. Also, we incorporated additional linkage information from linked reads (10×) and long reads (PB and ONT) to help Ambigram find more accurate and convincing BFB paths. Additionally, we ran AmpliconArchitect^19^ to get SV and CN information of COLO829 with default parameters. We further used AmpliconClassifier^19^ to detect BFB events in COLO829 by inputting the results from AmpliconArchitect.

#### CN and SV/FBI metrics for benchmarking

To evaluate the performance of a tool in reconstructing the BFB path, we measured the consistencies of segment CN and SV compared to the ground truth BFB path. First, we measured the segment CN consistency with the metric CN accuracy. The CN accuracy is defined as the ratio of segment CN correctly inferred; that is, the number of segments with correctly inferred CNs divided by the total number of segments. Second, we measured the SV/FBI consistency with SV/FBI precision, SV/FBI recall, and SV/FBI F1-score. Given a set of SVs/FBIs inferred by a tool and the set of ground truth SVs/FBIs from the BFB path, we counted the FP (false positive), TP (true positive), and FN (false negative). FP is the number of inferred SVs/FBIs that do not belong to ground truth; TP is the number of correctly inferred SVs/FBIs; and FN is the number of ground truth SVs/FBIs that are failed to infer. Then, we calculated precision, recall, and F1-score by $\,{{\mbox{Precision}}}=\frac{{{\mbox{TP}}}}{{{\mbox{TP}}}+{{\mbox{FP}}}},\,{{\mbox{Recall}}}=\frac{{{\mbox{TP}}}}{{{\mbox{TP}}}+{{\mbox{FN}}}\,}$, and $\,{{\mbox{F1-score}}}=2\times \frac{{{\mbox{Precision}}}\times {{\mbox{Recall}}}}{{{\mbox{Precision}}}+{{\mbox{Recall}}}\,}$.

#### HCC827, PD4875 and PD3641 processing

AmpliconReconstructor resolved BFB events on the HCC827 lung cancer cell line^20^. We obtained the SV and OM data from Luebeck et al.^20^. We wrote a script to convert the OM data into linkage information that Ambigram can utilize. For the breast cancer sample PD4875, we got the SV and CN information directly from Greenman et al.’s work^25^. As for the pancreatic cancer sample PD3641, the SV and CN information was obtained from Zakov et al.^23^. We fit the SVs and CNs into Ambigram with default parameters. For BFBFinder^24^ that only requires CNs, we ran it in the Poisson error model with parameters “-s -a -e=PoissonErrorModel -w=0.858” and chose the first string as its final output.

#### Single-cell processing

COLO829 single-cell data was downloaded from GSE151409^33^. The single-cell MKN45 data was downloaded from PRJNA498809^32^. The BAM and CN files for single cells were obtained from Cell Ranger (with the reference genome GRCh37)^32^. We grouped single cells into subclones based on hierarchical CN clustering. Then we independently sampled the BAM file with read information into different subclone BAM files and called subclone SVs with SvABA^60^. Next, we grouped SVs based on distance (Supplementary Methods, Algorithm 1) and manually selected the BFB candidate SV sets. For each BFB candidate, we utilized Ambigram to resolve BFB paths in relevant subclones in single-cell mode.

#### Oncovirus integration processing

We investigated nine integrations of HBV in three HCC samples^38^ and identified seven candidates for BFB. For each virus-induced BFB event, we directly used segment CNs, VITs, and SVs curated by Jia et al. (with reference genome GRCh37)^37^ to resolve BFB paths that can interpret the corresponding HBV interaction instance. As for the other HCC sample, S0007T1^39^, the SVs and CNs of the whole genome were called from SvABA^60^ and Patchwork^61^. We first grouped all SVs by distance (Supplementary Methods, Algorithm 1). Then we found two SV sets involved with virus-induced BFB. Finally, we utilized Ambigram to resolve the complex BFB events involved with virus integration under TRX-BFB mode.

HELA data and SIHA data were downloaded from SRP048769^40^; CRR046045 data were downloaded from NGDC with accession code CRX040585^45^. The reads were aligned to mixed reference (GRCh38 and HPV reference database PaVE, https://pave.niaid.nih.gov/explore/reference_genomes/human_genomes^62^) with BWA^57^. We called HPV integration sites and SVs using Manta^63^. For each sample, we searched for FBIs within local genome regions involved with HPV integrations and span several million base pairs. Then we took FBIs and HPV integrations in a local region as a BFB candidate SV set and used Ambigram to resolve the complex BFB events under TRX-BFB mode.

#### T2T processing

We downloaded the complete human telomere-to-telomere assembly (T2T-CHM13v1.1) from https://www.ncbi.nlm.nih.gov/assembly/GCA_009914755.3/^47^. We ran MUMmer (with parameter -l 5000)^64^ to align the whole genomic sequence with itself without considering matches on the same local regions. We found around 10,000 matched fragments ranging from 10 to 30 kilobase pairs (kbp). We considered the matched reverse complementary fragment pairs as indicators of BFB events, so we selected five regions with alternate forward and reverse-matched fragments, which resemble BFB paths. Then, we developed a dynamic programming algorithm (Supplementary Methods, Algorithm 5) to split each region into fragments of length 10 kbp and aligned the first fragment with the subsequent fragments in both forward and reverse directions. In addition, the algorithm evaluated the match rate with the number of matched base pairs divided by the average length of both matched fragments. In addition, we calculated the average match rate and count the number of matches in the five local regions. As a result, we selected two regions with a high match rate (97%) or many matched fragments (*n* = 23), and forward and reverse fragments in both regions were arranged in an alternate order. Furthermore, we considered the first fragment as a reference and split it into several segments based on breakpoints delimited by comparing the reference with other matched fragments (Fig. 5b). Finally, we counted the occurrence of each segment as CN and utilized Ambigram to resolve the BFB paths.

#### 1000GP and CHD processing

The 1000GP control data were obtained from http://ftp.sra.ebi.ac.uk/vol1/run/^48^. We downloaded the CHD band and relative data from dbGaP with accession codes phs001138.v3.p2 and phs001194.v2.p236^52^. We perform alignment using BWA^57^ and call SVs by SvABA^60^ and Manta^63^. If the 5’ and 3’ breakpoints of an SV can be detected separately in both SvABA and Manta with 100bp tolerance, we kept such consensus SVs with the breakpoints from SvABA. Then, we filtered consensus SVs with <20 supportive reads. We counted the occurrences of each FBI among three cohorts. On the one hand, we found BFB candidate SV sets for each 1000GP sample based on Algorithm 1 in Supplementary Methods. Then we selected genes truncated by recurrent FBIs in 1000GP data and investigated BFB candidate SV sets involved with these gene regions. On the other hand, we conducted a significant enrichment test with a chi-square test between the CHD proband and control cohorts for each FBI. We selected FBI truncated genes that occurred in at least 50% of probands. Moreover, we chose FBI truncated genes with a *p* value < 1e-5 in the proband enrichment test. As a result, we found nine genes and predicted their gene ontology and human phenotypes using Harmonizome (https://maayanlab.cloud/Harmonizome/)^65^. We found BFB candidate SV sets with a range <10 million base pairs (Mb) and applied Ambigram to decipher the local genomic map for probands.

### Reporting summary

Further information on research design is available in the Nature Portfolio Reporting Summary linked to this article.

### Supplementary information

Supplementary Information

Reporting Summary

## Data Availability

The COLO829 PE, 10x, PB, and ONT data are downloaded from PRJEB27698 [https://www.ebi.ac.uk/ena/browser/view/PRJEB27698]^13^. The HCC827 PE and OM data are retrieved from Luebeck et al.^20^. The PD4875 data is obtained from Greenman et al.^25^. The PD3641 data are obtained from Zakov et al.^23^. COLO829 single-cell data are downloaded from GSE151409 [https://0-www-ncbi-nlm-nih-gov.brum.beds.ac.uk/geo/query/acc.cgi?acc=GSE151409]^33^. mkn45 single-cell data are downloaded from PRJNA498809 [https://www.ncbi.nlm.nih.gov/bioproject/?term=PRJNA498809]^32^. The 101T, 260T, and 261T HCC data are obtained from Jia et al.^37^. The S0007T1 HCC data is obtained from the China National GeneBank DataBase (CNGBdb) with accession number CNP0003155 [https://db.cngb.org/search/project/CNP0003155/]^39^. HELA data and SIHA data are downloaded from SRP048769 [https://www.ncbi.nlm.nih.gov/Traces/study/?acc=SRP048769&o=acc_s%3Aa]^40^; CRR046045 data is downloaded from NGDC with accession code CRX040585 [https://ngdc.cncb.ac.cn/gsa/browse/CRA001401/CRX040585]^45^. The 1000GP data are obtained from http://ftp.sra.ebi.ac.uk/vol1/run/^48^. The CHD data are downloaded from dbGaP with accession codes phs001194.v3.p2 [https://www.ncbi.nlm.nih.gov/projects/gap/cgi-bin/study.cgi?study_id=phs001194.v3.p2]^52^. GRCh37 and GRCh38 reference genomes are downloaded from https://www.ncbi.nlm.nih.gov/datasets/genome/GCF_000001405.26/. The complete T2T genome is downloaded from https://www.ncbi.nlm.nih.gov/datasets/genome/GCA_009914755.3/. The processed data in this work are freely available at https://github.com/deepomicslab/Ambigram_paperor https://zenodo.org/badge/latestdoi/428970777^66^.

## Code availability

The C++ packages are available freely at https://github.com/deepomicslab/Ambigram or https://zenodo.org/badge/latestdoi/442341093^67^.

## References

[1] Robinson HM, Harrison CJ, Moorman AV, Chudoba I, Strefford JC (2007). Intrachromosomal amplification of chromosome 21 (iamp21) may arise from a breakage–fusion–bridge cycle. Genes, Chromosomes Cancer.

[2] Li Y (2014). Constitutional and somatic rearrangement of chromosome 21 in acute lymphoblastic leukaemia. Nature.

[3] Hicks J (2006). Novel patterns of genome rearrangement and their association with survival in breast cancer. Genome Res..

[4] Cheng C (2016). Whole-genome sequencing reveals diverse models of structural variations in esophageal squamous cell carcinoma. Am. J. Hum. Genet..

[5] Hillmer AM (2011). Comprehensive long-span paired-end-tag mapping reveals characteristic patterns of structural variations in epithelial cancer genomes. Genome Res..

[6] Xing R (2019). Whole-genome sequencing reveals novel tandem-duplication hotspots and a prognostic mutational signature in gastric cancer. Nat. Commun..

[7] Bignell GR (2007). Architectures of somatic genomic rearrangement in human cancer amplicons at sequence-level resolution. Genome Res..

[8] Kitada K, Yamasaki T (2008). The complicated copy number alterations in chromosome 7 of a lung cancer cell line is explained by a model based on repeated breakage-fusion-bridge cycles. Cancer Genetics Cytogenet..

[9] Lim G (2005). An integrated mband and submegabase resolution tiling set (smrt) cgh array analysis of focal amplification, microdeletions, and ladder structures consistent with breakage–fusion–bridge cycle events in osteosarcoma. Genes Chromosomes Cancer.

[10] Selvarajah S (2006). The breakage–fusion–bridge (bfb) cycle as a mechanism for generating genetic heterogeneity in osteosarcoma. Chromosoma.

[11] Selvarajah S (2008). Genomic signatures of chromosomal instability and osteosarcoma progression detected by high resolution array cgh and interphase fish. Cytogenet. Genome Res..

[12] Campbell PJ (2010). The patterns and dynamics of genomic instability in metastatic pancreatic cancer. Nature.

[13] Valle-Inclan JE (2022). A multi-platform reference for somatic structural variation detection. Cell Genomics.

[14] McClintock B (1939). The behavior in successive nuclear divisions of a chromosome broken at meiosis. Proc. Natl. Acad. Sci. USA.

[15] Yi K, Ju YS (2018). Patterns and mechanisms of structural variations in human cancer. Exp. Mol. Med..

[16] Li Y (2020). Patterns of somatic structural variation in human cancer genomes. Nature.

[17] DePinho RA, Polyak K (2004). Cancer chromosomes in crisis. Nat. Genet..

[18] Maciejowski J, de Lange T (2017). Telomeres in cancer: tumour suppression and genome instability. Nat. Rev. Mol. Cell Biol..

[19] Deshpande V (2019). Exploring the landscape of focal amplifications in cancer using ampliconarchitect. Nat. Commun..

[20] Luebeck J (2020). Ampliconreconstructor integrates ngs and optical mapping to resolve the complex structures of focal amplifications. Nat. Commun..

[21] Shale, C. et al. Unscrambling cancer genomes via integrated analysis of structural variation and copy number. *Cell Genomics* **2**, 100112 (2022). 10.1016/j.xgen.2022.100112 PMC9903802 36776527

[22] Kinsella M, Bafna V (2012). Combinatorics of the breakage-fusion-bridge mechanism. J. Comput. Biol..

[23] Zakov S, Kinsella M, Bafna V (2013). An algorithmic approach for breakage-fusion-bridge detection in tumor genomes. Proc. Natl. Acad. Sci..

[24] Zakov S, Bafna V (2015). Reconstructing breakage fusion bridge architectures using noisy copy numbers. J. Comput. Biol..

[25] Greenman C, Cooke S, Marshall J, Stratton M, Campbell P (2016). Modeling the evolution space of breakage fusion bridge cycles with a stochastic folding process. J. Math. Biol..

[26] Greenman CD, Penso-Dolfin L, Wu T (2020). The complexity of genome rearrangement combinatorics under the infinite sites model. J. Theor. Biol..

[27] Zheng GX (2016). Haplotyping germline and cancer genomes with high-throughput linked-read sequencing. Nat. Biotechnol..

[28] Zhang L, Zhou X, Weng Z, Sidow A (2019). Assessment of human diploid genome assembly with 10x linked-reads data. GigaScience.

[29] Pollard MO, Gurdasani D, Mentzer AJ, Porter T, Sandhu MS (2018). Long reads: their purpose and place. Hum. Mol. Genet..

[30] Amarasinghe SL (2020). Opportunities and challenges in long-read sequencing data analysis. Genome Biol..

[31] Pendleton M (2015). Assembly and diploid architecture of an individual human genome via single-molecule technologies. Nat. Methods.

[32] Andor N (2020). Joint single cell dna-seq and rna-seq of gastric cancer cell lines reveals rules of in vitro evolution. NAR Genom Bioinform..

[33] Velazquez-Villarreal EI (2020). Single-cell sequencing of genomic dna resolves sub-clonal heterogeneity in a melanoma cell line. Commun. Biol..

[34] Minussi DC (2021). Breast tumours maintain a reservoir of subclonal diversity during expansion. Nature.

[35] Chen L (2022). Somatic variant analysis suite: copy number variation clonal visualization online platform for large-scale single-cell genomics. Brief. Bioinform..

[36] Blumer A, Ehrenfeucht A, Haussler D, Warmuth MK (1987). Occam’s razor. Inform. Process. Lett..

[37] Jia W, Xu C, Li SC (2021). Resolving complex structures at oncovirus integration loci with conjugate graph. Brief. Bioinform..

[38] Sung W-K (2012). Genome-wide survey of recurrent hbv integration in hepatocellular carcinoma. Nat. Genet..

[39] Zou, H. et al. Hbv-integrated local genomic alterations reveal multicentric independent occurrences of multifocal hcc. *Clin. Transl. Med.* **13**, e1313 (2023). 10.1002/ctm2.1313 PMC10309081 37382888

[40] Hu Z (2015). Genome-wide profiling of hpv integration in cervical cancer identifies clustered genomic hot spots and a potential microhomology-mediated integration mechanism. Nat. Genet..

[41] Waters CE, Saldivar JC, Hosseini SA, Huebner K (2014). The fhit gene product: tumor suppressor and genome “caretaker”. Cell. Mol. Life Sci..

[42] Parrilla A (2020). Aurora borealis (bora), which promotes plk1 activation by aurora a, has an oncogenic role in ovarian cancer. Cancers.

[43] Weißbach S (2015). The molecular spectrum and clinical impact of dis 3 mutations in multiple myeloma. Br. J. Haematol..

[44] Lachmann M (2004). Pibf (progesterone induced blocking factor) is overexpressed in highly proliferating cells and associated with the centrosome. Int. J. Cancer.

[45] Cao C (2020). Hpv-ccdc106 integration alters local chromosome architecture and hijacks an enhancer by three-dimensional genome structure remodeling in cervical cancer. J. Genet. Genomics.

[46] Jen J, Wang Y-C (2016). Zinc finger proteins in cancer progression. J. Biomed. Sci..

[47] Nurk S (2022). The complete sequence of a human genome. Science.

[48] Byrska-Bishop, M. et al. High coverage whole genome sequencing of the expanded 1000 genomes project cohort including 602 trios. *BioRxiv* **185**, 3426-3440.e19 (2022). 10.1016/j.cell.2022.08.004 PMC9439720 36055201

[49] Soemedi R (2012). Phenotype-specific effect of chromosome 1q21. 1 rearrangements and gja5 duplications in 2436 congenital heart disease patients and 6760 controls. Hum. Mol. Genet..

[50] Miller DT (2010). Consensus statement: chromosomal microarray is a first-tier clinical diagnostic test for individuals with developmental disabilities or congenital anomalies. Am. J. Hum. Genet..

[51] Cooper GM (2011). A copy number variation morbidity map of developmental delay. Nat. Genet..

[52] Richter F (2020). Genomic analyses implicate noncoding de novo variants in congenital heart disease. Nat. Genet..

[53] Ho SS, Urban AE, Mills RE (2020). Structural variation in the sequencing era. Nat. Rev. Genet..

[54] Li, H. wgsim-read simulator for next generation sequencing. *Github Repository* (2011).

[55] Ono Y, Asai K, Hamada M (2013). Pbsim: Pacbio reads simulator–toward accurate genome assembly. Bioinformatics.

[56] Luo R, Sedlazeck FJ, Darby CA, Kelly SM, Schatz MC (2017). Lrsim: a linked-reads simulator generating insights for better genome partitioning. Comput. Struct. Biotechnol. J..

[57] Li, H. Aligning sequence reads, clone sequences and assembly contigs with bwa-mem. *arXiv* https://arxiv.org/abs/1303.3997 (2013).

[58] Sedlazeck FJ (2018). Accurate detection of complex structural variations using single-molecule sequencing. Nat. Methods.

[59] Marks P (2019). Resolving the full spectrum of human genome variation using linked-reads. Genome Res..

[60] Wala JA (2018). Svaba: genome-wide detection of structural variants and indels by local assembly. Genome Res..

[61] Mayrhofer M, DiLorenzo S, Isaksson A (2013). Patchwork: allele-specific copy number analysis of whole-genome sequenced tumor tissue. Genome Biol..

[62] Van Doorslaer K (2012). The papillomavirus episteme: a central resource for papillomavirus sequence data and analysis. Nucleic Acids Res..

[63] Chen X (2016). Manta: rapid detection of structural variants and indels for germline and cancer sequencing applications. Bioinformatics.

[64] Marçais G (2018). Mummer4: a fast and versatile genome alignment system. PLoS Comput. Biol..

[65] Rouillard, A. D. et al. The harmonizome: a collection of processed datasets gathered to serve and mine knowledge about genes and proteins. *Database* **2016**, baw100 (2016). 10.1093/database/baw100 PMC4930834 27374120

[66] Li, C., Chen, L., Pan, G., Zhang, W. & Li, S. C. deepomicslab/ambigram_paper: v1.0.0 (2023). 10.5281/zenodo.8202100.

[67] Li, C., Chen, L., Pan, G., Zhang, W. & Li, S. C. deepomicslab/ambigram: v1.0.0 (2023). 10.5281/zenodo.8202067.

[68] Jia, W., Li, H., Li, S., Chen, L. & Li, S. C. Oviz-bio: a web-based platform for interactive cancer genomics data visualization. *Nucleic Acids Res.* (2020). 10.1093/nar/gkaa371 PMC7319551 32392343

