# Supplementary material for: Deciphering complex breakage-fusion-bridge genome rearrangements with Ambigram
Source: Nat Commun. 2023 Sep 8;14:5528. doi: 10.1038/s41467-023-41259-w (PMC10491683; doi:10.1038/s41467-023-41259-w)
Supplement: Supplementary file 1 — Supplementary Information [file 41467_2023_41259_MOESM1_ESM.pdf]

Deciphering complex breakage-fusion-bridge genome rearrangements with Ambigram
-
Supplementary Information

Chaohui Li[†], Lingxi Chen[†], Guangze Pan, Wenqian Zhang, Shuai Cheng Li[*]

Contents

1 Supplementary Methods **3**
1.1 Finding BFB candidate SV sets . . . 3
1.2 Formalating BFB as a DAG . . . 3
1.3 BFB path and DAG . . . 4
1.4 Integer linear programming . . . 4
1.5 Single-cell mode . . . 5
1.6 Extra information from linked reads, long reads, and optical mapping alignment . . . 5
1.7 Compose a BFB path by connecting entities in the topological order . . . 5
1.8 Algorithm of the BFB-TRX mode . . . 6
1.9 Algorithm of T2T alignment . . . 6
1.10 Remarks of Ambigram . . . 7
1.10.1 Searching space . . . 7
1.10.2 Time complexity . . . 7
1.10.3 Space complexity . . . 7

2 Supplementary Tables **7**

3 Supplementary Figures **11**

List of Supplementary Tables

1 Genes overlapping with recurrent FBIs. We used the one-sided Chi-square test without adjustments. 8
2 Gene Ontology (GO) and human phenotypes (hP) of CHD genes inferred by Harmonizome [1]. "-" means not applicable. . . . 9
3 Summary of tools used in experiments. "-" means not applicable. . . . 10

List of Supplementary Figures

1 Simulated instance 1 - complex BFB involving chr7. . . . 11
2 Simulated instance 2 - complex BFB involving chr3. . . . 12
3 Simulated instance 3 - complex BFB involving inter-chromosomal rearrangements on chr2, chr6, and chr13. . . . 13
4 Simulated instance 4 - complex BFB involving inter-chromosomal rearrangements on chr2 and chr6. . . . 14
5 Simulated instance 5 - complex BFB involving inter-chromosomal rearrangements on chr6. . . . 15
6 Simulated instance 6 - virus-induced complex BFB involving inter-chromosomal rearrangements on chr8 and virus. . . . 16
7 The SV precision and SV F1-score of Ambigram for six simulated BFB instances. . . . 17
8 The SV and CN statistics of six simulated BFB instances. . . . 18
9 Benchmarking BFBFinder with *in silico* data . . . 19
10 The CN accuracy, SV precision, SV recall, and SV F1-score of Ambigram and BFBFinder for 410 simulated BFB paths. . . . 20
11 COLO829 instance 1 - the explicit evolution process of complex BFB in the first stage. . . . 21

12 COLO829 instance 1 - complex BFB solved by LINX. 22
13 COLO829 detected in AmpliconArchitect and AmpliconClassifier. 23
14 Results of COLO829 instances from BFBFinder. 24
15 COLO829 instance 2 - complex BFB involving inter-chromosomal rearrangements on chr15, chr6, and chr20. 25
16 The FBI precision and FBI F1-score of Ambigram for two BFB instances on COLO829. 26
17 The SV and CN statistics of two BFB instances on COLO829. 27
18 The BFB event in a lung cancer sample HCC827 [2]. 28
19 BFB events in breast cancer sample PD4875 [3] and pancreatic cancer sample PD3641 [4]. . . . 29
20 (a-b) The copy number profiles of single cells from cell lines COLO829 and mkn45. 30
21 Single-cell COLO829 instance 2 - complex BFB involving inter-chromosomal rearrangements on chr15, chr6, and chr20 with heterogeneity among subclones. 31
22 Complex BFB event on chr1 for mkn45. 32
23 Complex BFB event on chr3 for mkn45. 33
24 Complex BFB event on chr11 in subclone M8 for mkn45. 34
25 Complex BFB event on chr11 in subclones A and M6 for mkn45. 35
26 Complex BFB event on chr12 in subclone M8 for mkn45. 36
27 Complex BFB event on chr12 in subclones M6 and M7 for mkn45. 37
28 Complex BFB event on chr15 for mkn45. 38
29 Complex BFB involving HBV integration and genes *CGN*, *TUFT1*, *SNORA44*, and *MIR554* on chr1 of HCC 101T [5, 6]. 39
30 Complex BFB involving HBV integration and genes *RCC2* and *ARHGEF10L* on chr1 of HCC 260T [5, 6]. 40
31 Complex BFB involving HBV integration and gene *ANK3* on chr10 of HCC 260T [5, 6]. 41
32 Complex BFB involving HBV integration and genes *TERT* and *MIR4457* on chr5 of HCC 261T [5, 6]. 42
33 Complex BFB involving HBV integration and gene *BBS2* on chr16 of HCC 261T [5, 6]. 43
34 Complex BFB involving HPV integration and gene *FHIT* on chr3 of HELA cell line. 44
35 Complex BFB involving HPV integration on chr13 of SIHA cell line. 45
36 Complex BFB involving HPV integration on chr19 of cervical cancer sample CRR046045. 46
37 BFB analysis in 923 healthy genomes from 1000GP. 47
38 Recurrent complex BFB involving chr21 and chr19 of CHD sample SRR3949329. 48
39 Recurrent complex BFB involving chr1 of CHD sample SRR3945479. 49
40 Recurrent complex BFB involving chr12 and chr9 of CHD sample SRR3989901. 50
41 Recurrent complex BFB involving chr7 of CHD sample SRR5114912. 51
42 Recurrent complex BFB involving chr7 of CHD sample SRR5114981. 52
43 Running time evaluation. 53
44 Illustration of BFB paths. 54
45 One example of perfect BFB. 55
46 The illustration of SV junction type. 56

1 Supplementary Methods

1.1 Finding BFB candidate SV sets

This is a clustering method to identify the sets of SVs that may be involved in the same CSV event like BFB. Given a large data set consisting of miscellaneous SVs, we define each SV as a pair of chromosome names and breakpoints, denoted $(chr_1, bkp_1, chr_2, bkp_2)$, where $bkp_1, bkp_2 \in N$ and chr_1 and chr_2 are the chromosomes that the two breakpoints belong to, respectively. The distance of two SVs is defined by the least absolute value among four pairs of breakpoints' differences, and each SV contributes one breakpoint to every pair. Note that the difference is taken as infinity if two breakpoints belong to different chromosomes. Generally, we use the breadth-first search algorithm to cluster SVs into groups based on the SV distance that should be less than a predefined distance α. Besides, the range of an SV group is defined as the difference between the largest breakpoint and smallest breakpoint among all SVs in the group. We also set a range limit β so that each resultant SV set has a range less than the limit.

Algorithm 1 Cluster SVs into SV sets.

1: Sort all SVs in ascending order of breakpoints;
2: Let Q be a queue and put an ungrouped SV into Q;
3: Let S be an empty SV set;
4: **while** Q is not empty **do**
5:   Fetch the first SV u from Q, and add u into S;
6:   **for** each ungrouped SV v **do**
7:     **if** distance of u and $v \leq \alpha$ **then**
8:       **if** range of S including $v \leq \beta$ **then**
9:         Add v into S;
10:       **end if**
11:     **end if**
12:   **end for**
13: **end while**
14: Get a SV set S;
15: **if** some SVs are ungrouped **then**
16:   Go to line 2;
17: **end if**

1.2 Formalating BFB as a DAG

Based on the definitions of mono-chains, loops, and BFB paths in Methods, we can conclude the following properties:

Lemma 1.1 *Removing a loop from a BFB path will produce another BFB path.*

We denote a BFB path with a loop as $P = e(a_1, b_1)|e(a_2, b_2)|...|e(a_n, b_n)$, where $e(a_k, b_k)$ is a loop. Since a loop is a symmetric entity, the entities flanking a loop are either reverse complements or a pair of parent and child entities. Hence, the entities $e(a_{k-1}, b_{k-1})$ and $e(a_{k+1}, b_{k+1})$ share a pair of reverse complementary segments linked by an FBI junction. After removing the loop, the path becomes $P' = e(a_1, b_1)|...|e(a_{k-1}, b_{k-1})|e(a_{k+1}, b_{k+1})|...|e(a_n, b_n)$, which still keeps the continuity and palindromic suffix. Therefore, the resultant path P' is still a BFB path.

Lemma 1.2 *Any consecutive mono-chains on a non-loop BFB path are a pair of parent and child mono-chains.*

We have a non-loop BFB path $P = m(a_1, b_1)|m(a_2, b_2)|...|m(a_n, b_n)$. For any consecutive mono-chains $m(a_k, b_k)$ and $m(a_{k+1}, b_{k+1})$, where $k \in \{1, 2, ..., n-1\}$, they share a pair of reverse complementary segments linked by an FBI junction because of the continuity of a BFB path. Besides, the length of $m(a_k, b_k)$ is larger than the length of $m(a_{k+1}, b_{k+1})$. Otherwise, there is a loop if the mono-chains have equal length or a structure that compromise the palindromic property of a BFB path if the length of $m(a_k, b_k)$ is less than that of $m(a_{k+1}, b_{k+1})$. Therefore, the consecutive mono-chains on P are a pair of parent and child mono-chains. In other words, a non-loop BFB path is composed of length-decreasing and consecutive mono-chains.

Theorem 1.3 *Any BFB path can be constructed by connecting mono-chains and then inserting loops.*

According to *Lemma 1.1*, we can iteratively remove loops from a BFB path, and finally, we will get a non-loop BFB path that only consists of mono-chains if there exists a mono-chain on the original path. Hence, any BFB

path can be represented by mono-chains and loops. Based on *Lemma 1.2*, we can construct a non-loop BFB path by connecting all pairs of parent and child mono-chains. Then we insert loops into appropriate positions on the non-loop BFB path so that the resultant path is still a BFB path. Therefore, any BFB path can be produced by integrating mono-chains and then inserting loops into some positions.

Lemma 1.4 *A BFB path can be equivalently represented by a BFB tree.*

Given a BFB path P, we can build a binary tree, termed *BFB tree*, where vertices represent entities and edges direct parent entities to child entities. According to *Theorem 1.3*, we first connect all pairs of parent and child mono-chain vertices. Each child mono-chain is a left child vertex of its parent mono-chain. Then we follow the order of entities on P to link loop vertices by directed edges from their parent entity vertices. Each loop is a left child vertex of its parent entity. As a result, we get a BFB tree, which equivalently represents P by order of entities derived by preorder traversal (e.g., Supplementary Fig. 44).

Theorem 1.5 *A BFB DAG can derive multiple BFB paths.*

Given sequencing data, we can gain the parent-child relationships between entities. Hence, we can establish a BFB directed acyclic graph (DAG) based on the steps shown in Methods. Note that sequencing data cannot determine the exact position for each copy of a loop with multiple occurrences. Therefore, we map CNs to the theoretical model by collapsing all copies of the loop into one vertex with CN larger than 1. According to *Lemma 1.4*, a BFB tree is built by following the order of entities on a BFB path to link parent and child entities. Since a BFB DAG is constructed by connecting all pairs of parent and child entities, a BFB tree is a sub-tree in a BFB DAG. Besides, a BFB tree is equivalent to a BFB path, so a BFB path is one solution derived from the BFB DAG. As a result, we can extract multiple BFB trees from a BFB DAG to derive several BFB paths, respectively.

1.3 BFB path and DAG

According to *Theorem 1.5*, there is an important mapping relationship between BFB paths and DAG, i.e., any BFB path can be collapsed into a DAG, while a DAG can represent one or more BFB paths. To derive BFB paths from a BFB DAG, we can follow different traversal orders to construct multiple BFB trees, each of which corresponds to a BFB path. To reduce our search space, we follow topological orders to reconstruct BFB paths (Algorithm 3). However, since the sequencing data cannot determine the exact position for each copy of a loop, the loop with multiple copies repeats in one position by default. With long reads or linked reads, we can distribute the copies to appropriate positions and reconstruct a BFB path that better fits the given information.

1.4 Integer linear programming

Apart from the ILP objective function and two ILP constraints that define CN differences, we incorporate additional domain knowledge to refine the ILP results and meet some special requirements. There are several constraints that guarantee the connectivity of the output entities so that all of them can be integrated into a BFB DAG for constructing BFB paths.

Firstly, we denote a set of segments involved in a BFB event as $S = \langle s_1, s_2, ..., s_n \rangle$. For any mono-chain $m(i,j)$, we define the set of its child mono-chains as $P_m^{i,j} = \{m(a,b) \big| a > i, b = j \text{ } or \text{ } a = i, b < j\}$. According to *Lemma 1.2*, any non-loop BFB path consists of length-decreasing mono-chains with the parent-child relationship. Therefore, each mono-chain has at most one child mono-chain. Hence, we have the following ILP constraint to guarantee that no mono-chain has more than one child mono-chains.

$$\left[\sum_{m(a,b) \in P_m^{i,j}} c_m(a,b) \right] + c_m(i,j) \leq 2, \; \forall i,j \in \{1,2,...,n\} \tag{1}$$

Besides, for any mono-chain $m(i,j)$, we define the set of its parent mono-chains as $P_m^{i,j} = \{m(a,b) \big| a < i, b = j \text{ } or \text{ } a = i, b > j\}$. According to *Lemma 1.2*, we have the following inequality that guarantees the mono-chain (except the reference path) has at least one parent mono-chain. As a result, the child mono-chain has at least a predecessor to follow and becomes a valid vertex in a BFB DAG.

$$\left[\sum_{m(a,b) \in P_m^{i,j}} c_m(a,b) \right] - c_m(i,j) \geq 0, \; \forall i,j \in \{1,2,...,n\} \tag{2}$$

Similarly, for any loop $l(i,j)$, we denote the set of parent entities as $P_e^{i,j} = \{e(a,b) \big| a < i, b = j \; or \; a = i, b > j\}$ since a loop can follow either a parent mono-chain or a parent loop in a BFB path. Hence, we have another formula that guarantees every loop has at least one parent entity. Therefore, the child loop can be a successor of a parent mono-chain or inserted into a parent loop, composing a BFB DAG.

$$\left[\sum_{e(a,b) \in P_e^{i,j}} c_e(a,b) \right] - c_l(i,j) \geq 0, \;\; \forall i,j \in \{1, 2, ..., n\} \tag{3}$$

1.5 Single-cell mode

We also provide a method to reconstruct BFB paths from single-cell data. Users can input CN profiles and SV information of multiple subclones evolving over time. Ambigram will add ILP constraints on common mono-chains and loops, which are shared by all the subclones. For any pair of subclones, we define $c_m(a,b)$ and $c'_m(a,b)$ as the CNs of $m(a,b)$ in the two subclones. Similarly, we denote the CNs of $l(a,b)$ in both subclones by $c_l(a,b)$ and $c'_l(a,b)$. Moreover, we have the sets of all mono-chains and loops $M = \{m(a,b) \big| a, b \in \{1, 2, ..., n\}\}$ and $L = \{l(a,b) \big| a, b \in \{1, 2, ..., n\}\}$, where $a \leq b$. Notably, there are $\binom{n}{2} = \frac{n(n-1)}{2}$ mono-chains and loops, respectively. Furthermore, our algorithm will add the errors into the objective function (Formula 1 in Methods), which considers the CN differences of mono-chains and loops shared by subclones, denoted by δ_m and δ_l, respectively. As a result, we can reconstruct several BFB paths with some similar parts. Here are the objective function (Formula 4) and ILP constraints (Formula 5 and 6) and for the Single-cell mode:

$$\min \sum_{i=1}^{n} \varepsilon_i + \sum_{i=1}^{n} \xi_{i,i} + \sum_{m,l=1}^{\frac{n(n-1)}{2}} (\delta_p + \delta_l) \tag{4}$$

$$-\varepsilon_p \leq c_m(a,b) - c'_m(a,b) \leq \varepsilon_p, \;\; \forall m(a,b) \in M \tag{5}$$

$$-\varepsilon_l \leq c_l(a,b) - c'_l(a,b) \leq \varepsilon_l, \;\; \forall l(a,b) \in L \tag{6}$$

1.6 Extra information from linked reads, long reads, and optical mapping alignment

Since linked reads, long reads, and optical mapping alignment data provide extra information, our algorithm can use the linkage information from them, which indicates possible connections among segments, to construct more convincing BFB paths in terms of the observed CN and SV information. We represent the extracted linkage information by a set of entities $E = \{e(i,j) \big| i,j \in \{1, 2, ..., n\}\}$, where $s_i s_{i+1} ... s_j$ is a linked genome sequence indicated by linkage information. Then we constrain the CN of entities in E in the ILP Formula 7 to improve the probability that these entities appear in the output as parts of BFB paths. As a result, the linkage information can help our algorithm construct BFB paths that better fit the real scenario.

$$\sum_{e(a,b) \in E} c_e(a,b) > 0 \tag{7}$$

1.7 Compose a BFB path by connecting entities in the topological order

We use recursion and backtracking to find all topological orders from a BFB DAG (Supplementary Methods, Algorithm 2). Then we follow the first topological order in the result to construct a BFB path (Supplementary Fig. 45). There is a temporary BFB path P that will be extended by an entity in each iteration. Suppose we get m entities in a topological order $E = [e(a_1,b_1), e(a_2,b_2), ..., e(a_m,b_m)]$, we fetch the first entity from E and initialize the temporary BFB path as $P = e(a_1,b_1)$. Then we iteratively fetch an entity from E and add it into an appropriate position following its parent in P. For each entity, we start searching for the position from the tail of the path P. During the process, the neighboring entities are a pair of parent and child, and P always keeps a palindromic suffix. Eventually, if all entities in E are added into P, the final BFB path consisting of E is derived.

Algorithm 2 Find all topological orders.

1: Initialize all entities in the BFB DAG as unvisited;
2: Initialize an emtpy ordered list of entiteis E;
3: Add an entity e with indegree = 0 into E, and set it as visited;
4: Decrease indegree of child entities of e by 1;
5: Recursively run line 3 until all entities have 0 indegrees, and mark E as a topological order;
6: Remove e from E, reset it as unvisited,
7: Iecrease indegree of child entities of e by 1, and go to line 3;
8: Return all topological orders;

Algorithm 3 Compose a BFB path in the topological order.

Sort all entities in E by a topological order;
Fetch the first entity in E and initialize P as the entity;
for each entity $e \in E$ **do**
    Find an appropriate position for inserting e in P
    Add e into the position in P;
    Remove e from E;
end for
Return P;

1.8 Algorithm of the BFB-TRX mode

After constructing BFB paths in local genome regions, we design an algorithm to concatenate two or more BFB paths with translocation. Given the segment set $S = \langle s_1, s_2, ..., s_n \rangle$, we define translocation by a pair of segments (s_i, s_j), where $i, j \in \{1, 2, ..., n\}$. Note that s_i can be either segment s_i or reverse complementary segment $\overline{s_i}$. Then we group m translocation in a set $T = \big\{(s_i, s_j)_k | \forall k \in \{1, 2, ..., m\},\ i, j \in \{1, 2, ..., n\}\big\}$. Moreover, we sort all translocation in T so that $s_j \in (s_i, s_j)_a$ and $s_i \in (s_i, s_j)_{a+1}$ belong to the same BFB path, for $\forall a \in \{1, 2, ..., m-1\}$. As a result, we iteratively concatenate BFB paths by translocation in T.

Algorithm 4 Concatenate BFB paths with translocation.

Fetch the first translocation $(s_i, s_j)_1$ in T;
Find BFB paths P_1 and P_2 that are connected by $(s_i, s_j)_1$;
Cut P_1 at the last position of s_i and P_2 at the first position of s_j;
Link truncated P_1 and P_2 as P;
for each translocation $(s_i, s_j)_k \in T$ **do**
    Set $P_1 = P$ and find P_2 that s_j belongs to.
    Cut P_1 at the last position of s_i and P_2 at the first position of s_j;
    Link truncated P_1 and P_2 as P;
end for
Return P;

1.9 Algorithm of T2T alignment

We design an algorithm to align partial sequences on the T2T complete human genome efficiently. This algorithm is based on dynamic programming and supports both forward and reverse alignment. Given two genomic sequences G_1 and G_2 that consist of four letters "A", "T", "C", and "G" representing four bases, the algorithm can find the longest common subsequence (LCS) present in both of them. The LCS of G_1 and G_2 appear in the same relative order but are not necessarily continuous. As for reverse alignment, two genomic sequences are reverse complements if their bases hold a complementary mapping relationship, which is "A" corresponds to "T" and "C" corresponds to "G". To check if two sequences are reversely aligned, we try to find the longest palindrome subsequence (LPS) of both sequences. The LPS is a subsequence that appears as a reverse complement in reverse relative order in two sequences but is not necessarily continuous. To find the LPS of two input sequences G_1 and G_2, we first convert G_2 into its reverse complement and then use the same method for finding LCS to get the LPS of both sequences. For both LCS and LPS, We evaluate the similarity between two input sequences by the length of LCS or LPS divided by the average length of both input sequences. If the similarity is higher than 0.8, we consider the two sequences are valid matches.

Algorithm 5 Align two genome sequences.

Get lengths of G_1 and G_2 as m and n, respectively;
Initialize a $(m+1) \times (n+1)$ array A with the first row and the first column equal to 0;
for i in $[1, 2, ..., m]$ **do**
  for j in $[1, 2, ..., n]$ **do**
    if i^{th} letter of G_1 is equal to j^{th} letter of G_2 **then**
      $A_{i,j} \leftarrow A_{i-1,j-1} + 1$;
    else
      $A_{i,j} \leftarrow max(A_{i,j-1}, A_{i-1,j})$;
    end if
  end for
end for
Initialize an empty sequence S;
while $m > 0$ and $n > 0$ **do**
  if $A_{m,n} = A_{m,n-1}$ **then**
    $n \leftarrow n - 1$;
  else if $A_{m,n} = A_{m-1,n}$ **then**
    $m \leftarrow m - 1$;
  else
    Put m^{th} letter of G_1 into the first position of S;
    $n \leftarrow n - 1$, $m \leftarrow m - 1$;
  end if
end while
Return S;

1.10 Remarks of Ambigram

To elaborate on this part better, we define the number of segments as n and the total number of mono-chains and loops as m. According to the definition of mono-chains and loops, each of them is composed of two segments. Therefore, the numbers of mono-chain candidates and loop candidates are equal. Then the total number is $m = 2 \times \frac{n(n+1)}{2} = n(n+1)$.

1.10.1 Searching space

The searching space is proportional to the total number of possible BFB paths. Based on the algorithms explained above, the permutations of loops that are inserted into a non-loop BFB path determine the searching space. In the worst case, all possible mono-chains are used to construct a non-loop BFB path, i.e., $s_1s_2...s_n|\overline{s_ns_{n-1}...s_2}|s_2s_3...s_{n-1}|...|s_{n/2}$. Given $\frac{n(n+1)}{2}$ loops, each loop has 2 positions for insertion, so we have $O(n^2)$ possible BFB paths. Therefore, the search space is $O(n^2)$.

1.10.2 Time complexity

There are several parts that cost the most running time: (1) Solve the copy numbers of entities (mono-chains and loops) with ILP that includes constraints between entities - $O(n^3) = O(m) \times O(n)$; (2) Construct BFB DAG by connecting pairs of entities and all topological orders - $O(n^3)$; (3) Find all valid topological orders - $O(n^3) = O(searching\ space * length\ of\ a\ BFB\ path)$; (4) Concatenate BFB paths with translocation junctions - $O(n^3)$. In conclusion, the overall time complexity is $O(n^3)$.

1.10.3 Space complexity

Most space is used to record entities and all topological orders. According to the search space, the upper bound of the number of topological orders is $O(n^2) = O(m)$. As a result, the space complexity is $O(m)$.

2 Supplementary Tables

Gene	Region (hg38)	Proband Count	Relative Count	1000GP Count	P-Value
APP	chr21:25880550-26171128	311 (94%)	569 (93%)	844 (91%)	1.3174e-1
C4BPA	chr1:207104233-207144972	195 (59%)	382 (62%)	484 (52%)	4.3633e-2
BORCS5	chr12:12357078-12471233	172 (52%)	313 (51%)	385 (42%)	1.3669e-3
PTPRQ	chr12:80402178-80680273	103 (31%)	165 (27%)	74 (8%)	7.7274e-25
PUS7	chr7:105439661-105522271	95 (29%)	160 (26%)	146 (16%)	4.4351e-7
ITPRID1	chr7:31514090-31658720	94 (28%)	139 (23%)	152 (16%)	3.5524e-6
PLEKHB2	chr2:131104847-131353709	54 (16%)	93 (15%)	64 (7%)	8.4805e-7
IL1RAPL1	chrX:28587446-29956718	51 (15%)	99 (16%)	54 (6%)	1.2342e-7
EXT1	chr8:117794490-118111826	48 (15%)	75 (12%)	57 (6%)	4.3498e-6

Supplementary Table 1: Genes overlapping with recurrent FBIs. We used the one-sided Chi-square test without adjustments.

Gene	GO/HP Code	Description
APP	GO:0086012	Membrane depolarization during cardiac muscle cell action potential
	GO:0086064	Cell communication by electrical coupling involved in cardiac conduction
	GO:0086065	Cell communication involved in cardiac conduction
	HP:0001634	Mitral valve prolapse
	HP:0001653	Mitral regurgitation
	HP:0001663	Ventricular fibrillation
	HP:0002521	Hypsarrhythmia
	HP:0011663	Right ventricular cardiomyopathy
C4BPA	HP:0001718	Mitral stenosis
	HP:0001658	Myocardial infarction
	HP:0001678	Atrioventricular block
BORCS5	-	-
PTPRQ	GO:0055003	Cardiac myofibril assembly
	GO:0035909	Aorta morphogenesis
	GO:0021591	Ventricular system development
	GO:0060039	Pericardium development
	GO:0007512	Adult heart development
	HP:0011663	Right ventricular cardiomyopathy
	HP:0004756	Ventricular tachycardia
	HP:0001659	Aortic regurgitation
	HP:0001644	Dilated cardiomyopathy
	HP:0001718	Mitral stenosis
	HP:0005110	Atrial fibrillation
	HP:0001660	Truncus arteriosus
PUS7	-	-
ITPRID1	-	-
PLEKHB2	GO:0086012	Membrane depolarization during cardiac muscle cell action potential
	GO:0060371	Regulation of atrial cardiac muscle cell membrane depolarization
	HP:0001658	Myocardial infarction
	HP:0001663	Ventricular fibrillation
	HP:0001657	Prolonged QT interval
	HP:0003115	Abnormal EKG
	HP:0001640	Cardiomegaly
IL1RAPL1	GO:2000727	Positive regulation of cardiac muscle cell differentiation
	GO:0086012	Membrane depolarization during cardiac muscle cell action potential
	HP:0002521	Hypsarrhythmia
EXT1	GO:0060973	Cell migration involved in heart development
	GO:0003203	Endocardial cushion morphogenesis
	GO:0060039	Pericardium development
	GO:2000826	Regulation of heart morphogenesis
	GO:0003188	Heart valve formation
	GO:0003179	Heart valve morphogenesis
	GO:0060317	Cardiac epithelial to mesenchymal transition
	GO:0061311	Cell surface receptor signaling pathway involved in heart development
	HP:0001634	Mitral valve prolapse
	HP:0005111	Dilatation of the ascending aorta
	HP:0001718	Mitral stenosis
	HP:0011663	Right ventricular cardiomyopathy
	HP:0001653	Mitral regurgitation
	HP:0001658	Myocardial infarction

Supplementary Table 2: Gene Ontology (GO) and human phenotypes (hP) of CHD genes inferred by Harmonizome [1]. “-” means not applicable.

Experiment	Protocol	Simulator	Alignment	Reference	SV Calling
In silico	PE	wgsim[7]	BWA-MEM[8]	GRCh38	SvABA[9]
In silico	PB	PBSIM[10]	NGMLR[11]	GRCh38	Sniffles[11]
In silico	ONT	PBSIM[10]	NGMLR[11]	GRCh38	Sniffles[11]
In silico	10x	LRSim[12]	Long Ranger[13]	GRCh38	SvABA[9]
COLO829	PE	-	-	GRCh37-lite	SvABA[9]
COLO829	PB	-	-	GRCh37-lite	Sniffles[11]
COLO829	ONT	-	-	GRCh37-lite	Sniffles[11]
COLO829	10x	-	-	GRCh37-lite	SvABA[9]
COLO829	SC	-	-	GRCh37	SvABA[9]
HBV	PE	-	-	GRCh37, AB644284.1, and DQ089801.1	Jia *et al.* [6]
HPV	PE	-	BWA-MEM[8]	GRCh38 and PaVE	Manta [14]
S0007T1	PE	-	-	AB644284.1	SvABA [9], Patchwork [15]
MKN45	SC	-	-	GRCh37	SvABA[9]
CHD	PE	-	BWA-MEM[8]	GRCh38	SvABA[9], Manta [14]
T2T	-	-	MUMmer [16] Algorithm 4	T2T	-

Supplementary Table 3: Summary of tools used in experiments. “-” means not applicable.

3 Supplementary Figures

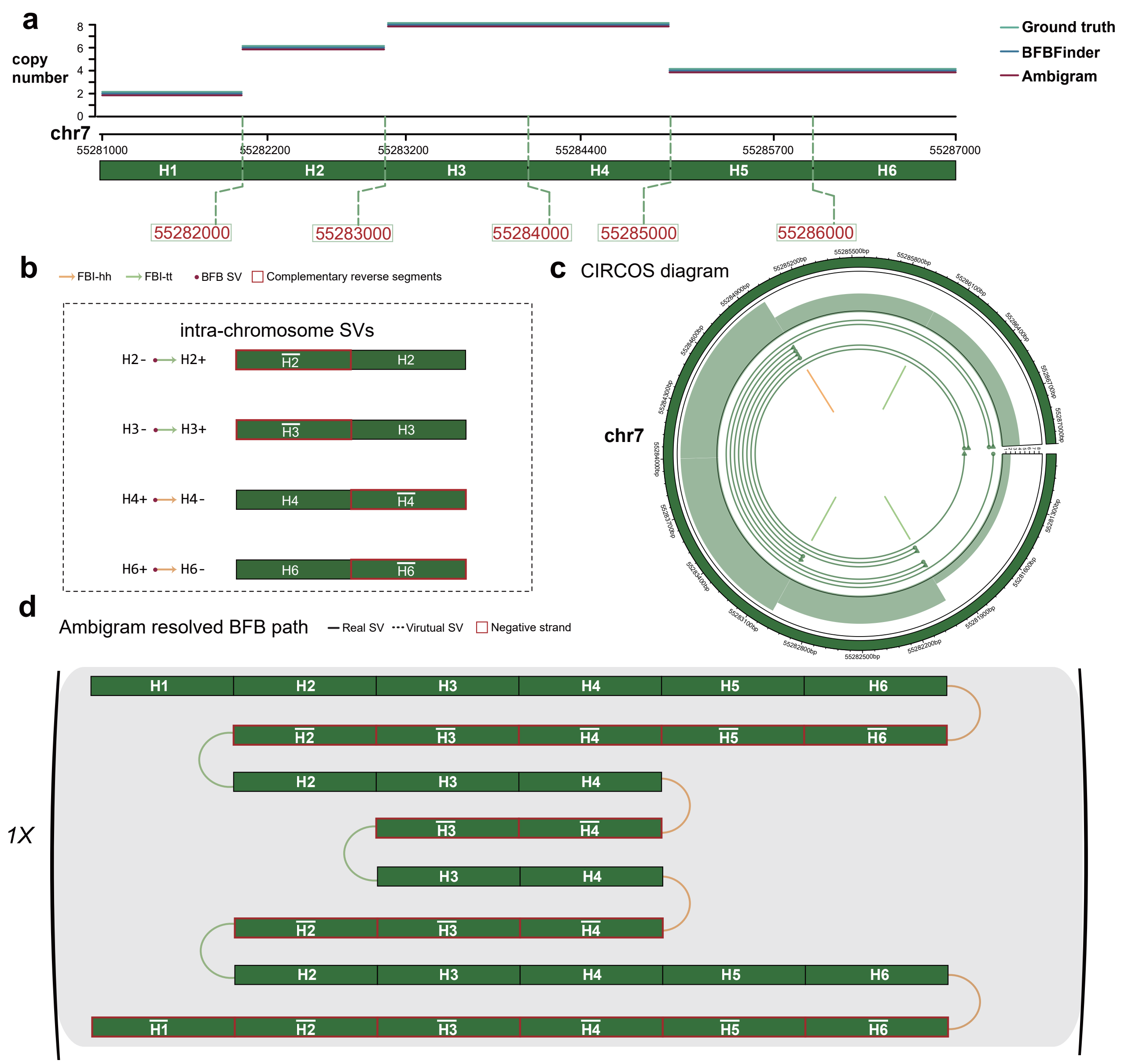

Supplementary Figure 1: Simulated instance 1 - complex BFB involving chr7.
(a) The FBI breakpoints split the local genome region of chr7 into six segments. The stairstep plot shows the ground truth CNs of segments and the CNs derived by BFBFinder and Ambigram. (b) List of FBIs and the segments connected by them. The head-to-head (hh) and tail-to-tail (tt) FBIs are colored light yellow and light green, respectively. The reverse complementary segments have a red border. (c) CIRCOS diagram of the BFB event. The outermost track shows the local genome regions involved with the BFB event. The second outermost track illustrates the input region CNs, and the third track indicates the resolved CNs by Ambigram. Besides, the third track shows the resolved BFB paths, in which the circle and triangle points refer to the 5' end and 3' end, respectively. The innermost part represents all the SVs involved with the BFB event. (d) Ambigram resolved the BFB path. Overall, chr7 undergoes four BFB cycles. The first BFB cycle occurs when segment H6 is fused with its reverse complement on the chromatid duplication. Then the second BFB cycle occurs when reverse segment $\overline{H2}$ is fused with its reverse complement on the sister chromatid. Moreover, the third BFB cycle occurs when the breakage happens at segment H4. A sister chromatid is replicated, and reverse segment $\overline{H4}$ on it is fused with segment H4, where the breakage is located. Finally, the FBI on reverse segment $\overline{H3}$ and segment H3 occurs, which contributes to a stable state and indicates the end of this BFB event.

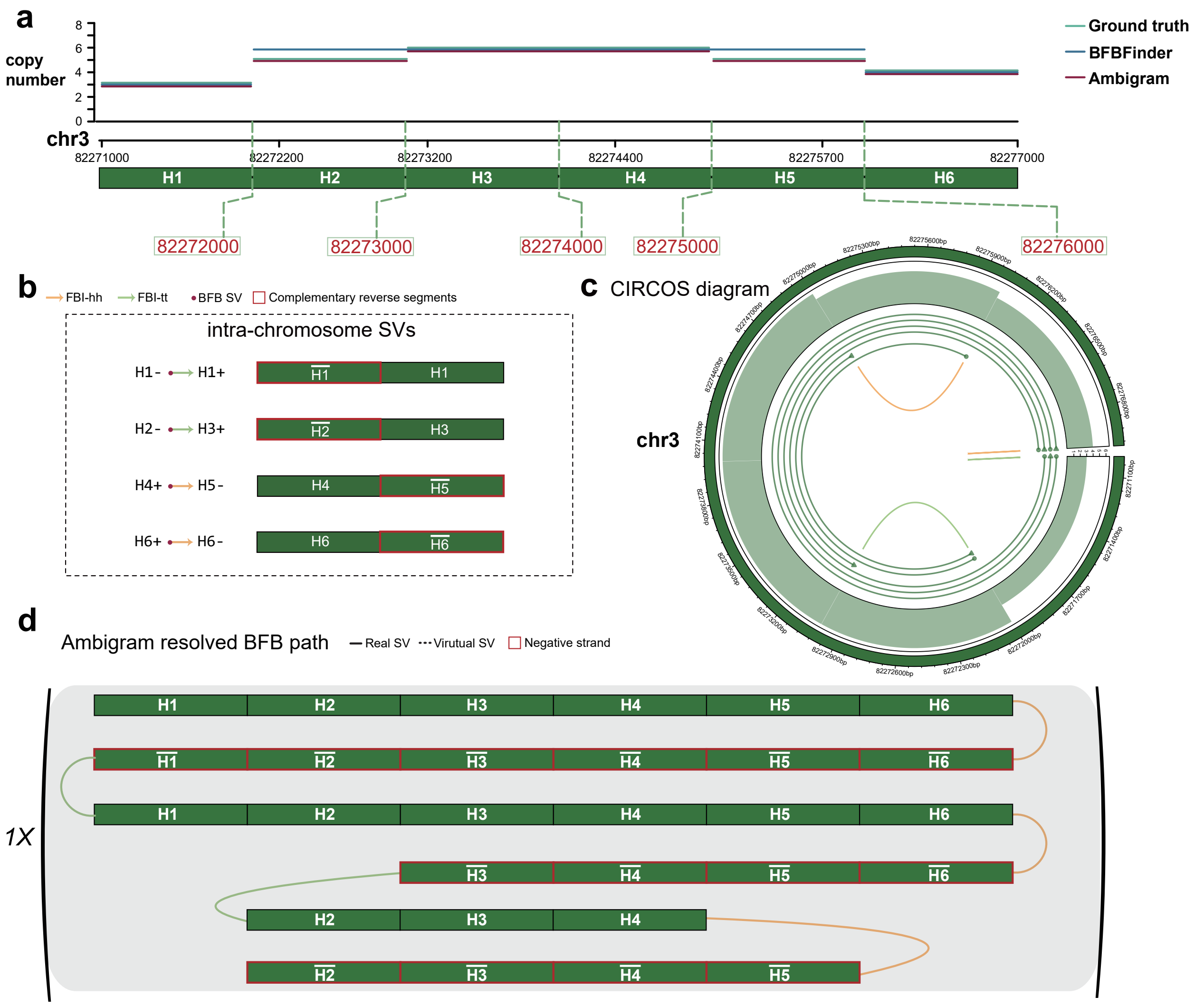

Supplementary Figure 2: Simulated instance 2 - complex BFB involving chr3.
(a) The FBI breakpoints split the local genome region of chr3 into six segments. The stairstep plot shows the ground truth CNs of segments and the CNs derived by BFBFinder and Ambigram. (b) List of FBIs and the segments connected by them. The head-to-head (hh) and tail-to-tail (tt) FBIs are colored light yellow and light green, respectively. The reverse complementary segments have a red border. (c) CIRCOS diagram of the BFB event. The outermost track shows the local genome regions involved with the BFB event. The second outermost track illustrates the input region CNs, and the third track indicates the resolved CNs by Ambigram. Besides, the third track shows the resolved BFB paths, in which the circle and triangle points refer to the 5' end and 3' end, respectively. The innermost part represents all the SVs involved with the BFB event. (d) Ambigram resolved the BFB path. Overall, chr3 undergoes four BFB cycles. The first BFB cycle occurs when segment H6 is fused with its reverse complement on the chromatid duplication. Then the second BFB cycle occurs when the breakpoint on reverse segment $\overline{H1}$ is fused with the left breakpoint on segment $\overline{H1}$ on the sister chromatid. Moreover, the third BFB cycle occurs when the breakage and fusion happen at reverse segment $\overline{H3}$ and segment H2 on its reverse counterpart. Finally, the last cycle occurs when the FBI connects segment H4 to reverse segment $\overline{H5}$, which contributes to a stable state and indicates the end of this BFB event.

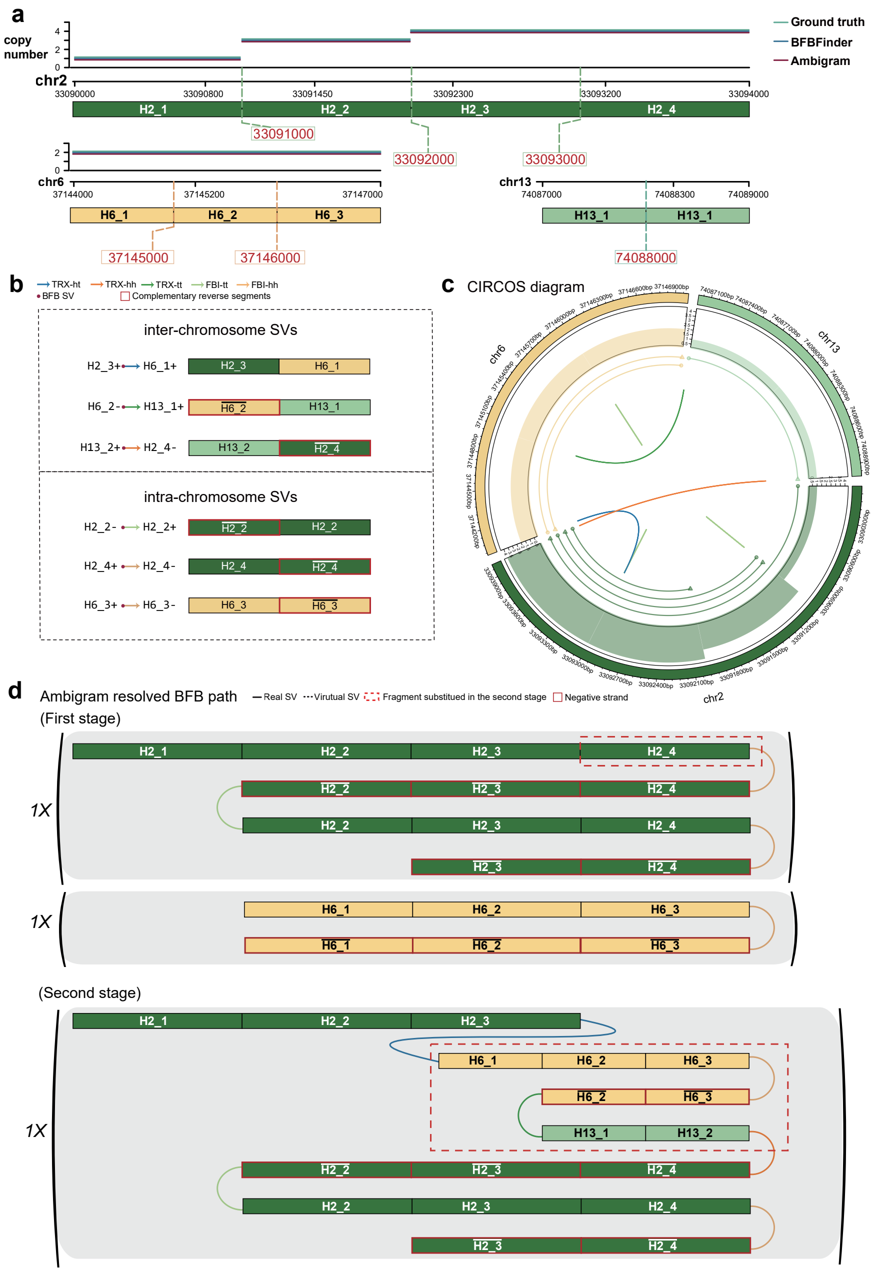

Supplementary Figure 3: Simulated instance 3 - complex BFB involving inter-chromosomal rearrangements on chr2, chr6, and chr13.

(a) The SV breakpoints split the local genome region of chr2 into four segments and partition chr6 and chr13 into 3 and 2 segments, respectively. The stairstep plot shows the ground truth CNs of segments and the CNs derived by BFBFinder and Ambigram. (b) List of SVs and the segments connected by them. The tail-to-tail (tt) FBI is colored light green. The reverse complementary segments have a red border. (c) CIRCOS diagram of the BFB event. The outermost track shows the local genome regions involved with the BFB event. The second outermost track illustrates the input region CNs, and the third track indicates the resolved CNs by Ambigram. Besides, the third track shows the resolved BFB paths, in which the circle and triangle points refer to the 5' end and 3' end, respectively. The innermost part represents all the SVs involved with the BFB event. (d) Ambigram resolved the BFB path. We interpret this BFB with two stages. In the first stage, chr2 undergoes two BFB cycles. The first BFB cycle occurs when segment H2_4 is fused with its reverse complement on the chromatid duplication. Then the second BFB cycle occurs when reverse segment $\overline{H2\_2}$ is fused with its complement on the sister chromatid. Finally, the breakage on reverse segment $\overline{H2\_3}$ contributes to a stable state that indicates the end of the first stage. Besides, chr6 undergoes one BFB cycle that occurs on segment H6_3 and its reverse segment on the sister chromatid. In the second stage, three inter-chromosomal arrangement occurs on chr2, chr6, and chr13, inserting genome regions of chr6 and chr13 into the region between segment H2_2 and reverse segment $\overline{H2\_4}$ on the BFB path of chr2, which leads to the final complex BFB path.

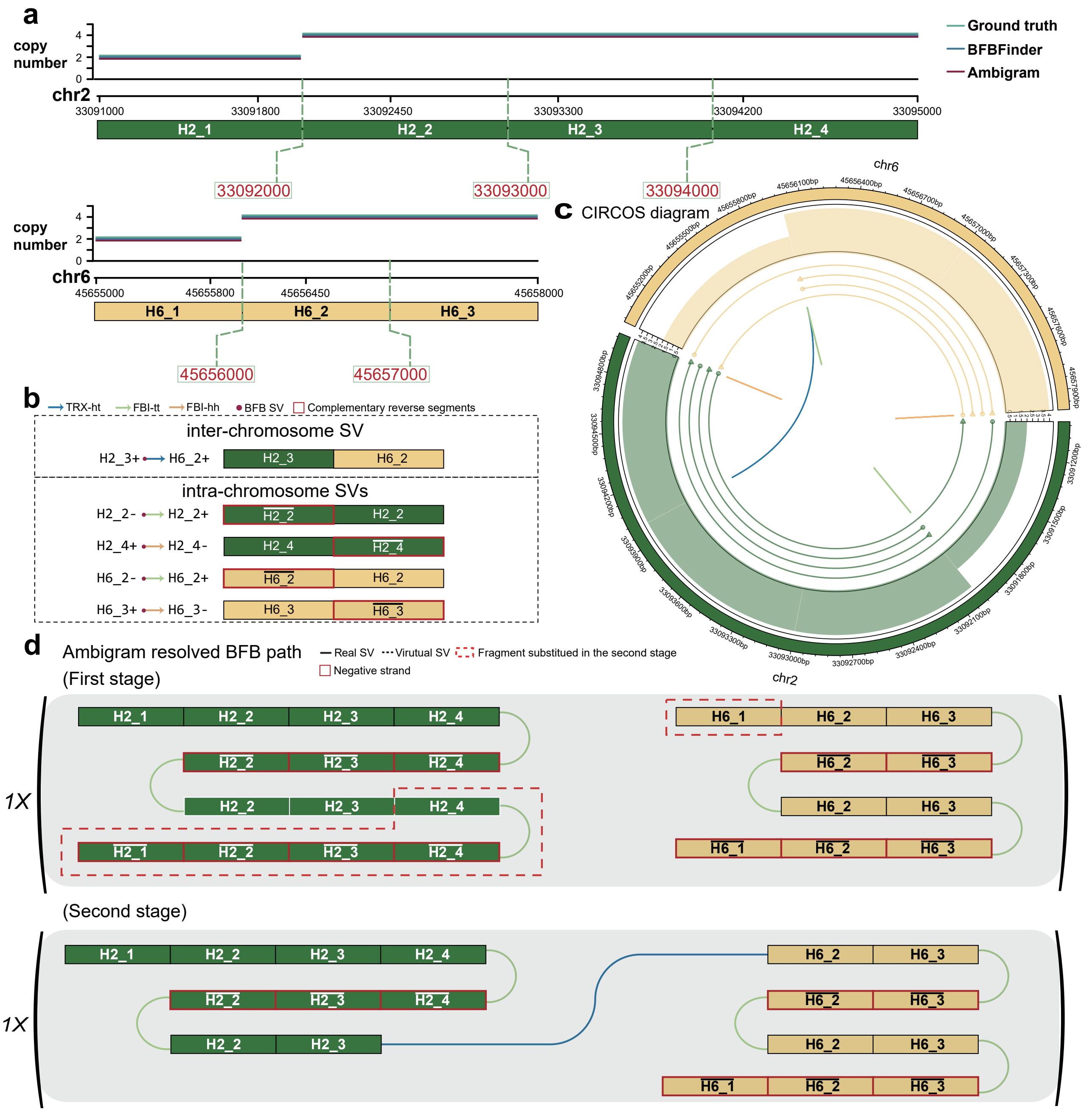

Supplementary Figure 4: Simulated instance 4 - complex BFB involving inter-chromosomal rearrangements on chr2 and chr6.
(a) The SV breakpoints split the local genome region of chr2 into four segments and partition chr6 into three segments. The stairstep plot shows the ground truth CNs of segments and the CNs derived by BFBFinder and Ambigram. (b) List of SVs and the segments connected by them. The tail-to-tail (tt) FBI is colored light green. The reverse complementary segments have a red border. (c) CIRCOS diagram of the BFB event. The outermost track shows the local genome regions involved with the BFB event. The second outermost track illustrates the input region CNs, and the third track indicates the resolved CNs by Ambigram. Besides, the third track shows the resolved BFB paths, in which the circle and triangle points refer to the 5' end and 3' end, respectively. The innermost part represents all the SVs involved with the BFB event. (d) Ambigram resolved the BFB path. We interpret this BFB with two stages. In the first stage, both chr2 and chr6 undergo two BFB cycles. The first BFB cycle for chr2 occurs when segment H2_4 is fused with its reverse complement on the chromatid duplication. Then the second BFB cycle occurs when reverse segment $\overline{H2\_2}$ is fused with its complement on the sister chromatid, contributing to a stable state. Besides, a similar process happens on chr6. The first breakage and fusion happen on segment H6_3 and its reverse complement on the chromatid duplication. Then the second cycle occurs on the FBI breakpoints of reverse segment $\overline{H6\_2}$ and segment H6_2, leading to a stable state. In the second stage, an inter-chromosomal arrangement occurs on chr2 and chr6, which concatenates BFB paths on chr2 and chr6 and indicates the end of this complex BFB event.

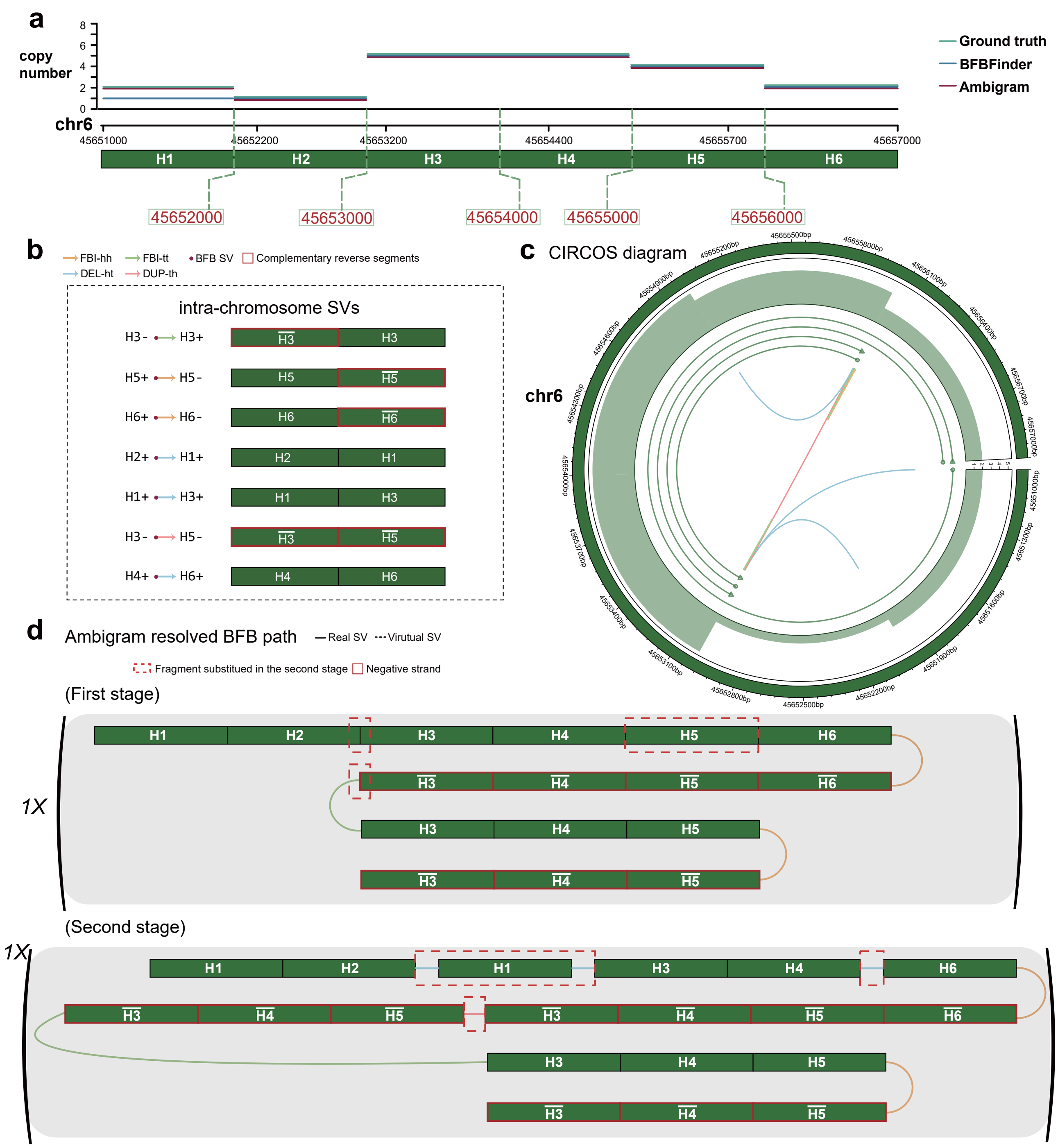

Supplementary Figure 5: Simulated instance 5 - complex BFB involving inter-chromosomal rearrangements on chr6.

(a) The SV breakpoints split the local genome region of chr6 into six segments. The stairstep plot shows the ground truth CNs of segments and the CNs derived by BFBFinder and Ambigram. (b) List of SVs and the segments connected by them. The tail-to-tail (tt) FBI is colored light green, and the head-to-head (hh) FBI is colored light yellow. The reverse complementary segments have a red border. (c) CIRCOS diagram of the BFB event. The outermost track shows the local genome regions involved with the BFB event. The second outermost track illustrates the input region CNs, and the third track indicates the resolved CNs by Ambigram. Besides, the third track shows the resolved BFB paths, in which the circle and triangle points refer to the 5' end and 3' end, respectively. The innermost part represents all the SVs involved with the BFB event. (d) Ambigram resolved the BFB path. We interpret this BFB with two stages. In the first stage, chr6 undergoes three BFB cycles. The first BFB cycle occurs when segment H6 is fused with its reverse complement on the chromatid duplication. Then the second BFB cycle occurs when reverse segment $\overline{H3}$ is fused with its complement on the sister chromatid. Finally, the third cycle occurs on the FBI breakpoints of segment H5 and reverse segment $\overline{H5}$, and the last breakage on reverse segment $\overline{H3}$ leads to a stable state. In the second stage, two SVs insert segment H1 between segment H2 and segment H3, and the other two SVs delete segment H5 and replicate segments H3-H5, respectively.

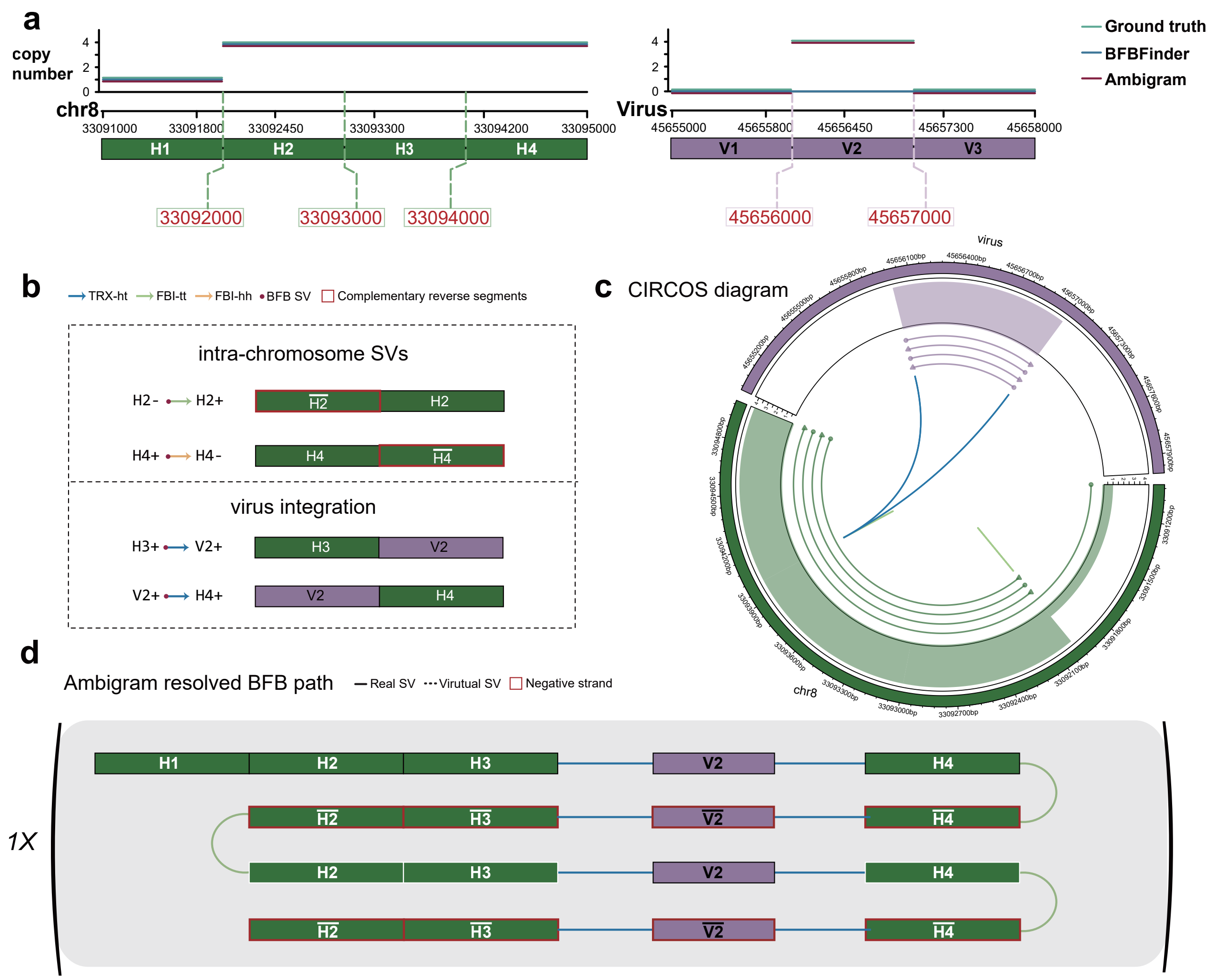

Supplementary Figure 6: Simulated instance 6 - virus-induced complex BFB involving inter-chromosomal rearrangements on chr8 and virus.
(a) The SV breakpoints split the local genome region of chr8 and virus into four and three segments, respectively. The stairstep plot shows the ground truth CNs of segments and the CNs derived by BFBFinder and Ambigram. (b) List of SVs and the segments connected by them. The tail-to-tail (tt) FBI is colored light green, and the head-to-tail (ht) translocation is colored blue. The reverse complementary segments have a red border. (c) CIRCOS diagram of the BFB event. The outermost track shows the local genome regions involved with the BFB event. The second outermost track illustrates the input region CNs, and the third track indicates the resolved CNs by Ambigram. Besides, the third track shows the resolved BFB paths, in which the circle and triangle points refer to the 5' end and 3' end, respectively. The innermost part represents all the SVs involved with the BFB event. (d) Ambigram resolved the BFB path. We interpret this BFB with two stages. In the first stage, segment v2 on virus is inserted between segment H3 and segment H4 on chr8. In the second stage, the virus-integrated chromosome undergoes two BFB cycles. The first BFB cycle occurs when segment H4 is fused with its reverse complement on the chromatid duplication. Then the second BFB cycle occurs when reverse segment $\overline{H2}$ is fused with its complement on the sister chromatid. Finally, the breakage on reverse segment $\overline{H2}$ contributes to a stable state.

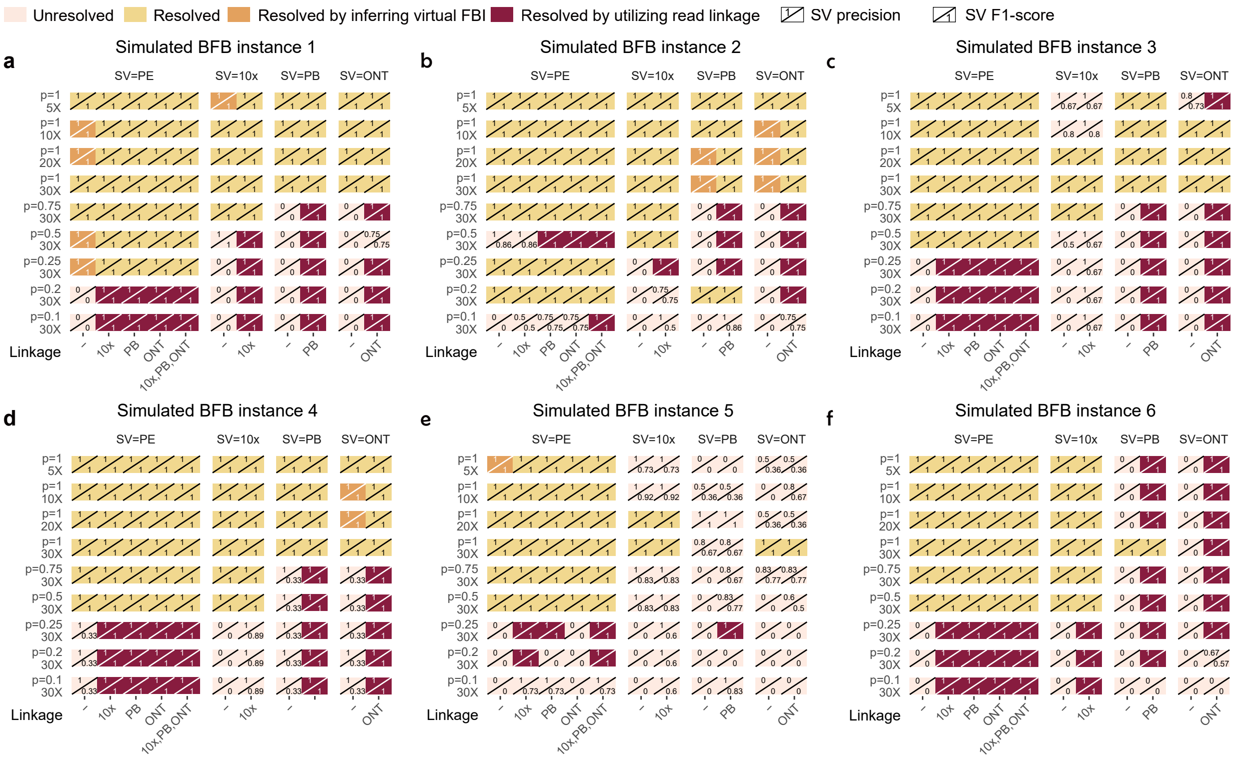

Supplementary Figure 7: The SV precision and SV F1-score of Ambigram for six simulated BFB instances. (a-f) Results derived by Ambigram for simulated BFB instances 1-6 with various sequencing protocols, depths, and purities. "-" means that the inputs of Ambigram are SVs called from one sequencing protocol (SV=PE, SV=10x, SV=PB, or SV=ONT) and ground truth CNs. "Resolved" means all SVs and CNs from the inferred BFB path are matched with those of ground truths, otherwise "Unresolved". "Resolved by inferring the virtual FBI" signifies Ambigram resolves the BFB path by recovering the undetected FBIs in low sequencing depth and tumor purity scenarios. "Resolved by utilizing read linkage" means that Ambigram cannot resolve the BFB path with CNs and detected SVs, while it can resolve the path after incorporating the linked or long read linkage from 10x, PB, or ONT data. The SV precision measures the portion of the SVs inferred correctly among predictions, that is, the number of ground truth SVs inferred correctly by the tool over the total number of inferred SVs. The SV F1-score is the harmonic mean of SV precision and SV recall. Note that "Resolved" implies CN accuracy = 1, SV precision = 1, SV recall = 1, and SV F1-score= 1.

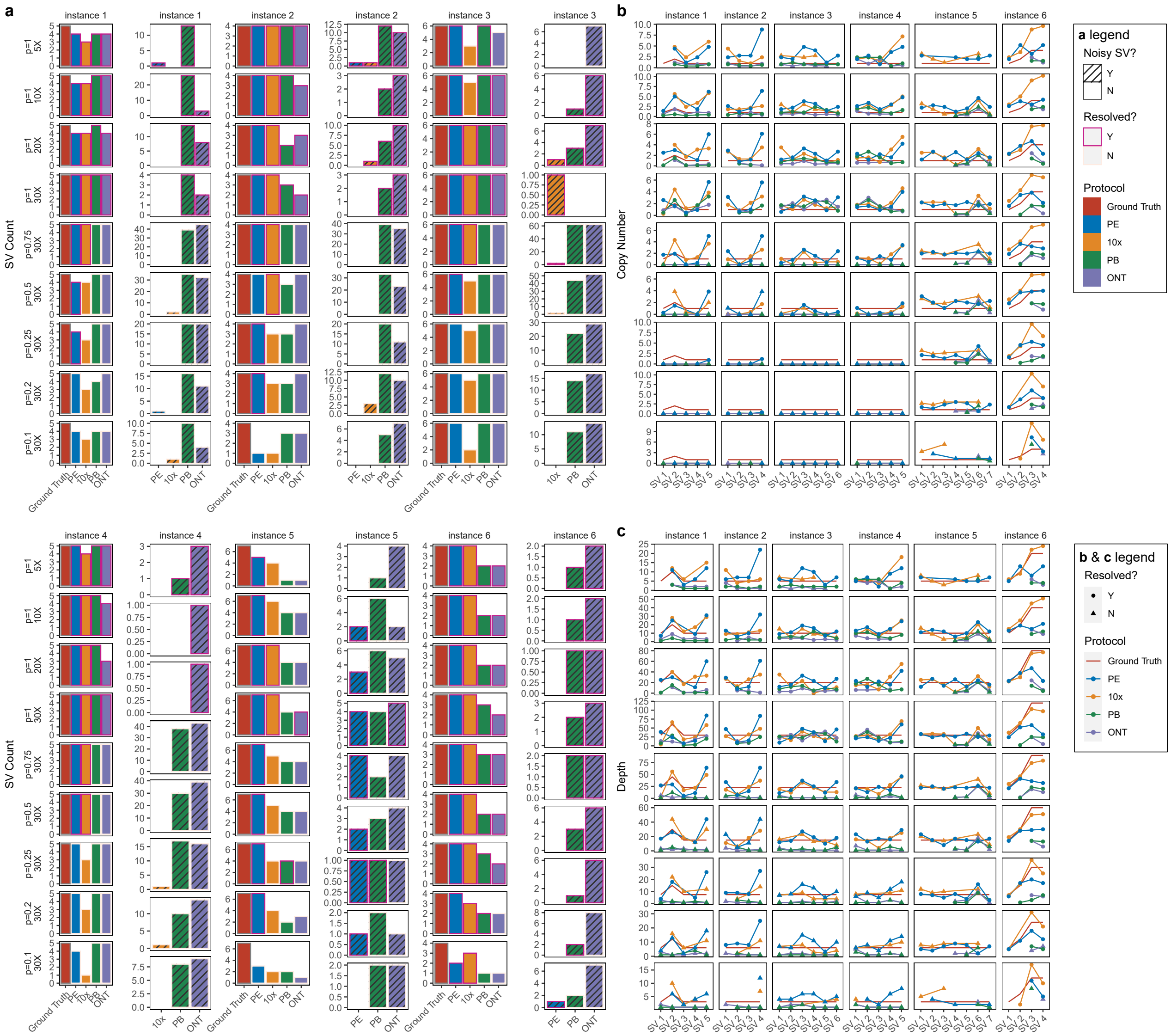

Supplementary Figure 8: The SV and CN statistics of six simulated BFB instances.
(a) The number of noisy and BFB SVs from ground truths and called from PE, 10x, PB, and ONT sequencing reads. (b) The copy number of BFB SVs from ground truths, PE, 10x, PB, and ONT sequencing reads. These copy numbers are used as Ambigram input. (c) The depth of BFB SVs from ground truths, PE, 10x, PB, and ONT sequencing reads. These depths are used to estimate the Ambigram input copy numbers.

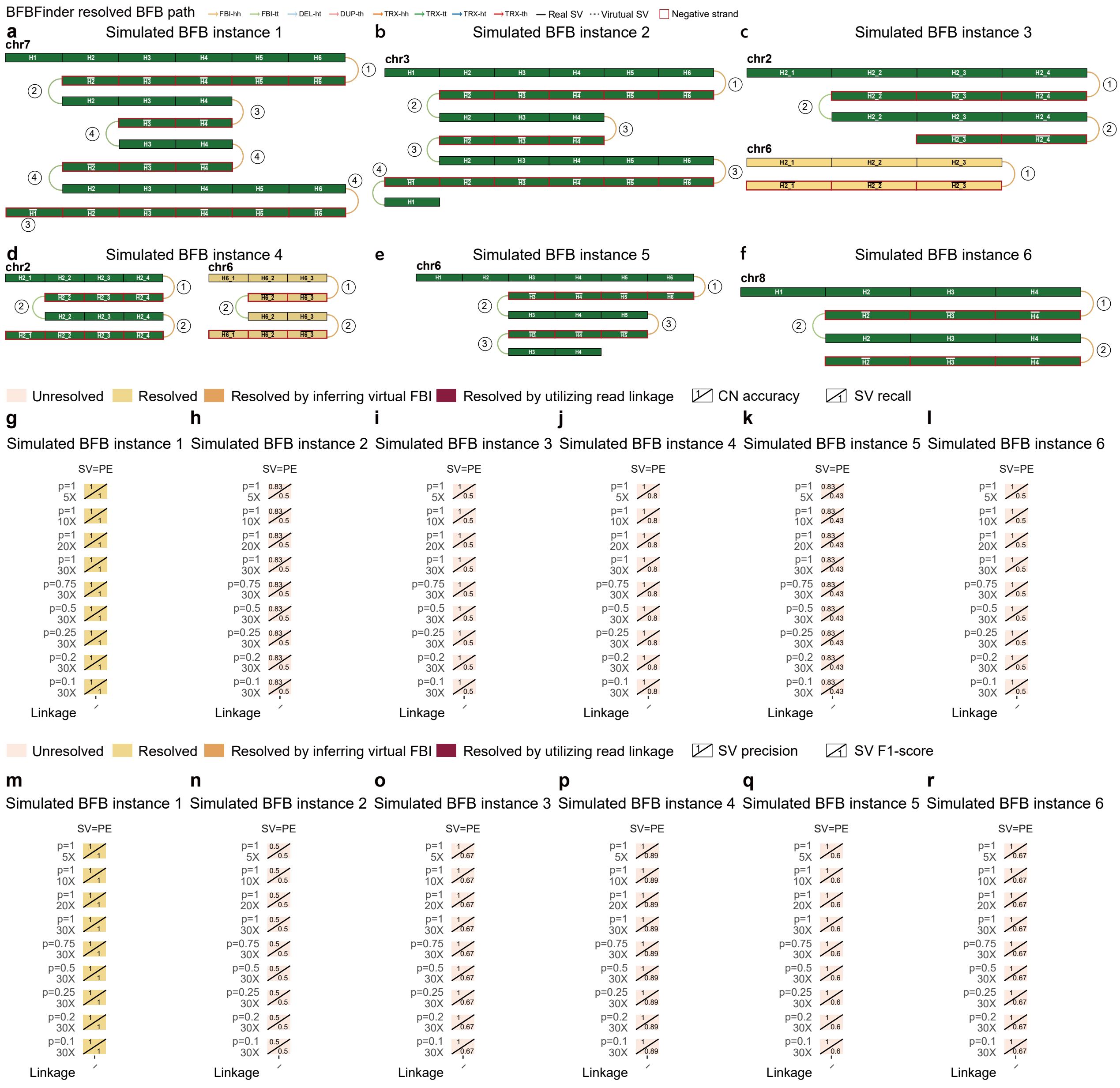

Supplementary Figure 9: Benchmarking BFBFinder with *in silico* data
(a-f) The BFB paths from BFBFinder of simulated instances 1-6. The number n in a circle denotes that the SV comes from chromosome duplication in the n-th BFB cycle. (g-r) Results derived by BFBFinder for simulated BFB instances 1-6 with various sequencing depths and purities. "-" means that the inputs of BFBFinder are SVs called from one sequencing protocol and ground truth CNs. "Resolved" means all SVs and CNs from the inferred BFB path are matched with those of ground truths, otherwise "Unresolved". "Resolved by inferring the virtual FBI" signifies BFBFinder resolves the BFB path by recovering the undetected FBIs in low sequencing depth and tumor purity scenarios. "Resolved by utilizing read linkage" means that BFBFinder cannot resolve the BFB path with CNs and detected SVs, while it can resolve the path after incorporating the linked or long read linkage from 10x, PB, or ONT data. The CN accuracy is measured by the number of segments with correctly inferred CNs divided by the total segment number. The SV precision measures the portion of the SVs inferred correctly among predictions, that is, the number of ground truth SVs inferred correctly by the tool over the total number of inferred SVs. The SV recall measures the portion of the SVs inferred correctly among ground truths, that is, the number of ground truth SVs inferred correctly by the tool over the total number of ground truth SVs. The SV F1-score is the harmonic mean of SV precision and SV recall. Note that an instance is resolved only if CN accuracy, SV precision, SV recall, and SV F1-score are 1. BFB: breakage-fusion-bridge. FBI-hh: fold-back inversion with head-to-head direction. FBI-tt: fold-back inversion with tail-to-tail direction. DEL: deletion. DUP: duplication. INS: insertion. TRX: translocation. SV: structure variation. PE: paired-end. 10x: 10x linked-reads. PB: PacBio. ONT: Oxford Nanopore.

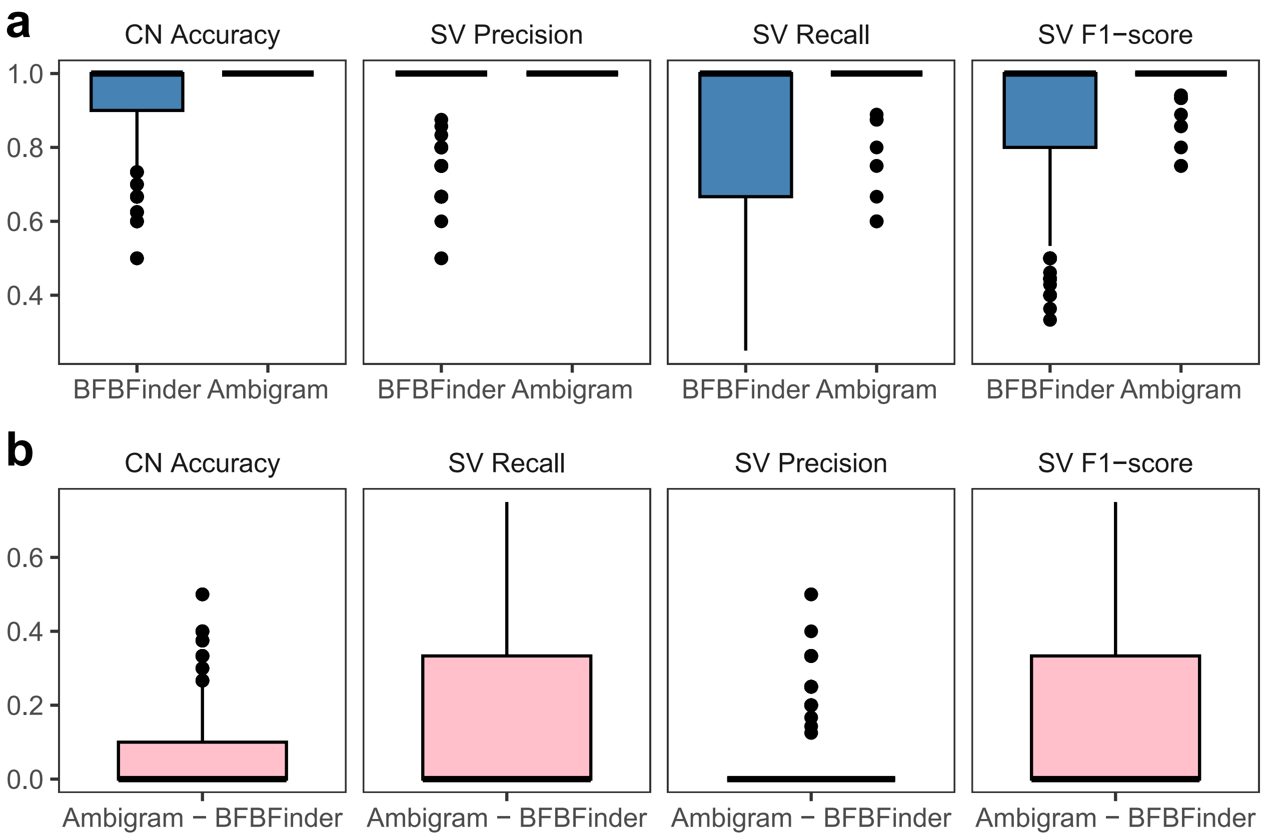

Supplementary Figure 10: The CN accuracy, SV precision, SV recall, and SV F1-score of Ambigram and BFBFinder for 410 simulated BFB paths.
(a) The CN accuracy, SV Precision, SV Recall, and SV F1-score of BFBFinder and Ambigram for 410 simulation instances. (b) The CN accuracy, SV Precision, SV Recall, and SV F1-score change of Ambigram against BFBFinder for 410 simulation instances. We have $n = 410$ simulated BFB instances in total. The CN accuracy is measured by the number of segments with correctly inferred CNs divided by the total segment number. The SV precision measures the portion of the SVs inferred correctly among predictions, that is, the number of ground truth SVs inferred correctly by the tool over the total number of inferred SVs. The SV recall measures the portion of the SVs inferred correctly among ground truths, that is, the number of ground truth SVs inferred correctly by the tool over the total number of ground truth SVs. The SV F1-score is the harmonic mean of SV precision and SV recall. Box plots indicate the median (middle line), 25th, 75th percentile (box), and 5th and 95th percentile (whiskers) as well as outliers (single points).

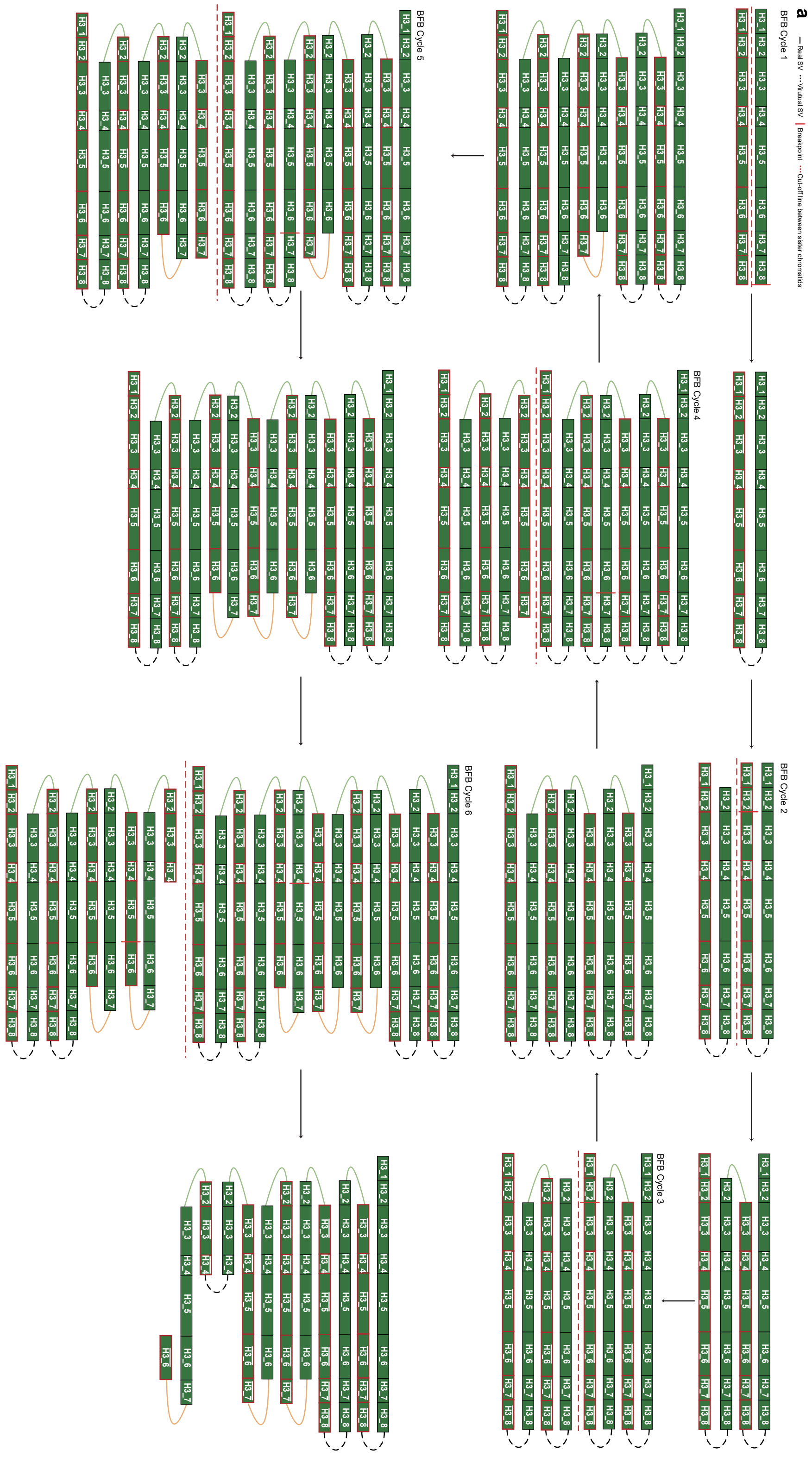

Supplementary Figure 11: COLO829 instance 1 - the explicit evolution process of complex BFB in the first stage.

The whole complex BFB event is fully illustrated in Fig. 3 and this supplementary figure. This figure mainly illustrates the first stage, when chr3 and chr6 encounter six and four BFB cycles, respectively. The first BFB cycle on chr3 occurs when a sister chromatid is replicated, and segment H3_8 is fused with its reverse complement. Then the second BFB cycle starts when the double-strand breaks off at reverse segment $\overline{H3\_3}$, which leads to instability of chr3. A duplication is reproduced, and reverse segment $\overline{H3\_3}$ is fused with segment H3_2. Similarly, chr3 undergoes the third BFB cycle that happens on segments $\overline{H3\_3}$-H3_2. Following that, the fourth BFB cycle happens at the breakage point on segment H3_6, fused with reverse segment $\overline{H3\_7}$ on the chromatid duplication. Furthermore, a similar BFB cycle occurs on segments H3_6-$\overline{H3\_7}$. Finally, another breakage occurs at segment H3_4, and a sister chromatid is replicated. The final BFB cycle fuses segment H3_4 and reverse segment $\overline{H3\_4}$, and another breakage on reverse segment $\overline{H3\_6}$ leads to a stable state, which indicates the end of the BFB event on chr3. Similarly, chr6 undergoes four BFB cycles on breakpoints of segments H6_4, $\overline{H6\_1}$, $\overline{H6\_3}$, and H6_2 in order.

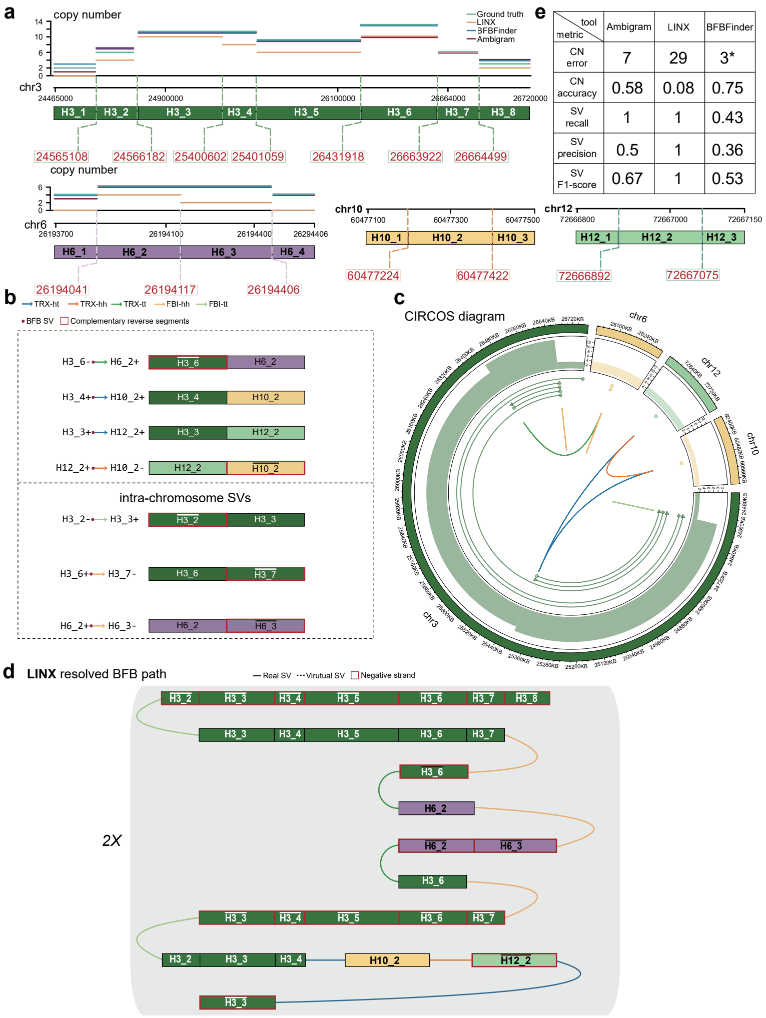

tool / metric	Ambigram	LINX	BFBFinder
CN error	7	29	3*
CN accuracy	0.58	0.08	0.75
SV recall	1	1	0.43
SV precision	0.5	1	0.36
SV F1-score	0.67	1	0.53

Supplementary Figure 12: COLO829 instance 1 - complex BFB solved by LINX.
(a) The SV breakpoints split the local genome region of chr3 into eight segments and partition chr6, chr10, and chr20 into 4, 3, and 3 segments, respectively. The stairstep plot shows the ground truth CNs of segments and the CNs derived by Ambigram and LINX. (b) List of SVs and the segments connected by them. The head-to-head (hh) and tail-to-tail(tt) FBIs are colored light yellow and light green, respectively. The reverse complementary segments have a red border. (c) CIRCOS diagram of the complex BFB. The outermost track shows the local genome regions involved with the BFB event. The second outermost track illustrates the input region CNs, and the third track indicates the resolved CNs by Ambigram. Besides, the third track shows the resolved BFB paths, in which the circle and triangle points refer to the 5' end and 3' end, respectively. The innermost part represents all the SVs involved with the BFB event. (d) LINX resolved the BFB path. The first BFB cycle starts when the breakage happens at reverse complementary segment H3_2. Then chr3 replicates its sister chromatid spanning from segment H3_3 to segment H3_8, and two sister chromatids are fused. Following that, the double-strand breaks off at segment H3_7, a sister chromatid is reproduced, and segment H3_7 is fused with reverse segment $\overline{H3\_6}$. Moreover, another breakage occurs at reverse segment $\overline{H3\_6}$, connected to H6_2 on chr6 with an inter-chromosomal rearrangement. Then the third BFB cycle happens when duplication and fusion occur at segment H6_2 and the reverse complementary segment H6_3. Furthermore, the fourth BFB cycle occurs when the double-strand breaks off at segment H3_4, and a sister chromatid is replicated. Finally, fragments of chr10 and chr12 are inserted between segment H3_4 and reverse segment $\overline{H3\_3}$, and a stable state is achieved after the final breakage occurs at reverse segment $\overline{H3\_3}$. Since chromosomal duplication follows these 4 BFB cycles through a whole genome doubling event, the final BFB path gets two copies. (e) The total CN error, CN accuracy, and SV recall derived by Ambigram, LINX, and BFBFinder compared to ground truth. The total CN error is the sum of all segment copy number differences between the output and ground truth. BFBFinder is marked with "*" as BFBFinder merely accepts segment CN profiles from a single chromosome, so we fit the ground truth CN profiles of BFB paths of chr3 and chr6 separately. The CN accuracy is measured by the number of segments with correctly inferred CNs divided by the total segment number. The SV recall measures the portion of the SVs inferred correctly among ground truths, that is, the number of ground truth SVs inferred correctly by the tool over the total number of ground truth SVs. Although BFBFinder had a small CN error, it failed to resolve the complex BFB event with translocation.

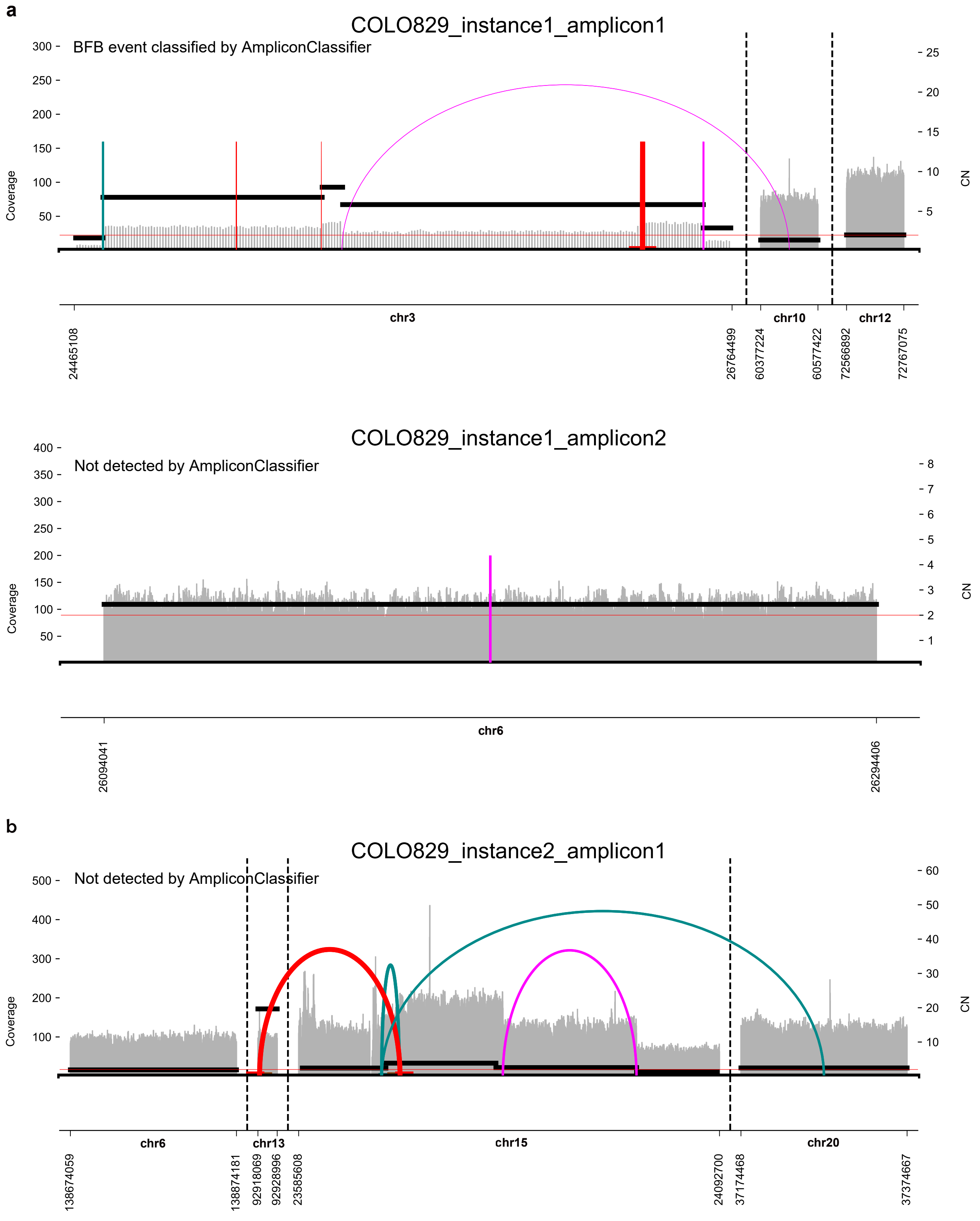

Supplementary Figure 13: COLO829 detected in AmpliconArchitect and AmpliconClassifier. (a) COLO829 instance 1 is interpreted as two separate amplicons by AmpliconArchitect. The first amplicon, involved with chr3, chr10, and chr12, is classified as a BFB event by AmpliconClassifier. The second amplicon on chr6 is not detected as BFB. (b) COLO829 instance 2 is interpreted as an amplicon by AmpliconArchitect, but it is not classified as a BFB event by AmpliconClassifier.

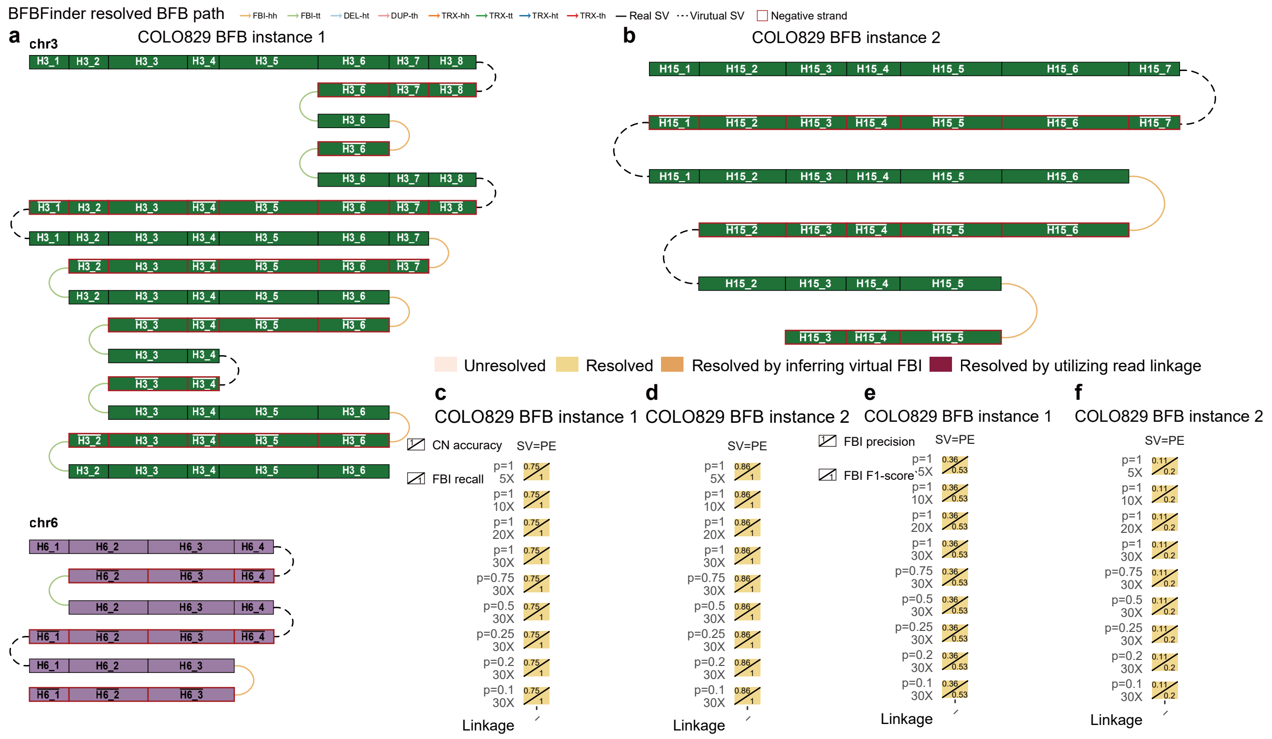

Supplementary Figure 14: Results of COLO829 instances from BFBFinder.
(a-b) The BFB paths from BFBFinder of COLO829 instances. (c-f) Results derived by BFBFinder for COLO829 cases with various sequencing depths and purities. "-" means that the inputs of BFBFinder are SVs called from one sequencing protocol and ground truth CNs. "Resolved" means all FBIs from the inferred BFB path are matched with those of ground truths, otherwise "Unresolved". "Resolved by inferring the virtual FBI" signifies BFBFinder resolves the BFB path by recovering the undetected FBIs in low sequencing depth and tumor purity scenarios. "Resolved by utilizing read linkage" means that BFBFinder cannot resolve the BFB path with CNs and detected SVs, while it can resolve the path after incorporating the linked or long read linkage from 10x, PB, or ONT data. The CN accuracy is measured by the number of segments with correctly inferred CNs divided by the total segment number. The SV precision measures the portion of the SVs inferred correctly among predictions, that is, the number of ground truth SVs inferred correctly by the tool over the total number of inferred SVs. The SV recall measures the portion of the SVs inferred correctly among ground truths, that is, the number of ground truth SVs inferred correctly by the tool over the total number of ground truth SVs. The SV F1-score is the harmonic mean of SV precision and SV recall. Note that an instance is resolved only if the FBI recall is 1. BFB: breakage-fusion-bridge. FBI-hh: fold-back inversion with head-to-head direction. FBI-tt: fold-back inversion with tail-to-tail direction. DEL: deletion. DUP: duplication. INS: insertion. TRX: translocation. SV: structure variation. PE: paired-end. 10x: 10x linked-reads. PB: PacBio. ONT: Oxford Nanopore.

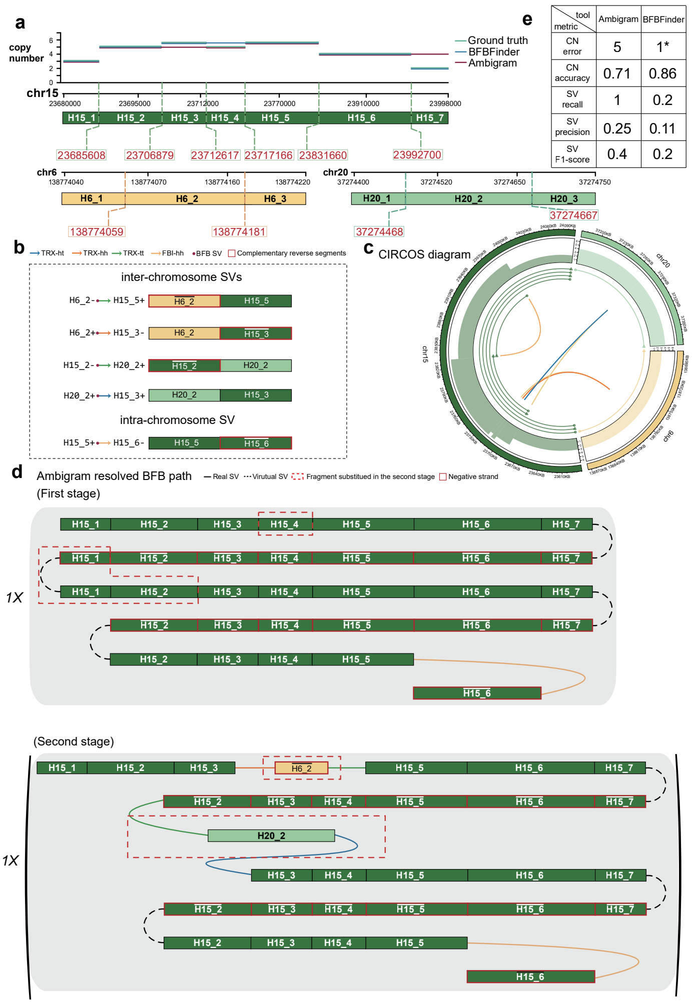

Supplementary Figure 15: COLO829 instance 2 - complex BFB involving inter-chromosomal rearrangements on chr15, chr6, and chr20.
(a) The SV breakpoints split the local genome region of chr15 into seven segments and partition chr6 and chr20 into three segments, respectively. The stairstep plot shows the ground truth CNs of segments and the CNs derived by Ambigram. (b) List of SVs and the segments connected by them. The head-to-head (hh) FBI is colored light yellow. The reverse complementary segments have a red border. (c) CIRCOS diagram of the complex BFB. The outermost track shows the local genome regions involved with the BFB event. The second outermost track illustrates the input region CNs, and the third track indicates the resolved CNs by Ambigram. Besides, the third track shows the resolved BFB paths, in which the circle and triangle points refer to the 5' end and 3' end, respectively. The innermost part represents all the SVs involved with the BFB event. (d) Ambigram resolved the BFB path. We interpret this BFB with two stages. In the first stage, chr15 undergoes four BFB cycles. The first BFB cycle occurs when segment H15_7 is fused with its reverse complement on the chromatid duplication. Then the second BFB cycle occurs when reverse segment $\overline{H15\_1}$ is fused with its complement on the sister chromatid. Furthermore, the third BFB cycle occurs when the breakage happens at reverse segment $\overline{H15\_2}$. Following that, a sister chromatid is replicated, and segments $\overline{H15\_2}$ and H15_2 are fused. Finally, the fourth BFB cycle fuses segments H15_5-$\overline{H15\_6}$, and another breakage at reverse segment $\overline{H15\_6}$ contributes to a stable state that indicates the end of the first stage. In the second stage, an inter-chromosomal arrangement occurs on chr15 and chr6, inserting reverse segment $\overline{H6\_2}$ into the region between segments H15_3 and H15_5. Besides, another translocation links chr15 and chr20 by connecting segment H20_2 to reverse segment $\overline{H15\_2}$ and segment H15_3 on the BFB path of ch15, which leads to the final complex BFB path. (e) The total CN error, CN accuracy, and SV recall derived by Ambigram and BFBFinder compared to ground truth. The total CN error is the sum of all segment copy number differences between the output and ground truth. BFBFinder is marked with "*" as BFBFinder merely accepts segment CN profiles from a single chromosome, so we fit the ground truth CN profiles of BFB paths of chr15. The CN accuracy is measured by the number of segments with correctly inferred CNs divided by the total segment number. The SV recall measures the portion of the SVs inferred correctly among ground truths, that is, the number of ground truth SVs inferred correctly by the tool over the total number of ground truth SVs. Although BFBFinder had a small CN error, it failed to resolve the complex BFB event with translocation.

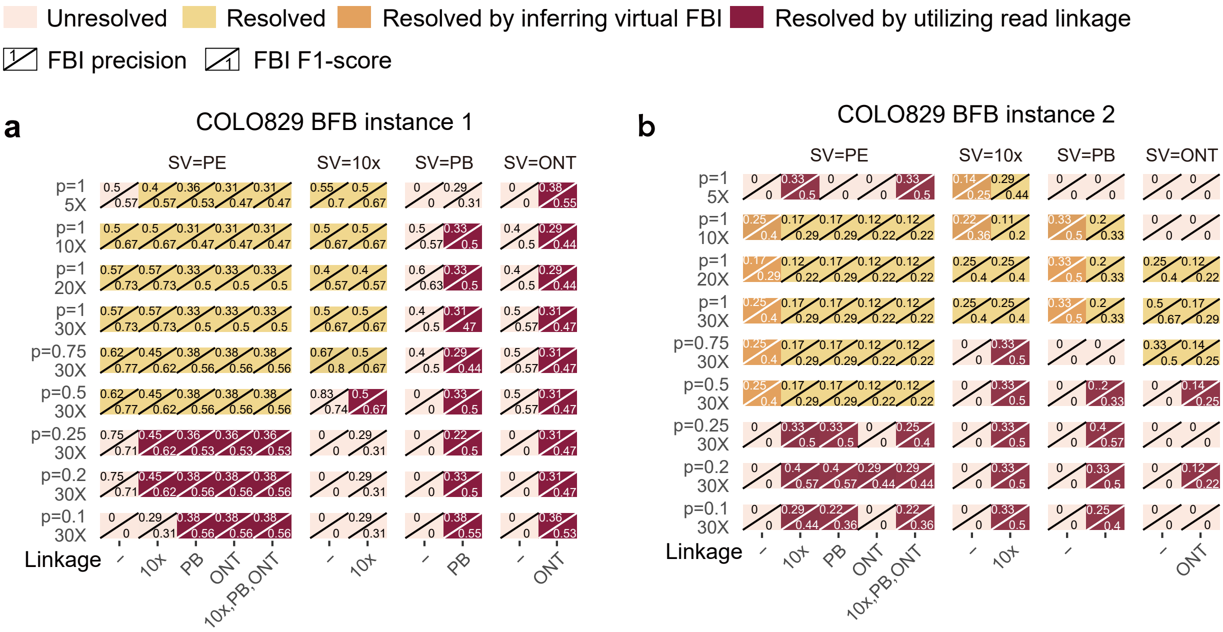

Supplementary Figure 16: The FBI precision and FBI F1-score of Ambigram for two BFB instances on COLO829.

(a-b) Results derived by Ambigram for COLO829 instances 1-2 with various sequencing protocols, depths, and purities. "-" means that the inputs of Ambigram are SVs called from one sequencing protocol (SV=PE, SV=10x, SV=PB, or SV=ONT) and ground truth CNs. "Resolved" means that inferred BFB path includes all ground truth FBIs, otherwise "Unresolved". "Resolved by inferring the virtual FBI" signifies Ambigram resolves the BFB path by recovering the undetected FBIs in low sequencing depth and tumor purity scenarios. "Resolved by utilizing read linkage" means that Ambigram cannot resolve the BFB path with CNs and detected SVs, while it can resolve the path after incorporating the linked or long read linkage from 10x, PB, or ONT data. The CN accuracy is measured by the number of segments with correctly inferred CNs divided by the total segment number. The FBI precision measures the portion of the FBIs inferred correctly among predictions, that is, the number of ground truth FBIs inferred correctly by the tool over the total number of inferred FBIs. The FBI recall measures the portion of the FBIs inferred correctly among ground truths, that is, the number of ground truth FBIs inferred correctly by the tool over the total number of ground truth FBIs. The FBI F1-score is the harmonic mean of FBI precision and FBI recall. Note that an instance is resolved only if FBI recall = 1.

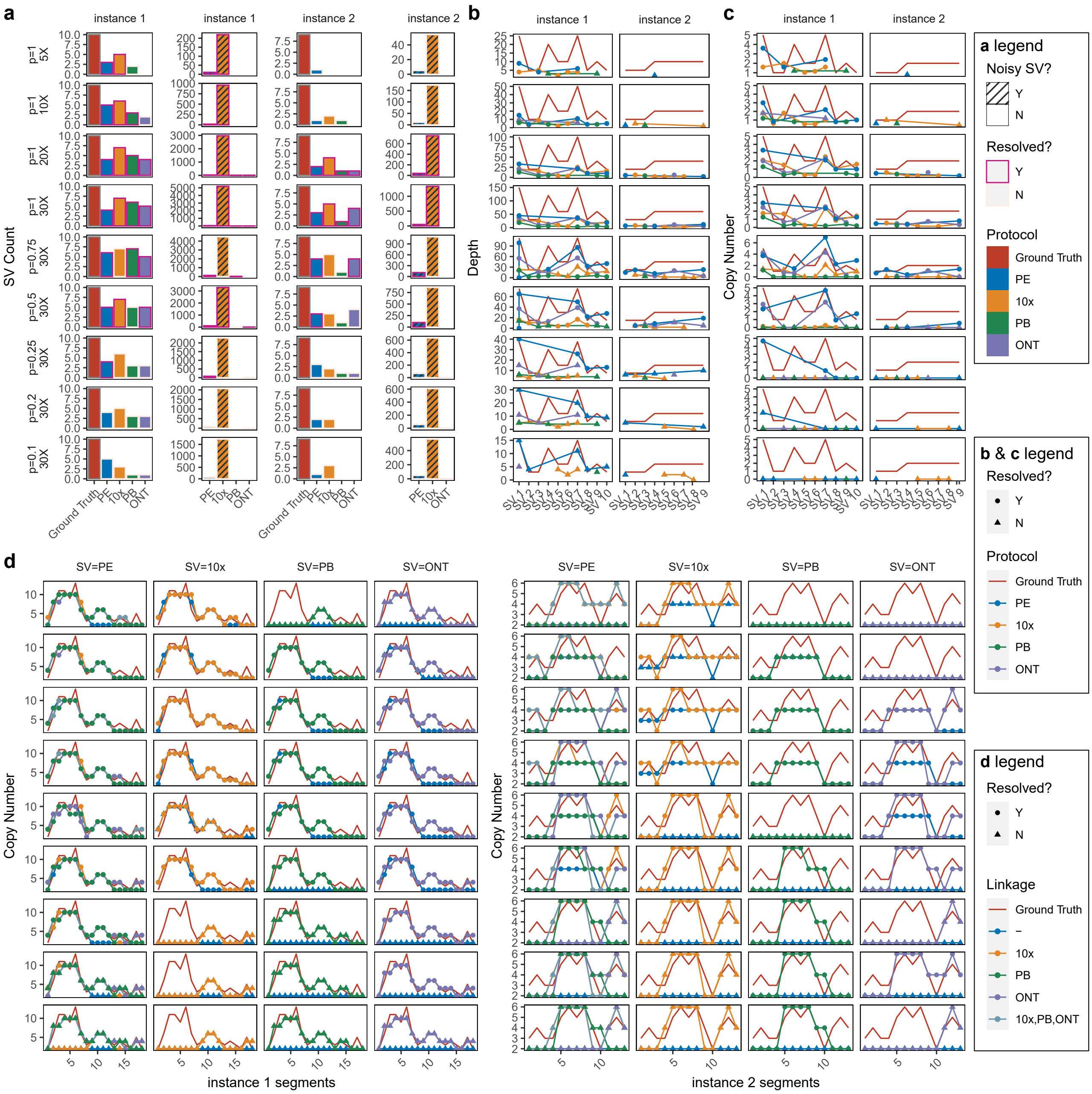

Supplementary Figure 17: The SV and CN statistics of two BFB instances on COLO829. (a) The number of noisy and BFB SVs from ground truths and called from PE, 10x, PB, and ONT sequencing reads. (b) The depth of BFB SVs from ground truths, PE, 10x, PB, and ONT sequencing reads. These depths are used to estimate the Ambigram input copy numbers. (c) The copy number of BFB SVs from ground truths, PE, 10x, PB, and ONT sequencing reads. These copy numbers are used as Ambigram input. (d) The ground truth copy number and Ambigram predicted copy number for each genome segment from PE, 10x, PB, and ONT sequencing reads.

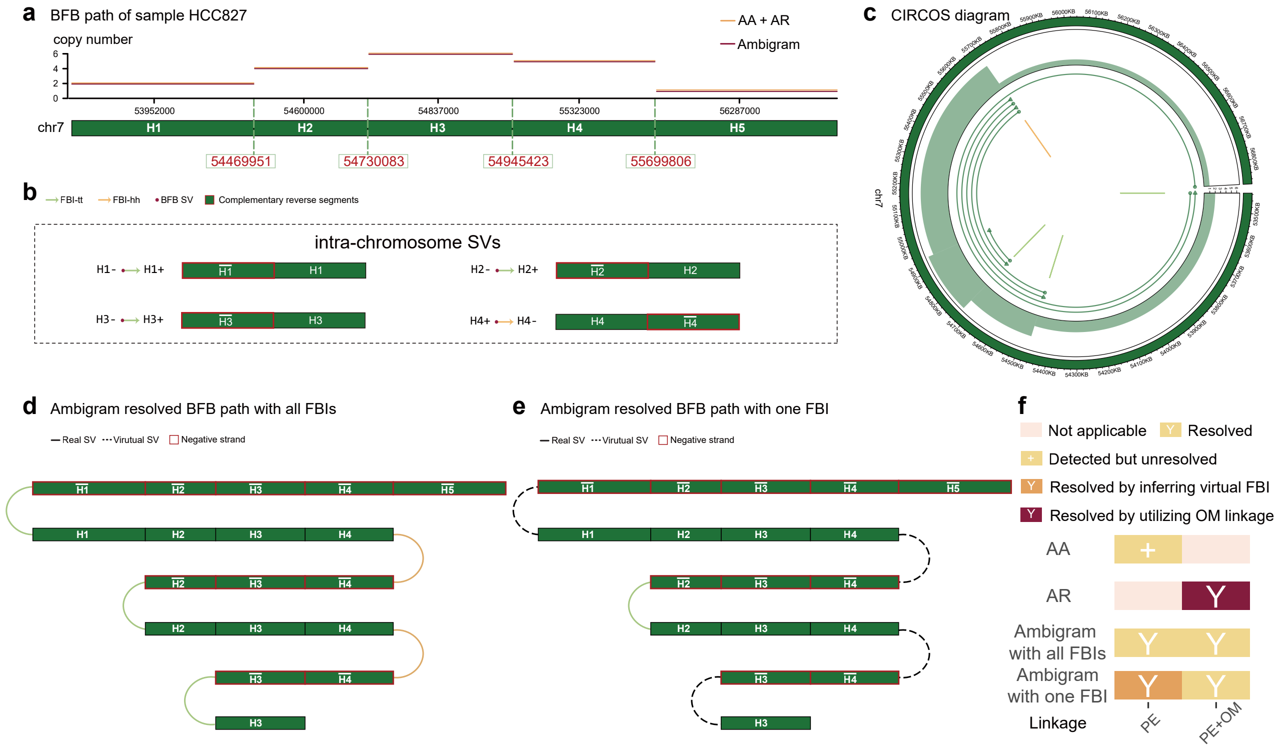

Supplementary Figure 18: The BFB event in a lung cancer sample HCC827 [2].
(a) The SV breakpoints split the local genome region of chr7 into five segments. The stairstep plot shows the ground truth CNs of segments and the CN derived by Ambigram and a combination of AA and AR. (b) List of SVs and the segments they connect. The head-to-head (hh) and tail-to-tail(tt) FBIs are colored light orange and light green, respectively. The reverse complementary segments have a red border. The SV called from PE sequencing data is marked with a red circle, while SVs from OM data are marked with blue circles. (c) CIRCOS diagram of the complex BFB. The outermost track shows the local genome regions involved with the BFB event. The second outermost track illustrates the input region CNs, and the third track indicates the resolved CNs by Ambigram. Besides, the third track shows the resolved BFB paths, in which the circle and triangle points refer to the 5' end and 3' end, respectively. The innermost part represents all the SVs involved with the BFB event. (d) Ambigram resolved the BFB path with all FBIs. In this BFB event, chr7 undergoes four BFB cycles. The first cycle occurs when a sister chromatid is replicated, and reverse segment $\overline{H1}$ is fused with its complement H1; then, the second BFB cycle fuses segments H4 and $\overline{H4}$; moreover, the third cycle starts when breakage occurs on reverse segment $\overline{H2}$, and segments $\overline{H2}$ is fused with H2; finally, the fourth BFB cycle fuses reverse segment H3 and its reverse complement, and another breakage on segment H3 results in a stable BFB path on chr7. The final BFB path is the same as the result of [2]. (e) Ambigram resolved the BFB path with only one FBI. To test the robustness of Ambigram, we only input one FBI on segments H2 and $\overline{H2}$ to resolve a BFB path. The result from Ambigram is the same as (d), while all FBIs, except the FBI H2-$\overline{H2}$, in (d) are inferred as virtual FBIs. (f) Results derived by AA, AR, Ambigram with all FBIs, and Ambigram with one FBI.

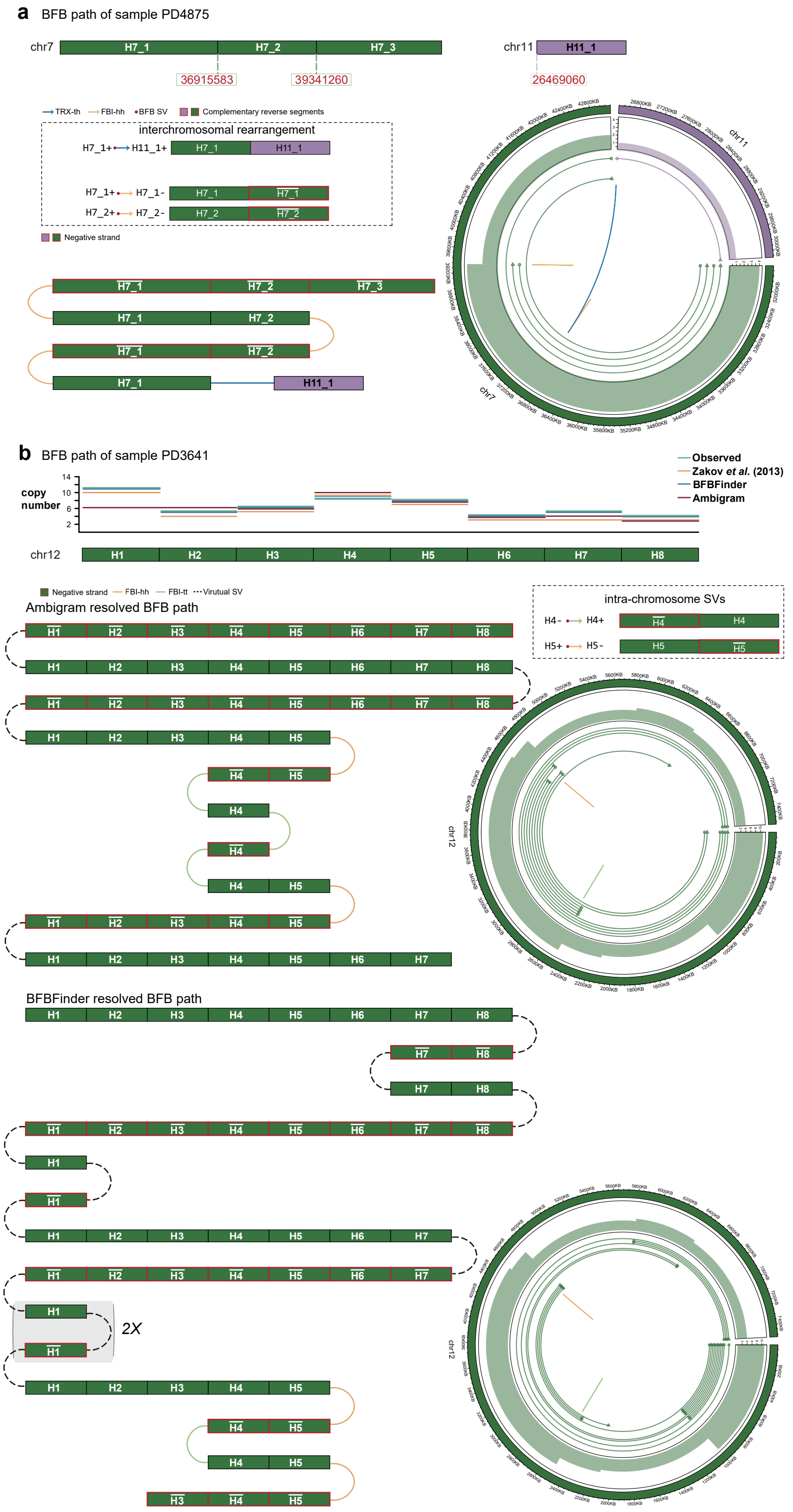

Supplementary Figure 19: BFB events in breast cancer sample PD4875 [3] and pancreatic cancer sample PD3641 [4].

(a) The SV breakpoints split the local genome region of chr7 into H1, H2, and H3 segments. The middle layer shows translocation and FBIs involved in these complex BFB events. The bottom layer illustrates Ambigram solved BFB path in sample PD4875. (b) The top layer shows various CNs of segments on chr12 in sample PD3641, according to the observed CNs, results from Zakov et al. (2013) [4], BFBFinder [17], and Ambigram. The middle layer illustrates Ambigram solved BFB path in sample PD3641, which undergoes five BFB cycles. The bottom layer illustrates BFBFinder solved BFB path (the first result of BFBFinder) in sample PD3641, which undergoes seven BFB cycles. The CIRCOS diagrams on the right reflect the CNs, SVs, and the output local genomic map.

a COLO829

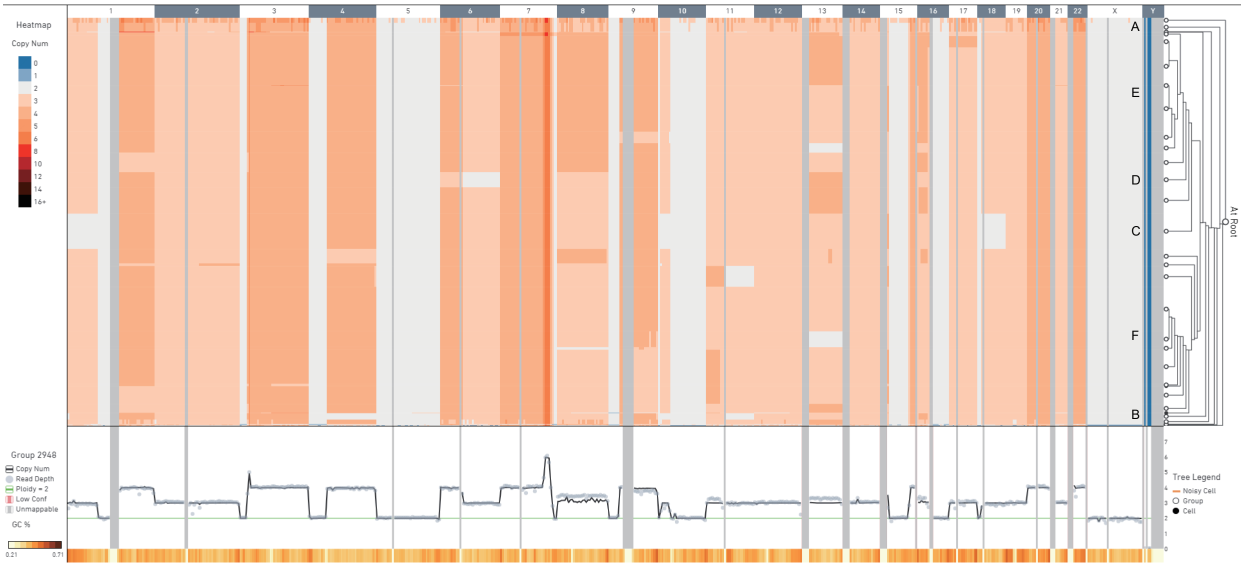

b MKN45

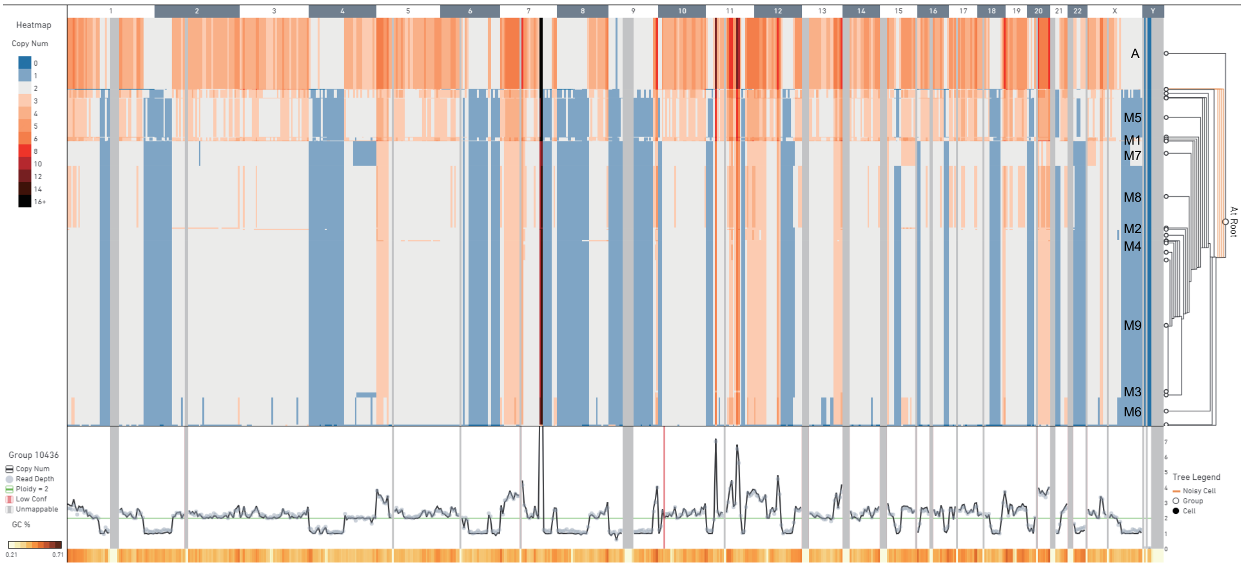

Supplementary Figure 20: (a-b) The copy number profiles of single cells from cell lines COLO829 and mkn45. The subclone confirmed by hierarchical clustering is labeled on the right.

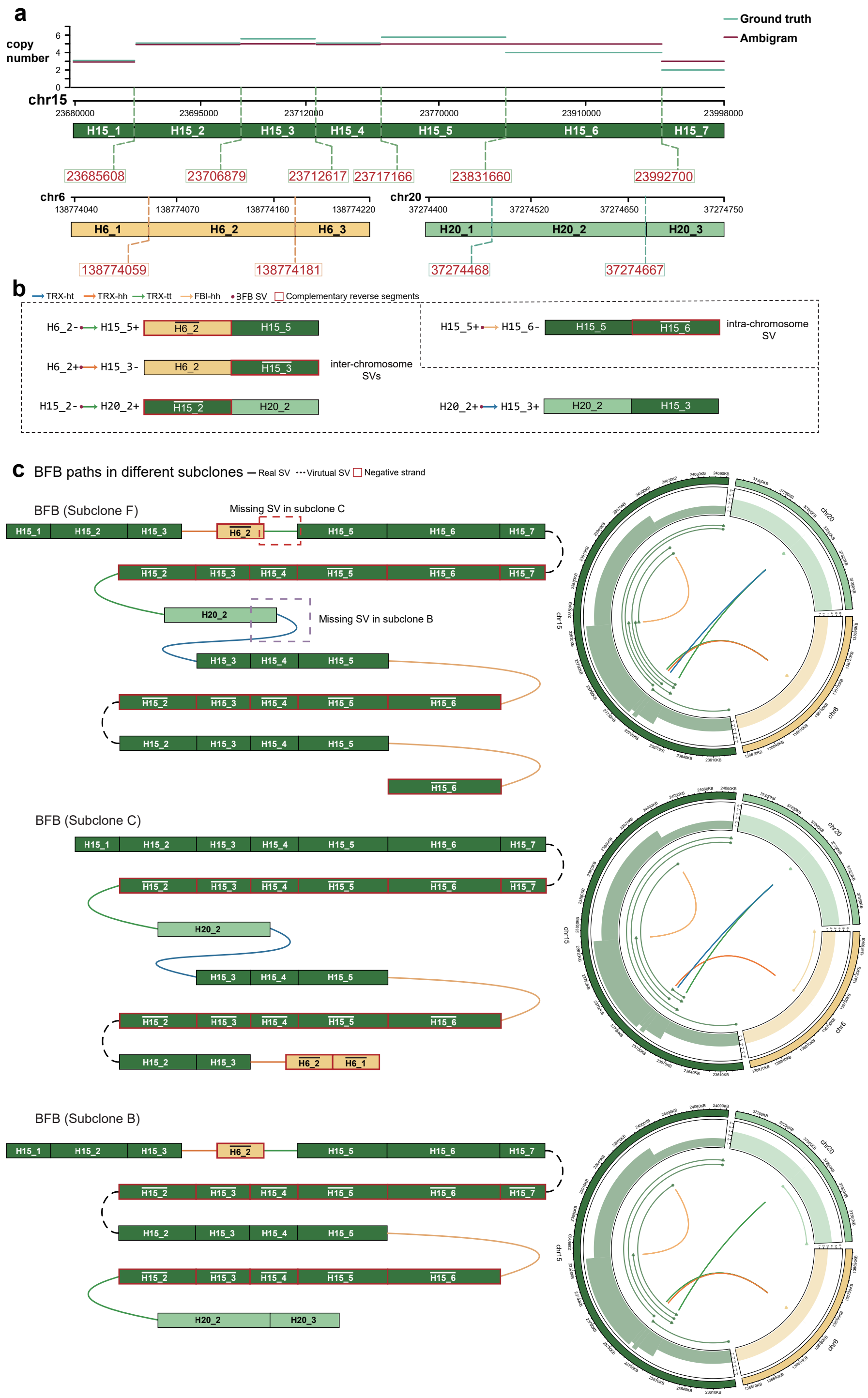

Supplementary Figure 21: Single-cell COLO829 instance 2 - complex BFB involving inter-chromosomal rearrangements on chr15, chr6, and chr20 with heterogeneity among subclones.
(a) The SV breakpoints split the local genome region of chr15 into seven segments and partition chr6 and chr20 into three segments, respectively. The stairstep plot shows the ground truth CNs of segments and the CNs derived by Ambigram. (b) List of SVs and the segments they connected with. The head-to-head (hh) FBI is colored light yellow. The reverse complementary segments have a red border. (c) Ambigram resolved the BFB path for three subclones, F, C, and B. Among these subclones with heterogeneity, subclone F contains complete SV information that can be input into Ambigram to reconstruct a BFB path with all input SVs. Besides, with loss of the translocation on reverse segment $\overline{H6\_2}$ and segment H15_5, the tail of the BFB path in subclone C shortens and ends with segments of chr6 in comparison with the result of subclone F. Similarly, due to loss of the translocation on segments H20_2 and H15_3, Ambigram puts segments of chr20 at the end of the BFB path in subclone B, instead of inserting segment H20_2 into the middle like the path in subclone F. The other three subclones, A, D, and E, have a complete SV set that agrees with bulk sequencing data, and their results are the same as the result of subclone F. The CIRCOS diagrams on the right reflect the CNs, SVs, and the output local genomic maps.

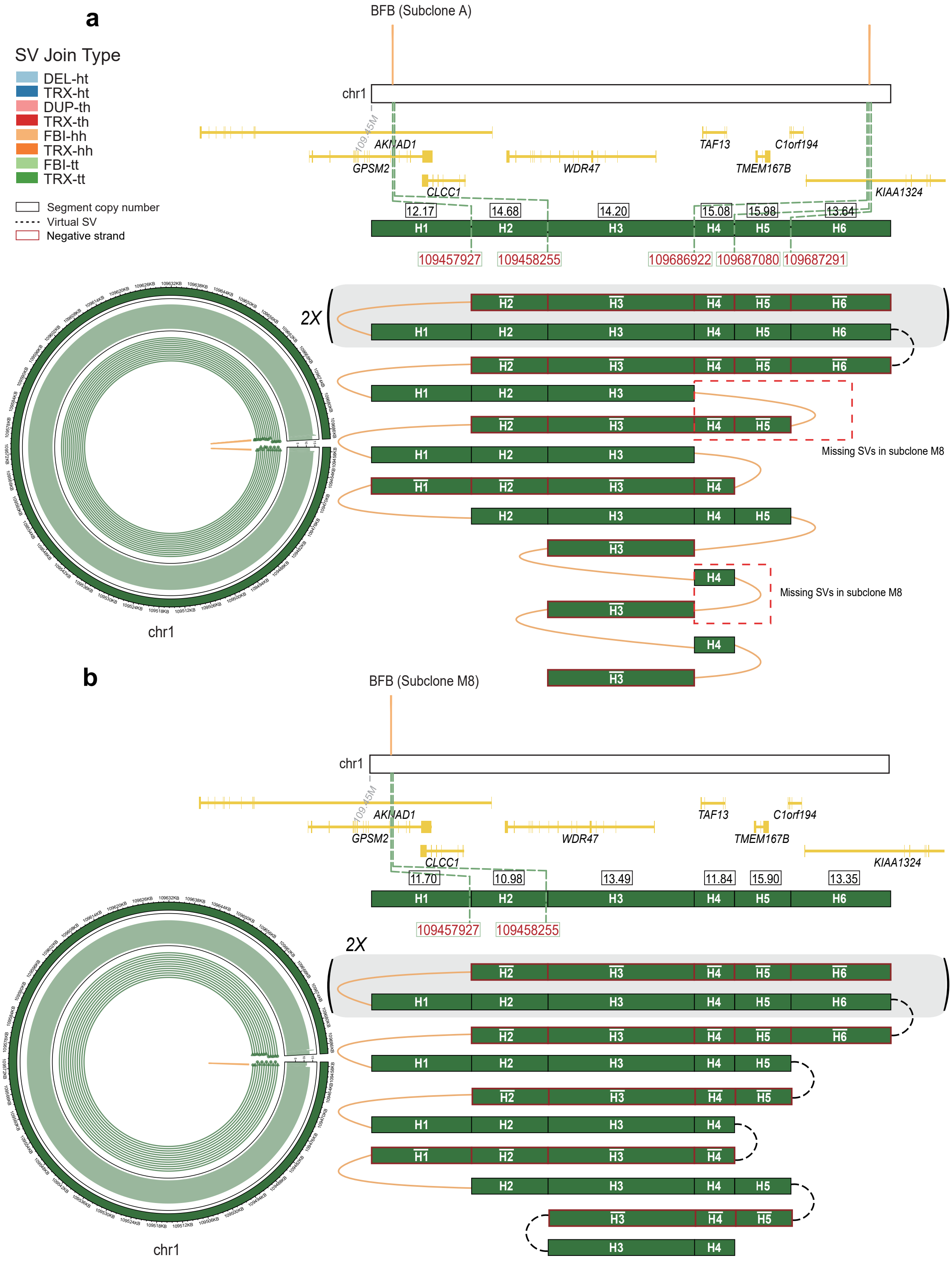

Supplementary Figure 22: Complex BFB event on chr1 for mkn45.
(a) The breakpoints of three FBIs split the local genome region of chr1 in subclone A into six segments. Based on the CN profile of subclone A, Ambigram connects all three SVs in a BFB path derived from nine BFB cycles. The yellow horizontal lines represent gene annotation. The CIRCOS diagram on the left reflects the CNs, SVs, and the output local genomic map. (b) With two FBIs missing in subclone M8, a BFB path similar to that of subclone A is derived from six BFB cycles. Ambigram infers virtual FBIs on segments H3, H4, and H5 of subclone M8 based on integrated FBI and CN information of both subclones. The CIRCOS diagram on the left reflects the CNs, SVs, and the output local genomic map.

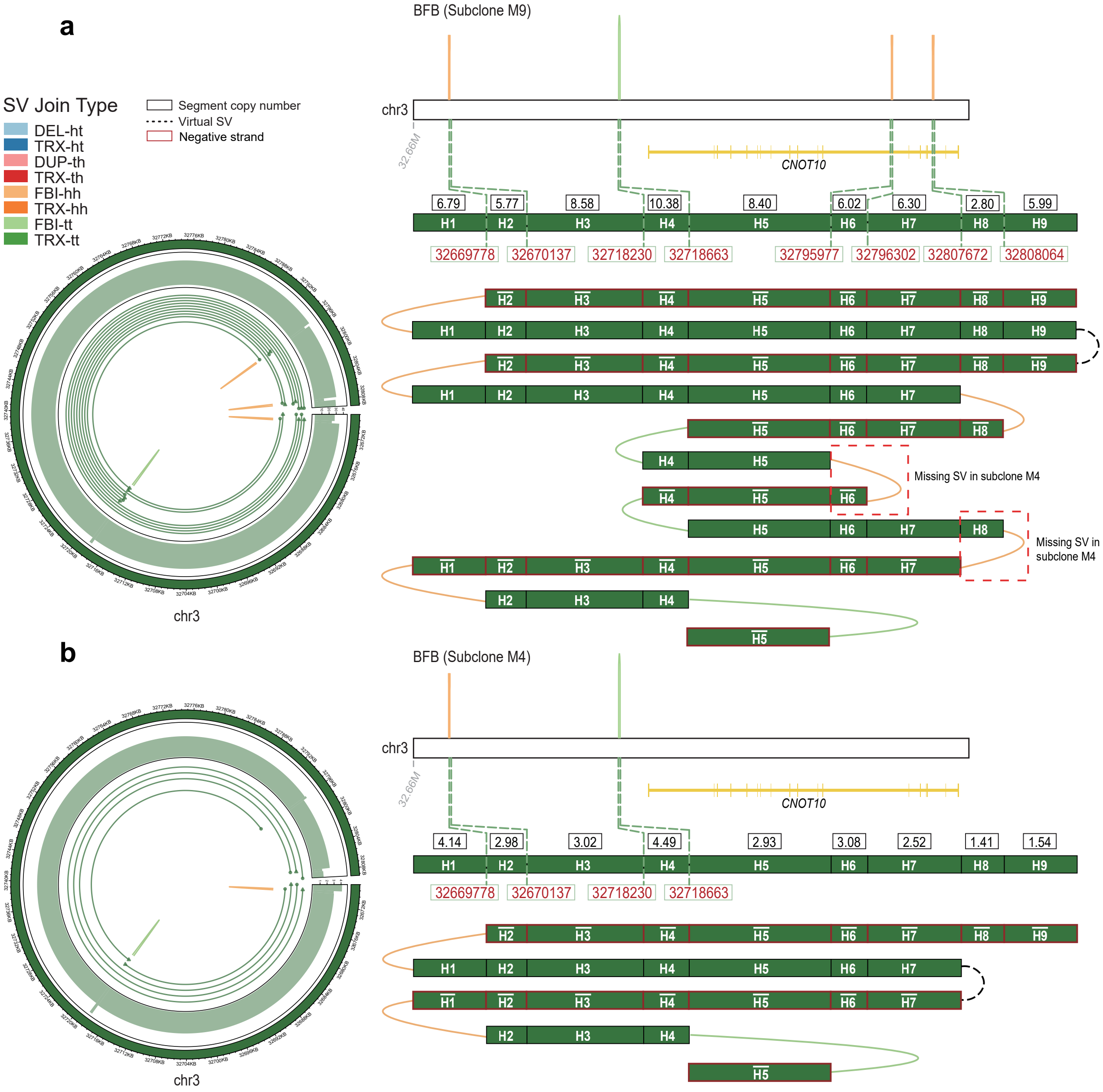

Supplementary Figure 23: Complex BFB event on chr3 for mkn45.
(a) The breakpoints of four FBIs split the local genome region of chr1 in subclone A into nine segments. According to the CN profile, Ambigram resolves a BFB path that includes all four FBIs in subclone M9, which can be explained by seven BFB cycles. The middle layer shows gene CNOT10 involved in this BFB event. The CIRCOS diagram on the left reflects the CNs, SVs, and the output local genomic map. (b) Ambigram resolves another BFB path on the same local genome region of chr3 in subclone M4. Compared with the result of M9, the BFB path of M4, derived from three BFB cycles, only contains two FBIs, with the other two missings. Besides, Ambigram infers a virtual FBI on segment H7 of subclone M4. The CIRCOS diagram on the left reflects the CNs, SVs, and the output local genomic map.

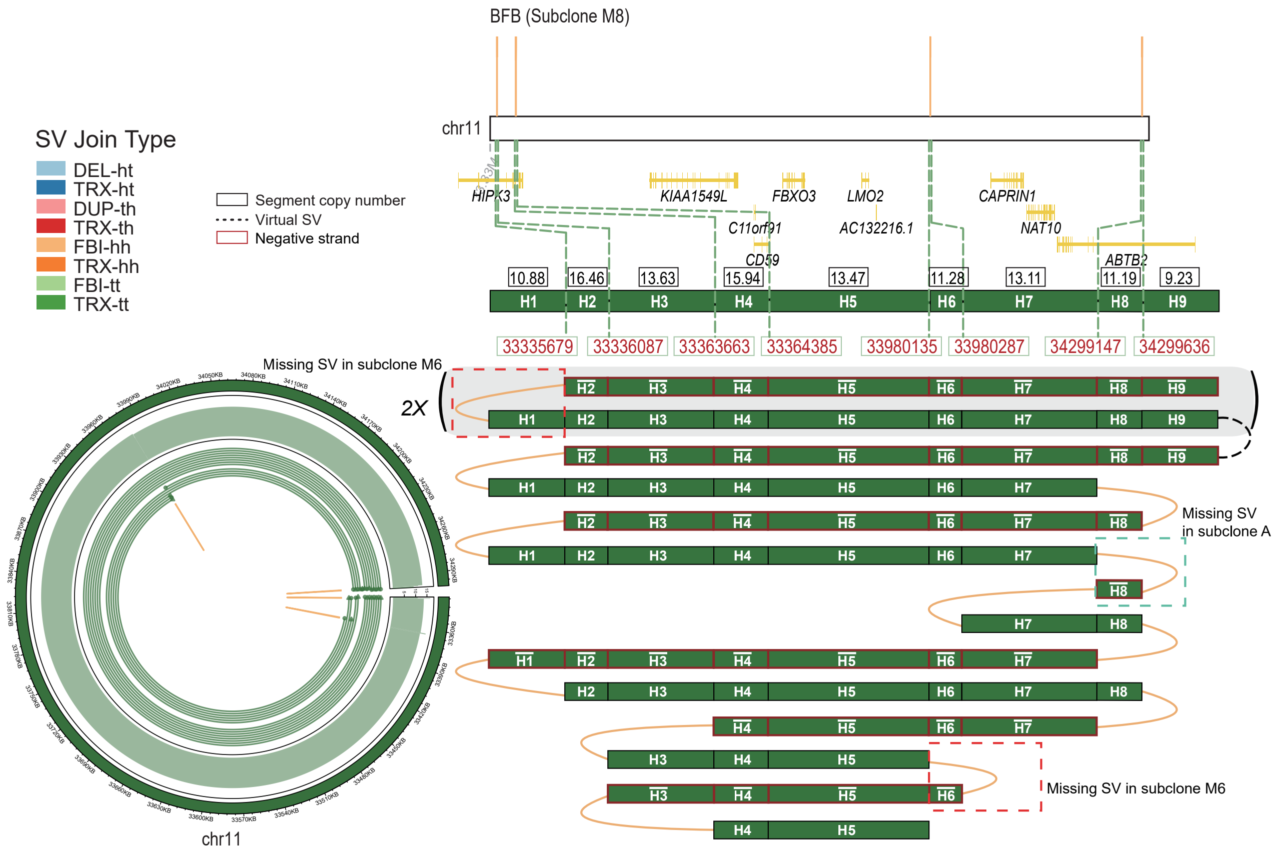

Supplementary Figure 24: Complex BFB event on chr11 in subclone M8 for mkn45.
The local genome region of chr11 in subclone M8 is split into nine segments by four FBIs. With segment CNs ranging from 10 to 16, a BFB path, including all of these FBIs, is constructed through eight BFB cycles. The yellow horizontal lines represent gene annotation. The CIRCOS diagrams on the left reflect the CNs, SVs, and the output local genomic map.

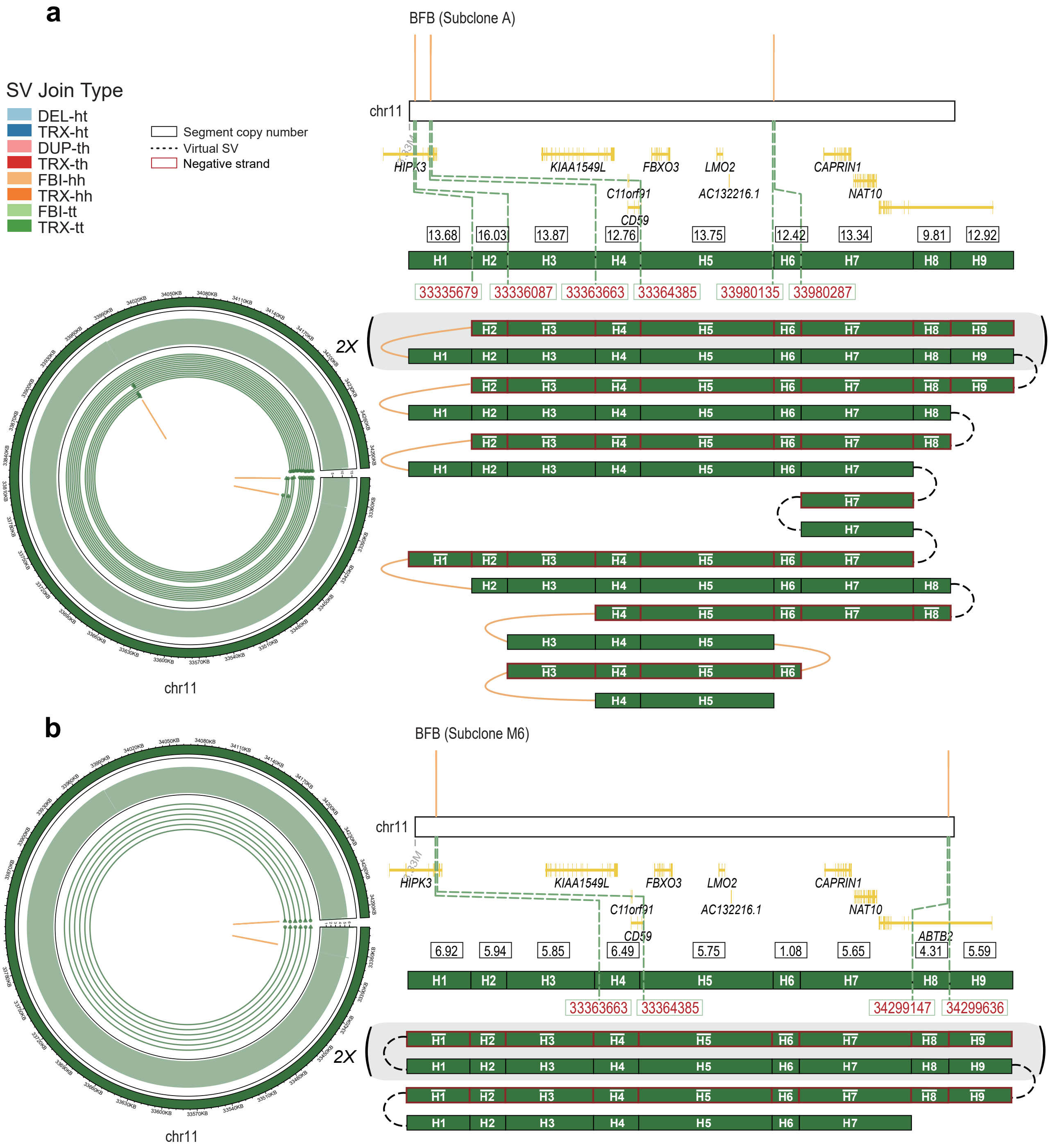

Supplementary Figure 25: Complex BFB event on chr11 in subclones A and M6 for mkn45. (a) With an FBI on segment H7 and reversed segment H8 missing, Ambigram deciphers a local genomic map in subclone A that is similar to that of subclone M8. Besides, Ambigram infers virtual FBIs on segments H7 and H8 of subclone A. This BFB event contains eight BFB cycles. The CIRCOS diagram on the left reflects the CNs, SVs, and the output local genomic map. (b) Since the segment CNs are relatively small, and two FBIs are missing compared with M8, a shorter BFB path consisting of two FBIs is established by three BFB cycles in subclone M6. The CIRCOS diagram on the left reflects the CNs, SVs, and the output local genomic map.

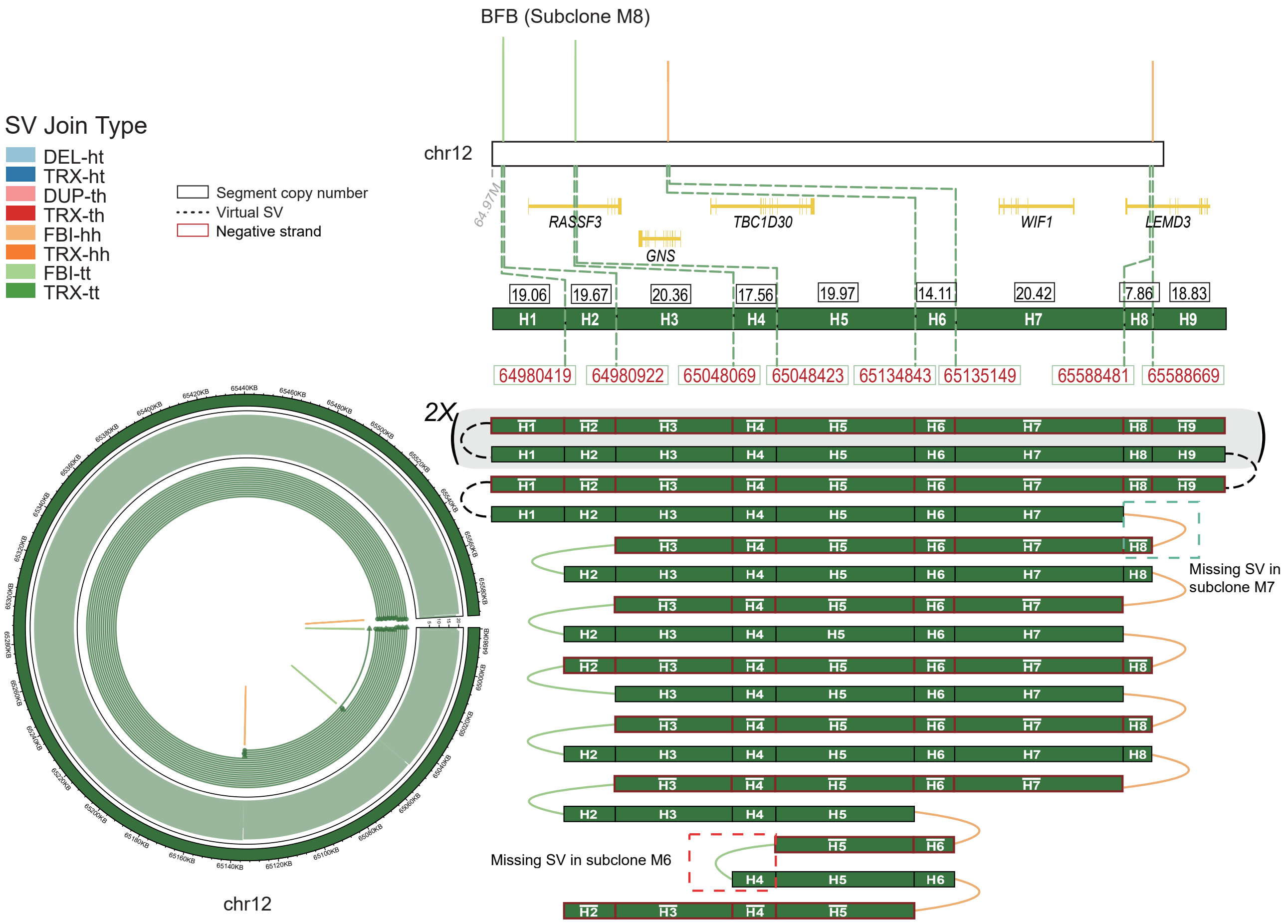

Supplementary Figure 26: Complex BFB event on chr12 in subclone M8 for mkn45. The genome region on chr12 in subclone M8 is split into nine segments by four FBIs. The yellow horizontal lines represent gene annotation. Compared with previous instances, the segment CNs are huge, so Ambigram deciphers a long BFB path containing all the FBIs, with repetitions in the head. Overall, chr12 in subclone M8 undergoes nine BFB cycles. The CIRCOS diagrams on the left reflect the CNs, SVs, and the output local genomic map.

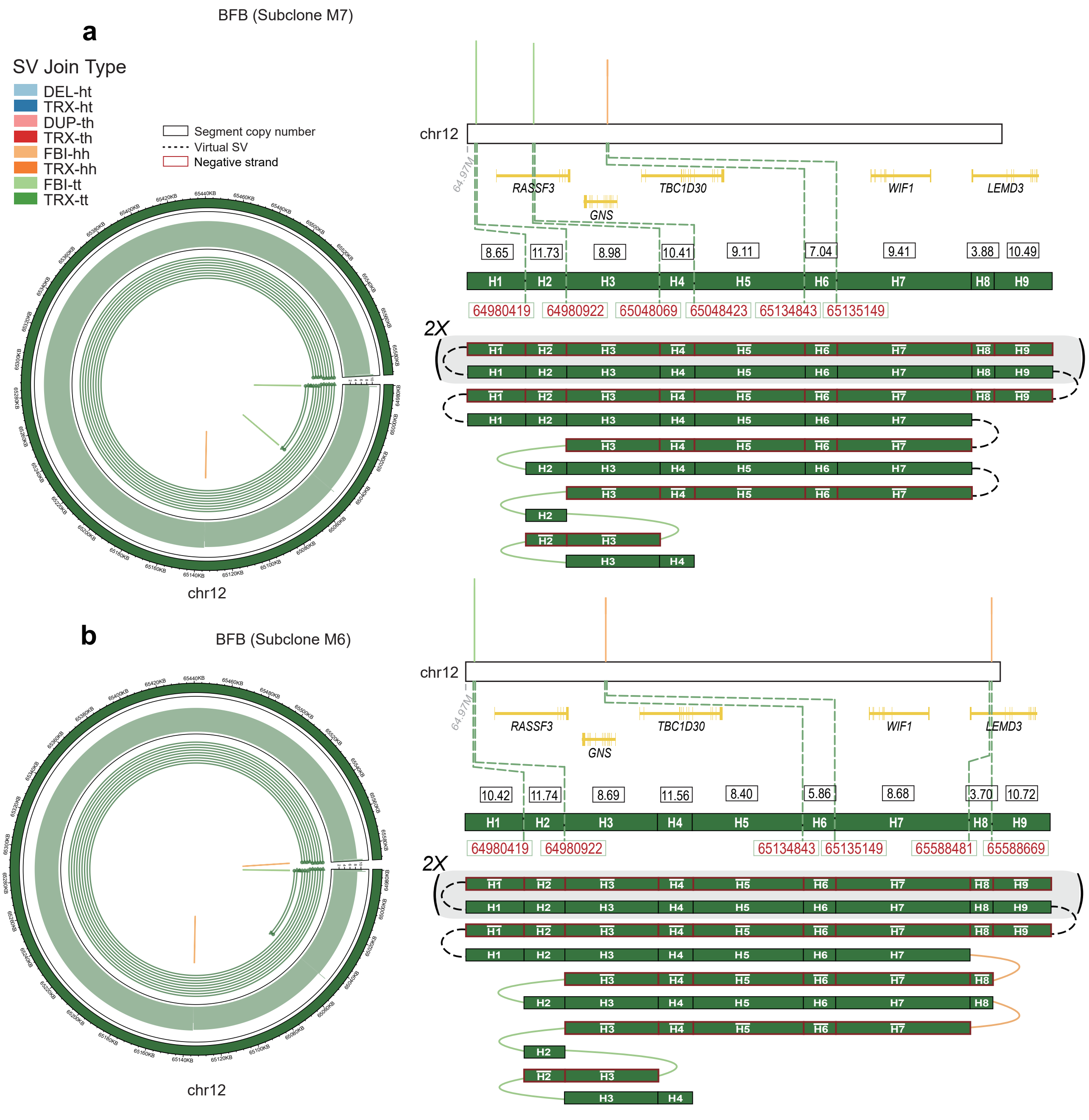

Supplementary Figure 27: Complex BFB event on chr12 in subclones M6 and M7 for mkn45. (a) Compared with M8, although the FBI on segment H7 and reversed segment H8 is missing in subclone M7, a BFB local genomic map resembling M8 is derived from seven BFB cycles. Besides, Ambigram infers a virtual FBI on segment H7 of subclone M7. The CIRCOS diagrams on the left reflect the CNs, SVs, and the output local genomic map. (b) Compared with M8, our algorithm resolves a BFB path in subclone M6 that can be explained by seven BFB cycles. The CIRCOS diagrams on the left reflect the CNs, SVs, and the output local genomic map.

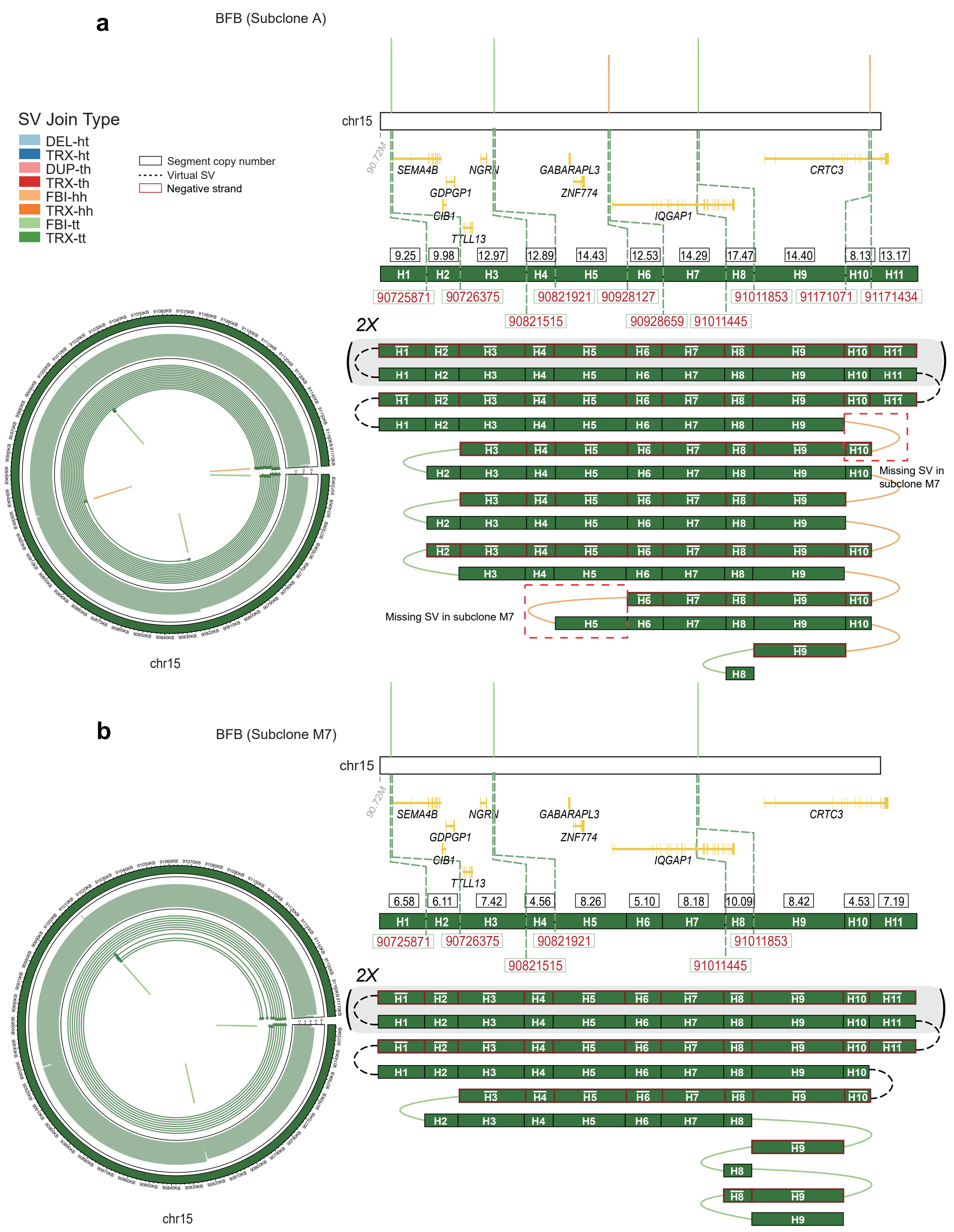

Supplementary Figure 28: Complex BFB event on chr15 for mkn45.
(a) BFB event occurring on chr15 in subclone A involves five FBIs, dividing the genome region into 11 segments. Despite the complicated instance, Ambigram deciphers a BFB path that includes four FBIs based on eight BFB cycles. The yellow horizontal lines represent gene annotation. The CIRCOS diagrams on the left reflect the CNs, SVs, and the output local genomic map. (b) The genome region on chr15 in subclone M7 contains three FBIs and loses two FBIs compared to the same region in subclone M8. A simpler local genomic map is constructed, and subclone M7 undergoes eight BFB cycles. Besides, Ambigram infers a virtual FBI on segment H10 of subclone M7. The CIRCOS diagrams on the left reflect the CNs, SVs, and the output local genomic map.

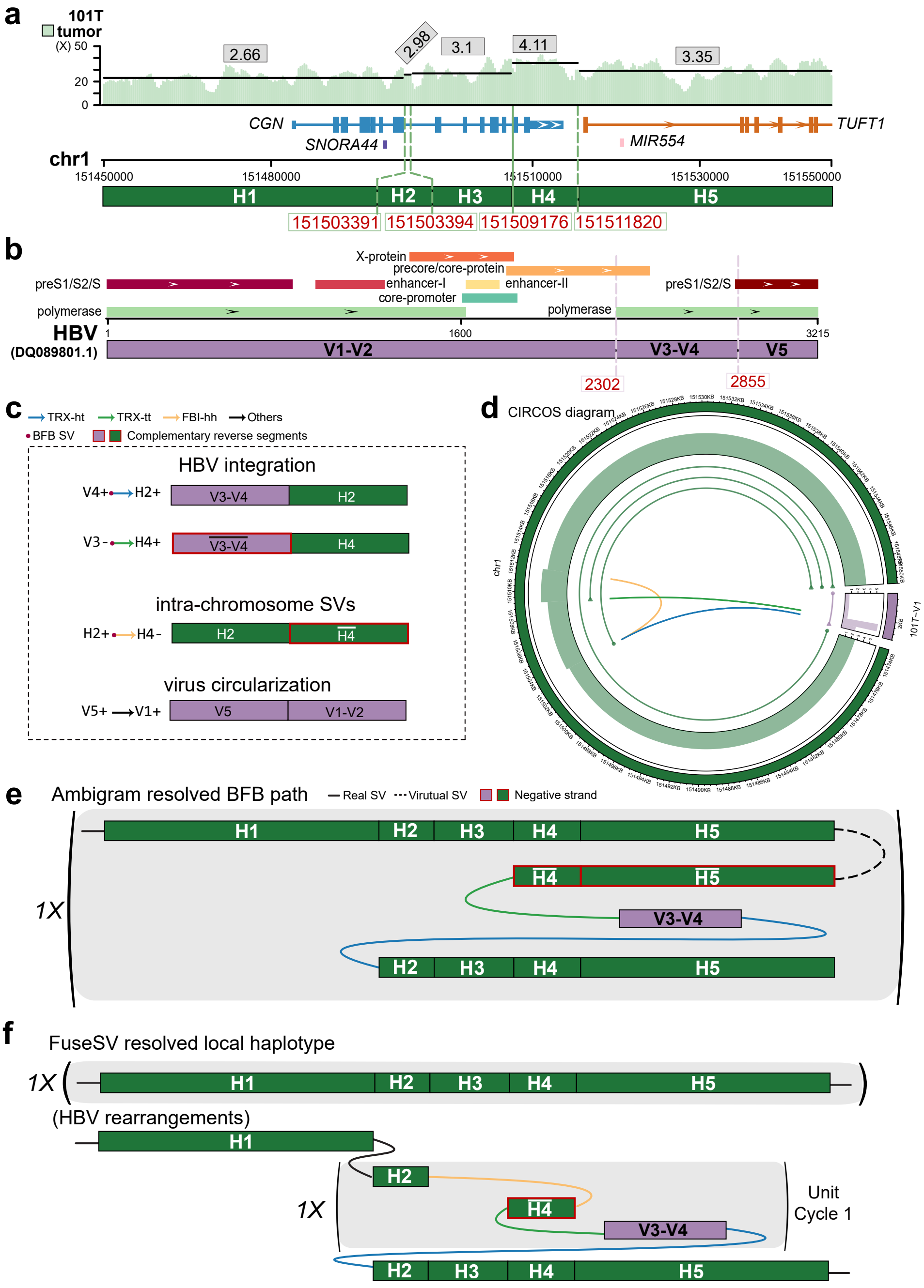

Supplementary Figure 29: Complex BFB involving HBV integration and genes *CGN*, *TUFT1*, *SNORA44*, and *MIR554* on chr1 of HCC 101T [5, 6].
(a) The SV breakpoints split the local genome region into H1, H2, H3, H4, and H5 segments. The light green denotes the read depth of each base pair, and the grey box shows the average CN of segments. The middle layer shows gene annotation. (b) The VIT and SV breakpoints split the virus genome into five segments (V1 - V5). The top layer shows functional annotation. (c) List of VITs, SVs, and the segments connected by them. The head-to-head (hh) and tail-to-tail(tt) FBIs are colored light yellow and light green, respectively. The reverse complementary segments have a red border. (d) CIRCOS diagram of the complex BFB. The outermost track shows the local genome regions involved with the BFB event. The second outermost track illustrates the input region CNs, and the third track indicates the resolved CNs by Ambigram. Besides, the third track shows the resolved BFB paths, in which the circle and triangle points refer to the 5' end and 3' end, respectively. The innermost part represents all the SVs involved with the BFB event. (e) Ambigram infers the BFB event occurs in two stages. In the first stage, chr1 undergoes two BFB cycles. The first BFB cycle occurs when a sister chromatid is replicated, and segment H5 is fused with its reverse complement. Then the second BFB cycle starts when the double-strand breaks off at reverse segment $\overline{H4}$. A duplication is reproduced, and reverse segment $\overline{H4}$ is fused with segment H2. In the second stage, the HBV segments V3 and V4 are inserted into the area between reverse segment $\overline{H4}$ and segment H2 through HBV integration, which indicates the end of this complex BFB event. (f) FuseSV resolved local genomic map. Note that (a-c) and (e) are modified from the original paper of FuseSV [6].

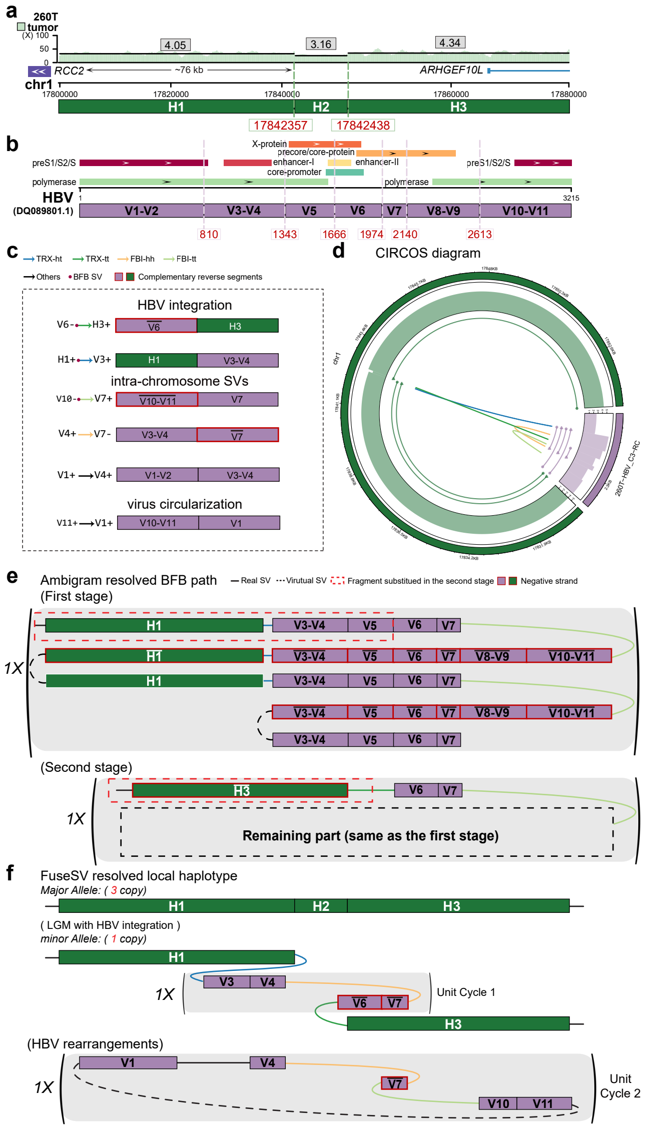

Supplementary Figure 30: Complex BFB involving HBV integration and genes *RCC2* and *ARHGEF10L* on chr1 of HCC 260T [5, 6].

(a) The VIT breakpoints split the local genome region into H1, H2, and H3 segments. The light green denotes the read depth of each base pair, and the grey box shows the average CN of segments. The middle layer shows gene annotation. (b) The VIT and SV breakpoints split the virus genome into 11 segments (V1 - V11). The top layer shows functional annotation. (c) List of VITs, SVs, and the segments they connected with. The head-to-head (hh) and tail-to-tail(tt) FBIs are colored light orange and light green, respectively. The reverse complementary segments have a red border. (d) CIRCOS diagram of the complex BFB. The outermost track shows the local genome regions involved with the BFB event. The second outermost track illustrates the input region CNs, and the third track indicates the resolved CNs by Ambigram. Besides, the third track shows the resolved BFB paths, in which the circle and triangle points refer to the 5' end and 3' end, respectively. The innermost part represents all the SVs involved with the BFB event. (e) Ambigram resolved the BFB path. First, segment H1 is connected to segment V3 through HBV integration. Then the HBV-integrated genome sequence undergoes three BFB cycles. The first cycle starts when the breakage happens at segment V7. A sister chromatid is reproduced, and segment V7 is fused with reverse segment $\overline{V10}$. Then the second BFB cycle occurs when a sister chromatid is replicated, and segment H1 is fused with its reverse complement. Finally, the third BFB cycle fuses segments V3 and $\overline{V3}$. In the second stage, reverse segment $\overline{H3}$ replaces the head of the BFB path, which indicates the end of this complex BFB event. (f) FuseSV resolved local genomic map. In sample 260 T, the HBV integrated upstream of the *RCC2* gene on chr1 (triploid, minor CN=1). The local genomic map on chr1 contains virus inversion V4+ to V7 connected HBV DNA segments, which induced the deletion of the H2 host segment on the minor allele. The viral enhancer I/II and core promoter in the local genomic map might promote the expression of oncogene *RCC2*. Note that (a-c) and (e) are modified from FuseSV's original paper [6].

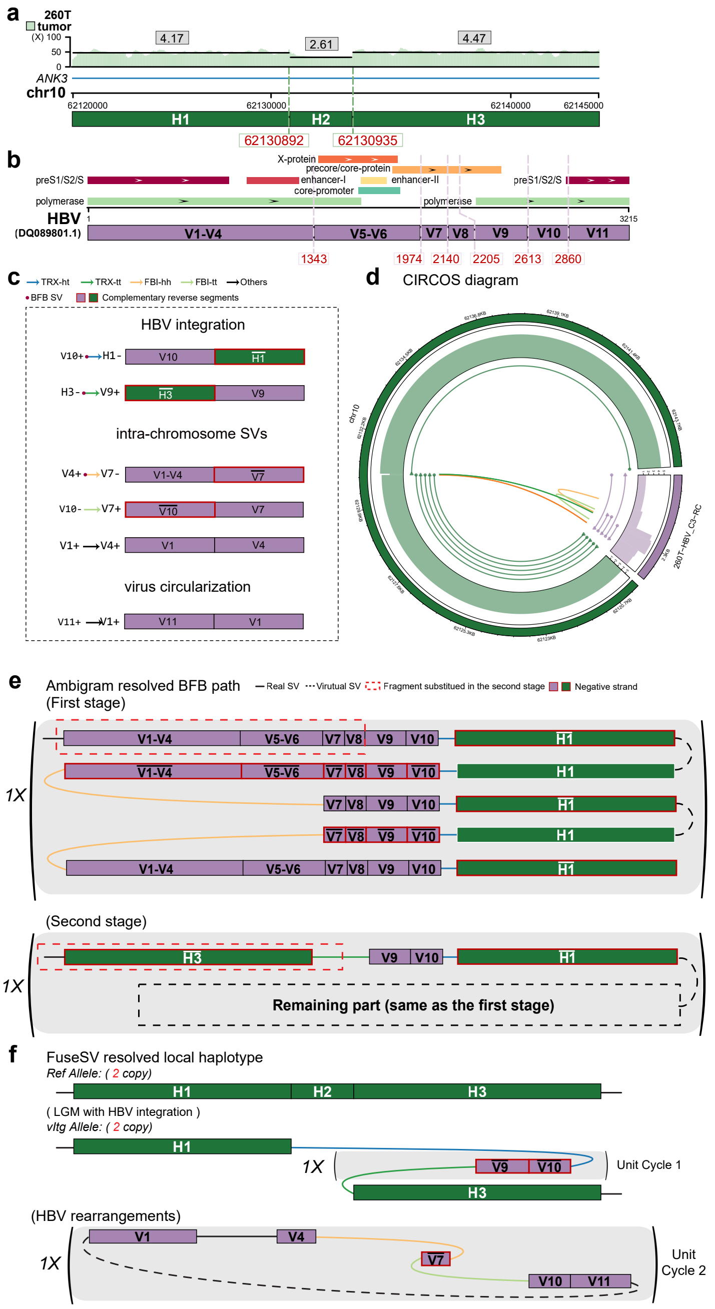

Supplementary Figure 31: Complex BFB involving HBV integration and gene *ANK3* on chr10 of HCC 260T [5, 6].
(a) The VIT breakpoints split the local genome region into H1, H2, and H3 segments. The light green denotes the read depth of each base pair, and the grey box shows the average CN of segments. The middle layer shows gene annotation. (b) The VIT and SV breakpoints split the virus genome into 11 segments (V1 - V11). The top layer shows functional annotation. (c) List of VITs, SVs, and the segments they connected with. The head-to-head (hh) and tail-to-tail(tt) FBIs are colored light orange and light green, respectively. The reverse complementary segments have a red border. (d) CIRCOS diagram of the complex BFB. The outermost track shows the local genome regions involved with the BFB event. The second outermost track illustrates the input region CNs, and the third track indicates the resolved CNs by Ambigram. Besides, the third track shows the resolved BFB paths, in which the circle and triangle points refer to the 5' end and 3' end, respectively. The innermost part represents all the SVs involved with the BFB event. (e) Ambigram resolved the BFB path. Segment V10 is linked to reverse segment $\overline{H1}$ on chr10 through HBV integration, and then the HBV-integrated genome sequence encounters three BFB cycles. Firstly, a sister chromatid is replicated, and reverse segment $\overline{H1}$ is fused with its reverse complement. Besides, the second BFB cycle occurs on segments V4 and $\overline{V7}$. Finally, the third BFB cycle fuses reverse segment $\overline{H1}$ and its reverse complement. (f) FuseSV resolved local genomic map. The HBV integrated at the *ANK3* gene on chr10 (tetraploid, minor CN=2). Short inversed HBV DNA segments V9-V10 were included in the chr10 local genomic map and substituted the H2 host segment (intron of ANK3) in two allele copies. Note that (a-c) and (e) are modified from FuseSV's original paper [6].

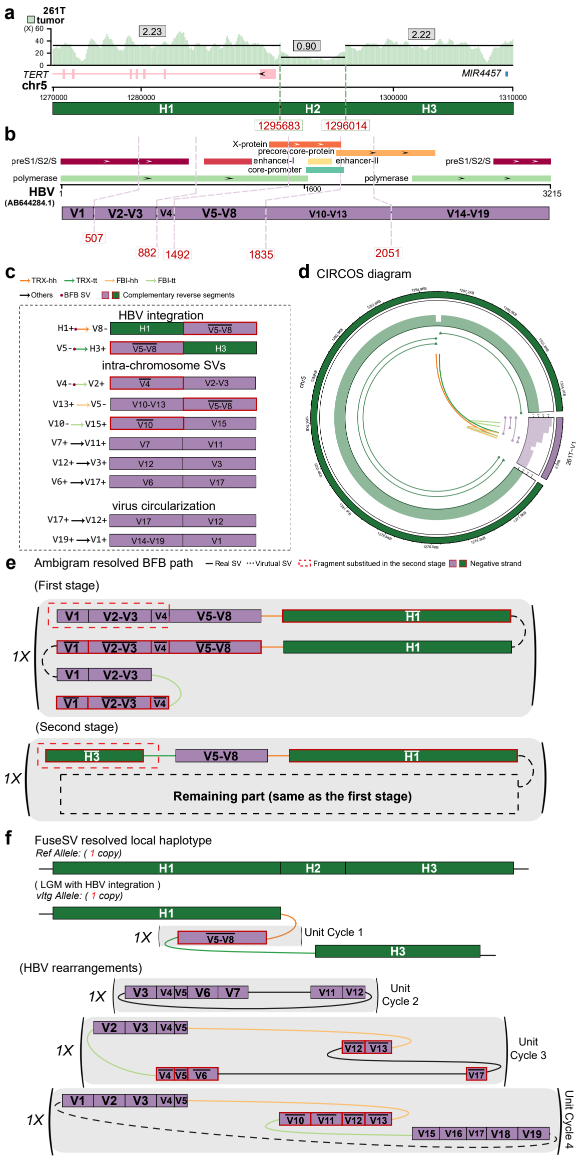

Supplementary Figure 32: Complex BFB involving HBV integration and genes *TERT* and *MIR4457* on chr5 of HCC 261T [5, 6].
(a) The VIT breakpoints split the local genome region into H1, H2, and H3 segments. The light green denotes the read depth of each base pair, and the grey box shows the average CN of segments. The middle layer shows gene annotation. (b) The VIT and SV breakpoints split the virus genome into 19 segments (V1 - V19). The top layer shows functional annotation. (c) List of VITs, SVs, and the segments they connected with. The head-to-head (hh) and tail-to-tail(tt) FBIs are colored light orange and light green, respectively. The reverse complementary segments have a red border. (d) CIRCOS diagram of the complex BFB. The outermost track shows the local genome regions involved with the BFB event. The second outermost track illustrates the input region CNs, and the third track indicates the resolved CNs by Ambigram. Besides, the third track shows the resolved BFB paths, in which the circle and triangle points refer to the 5' end and 3' end, respectively. The innermost part represents all the SVs involved with the BFB event. (e) Ambigram resolved the BFB path. This complex BFB event consists of two stages. In the first stage, segment V8 is integrated with reverse segment $\overline{H1}$ on chr5, and the HBV-integration genome sequence undergoes three BFB cycles. The first two cycles occur when chromosomal duplication happens, and reverse segments $\overline{H1}$ and $\overline{V1}$ are fused with their reverse complements, respectively. Then the third BFB cycle starts when the breakage occurs at segment V2, and a sister chromatid is reproduced, followed by the fusion between segment V2 and reverse segment $\overline{V4}$ on the chromatid duplication. In the second stage, another HBV integration happens between reverse segment $\overline{H3}$ and segment V5, replacing the head of the BFB path. (f) FuseSV detects the local genomic map of HBV integration at the heterozygous diploid chr5 of sample 261T, which consists of one copy of the normal allele (h1-H3), and one copy of the integration allele. Four short HBV segments (V5-V8) inversely integrate upstream of oncogene *TERT*, with one copy deletion of human segment H2. The virus enhancer-II and core promoter located in the inserted HBV segments may explain the up-regulated expression of *TERT* in sample 261T. Note that (a-c) and (e) are modified from FuseSV's original paper [6].

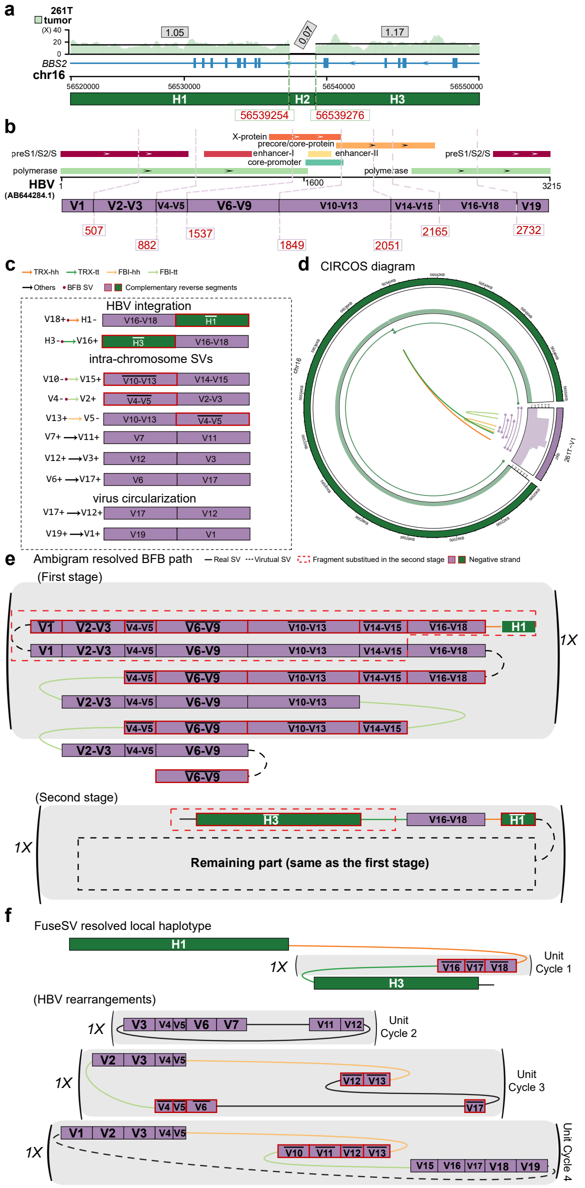

Supplementary Figure 33: Complex BFB involving HBV integration and gene *BBS2* on chr16 of HCC 261T [5, 6].

(a) The VIT breakpoints split the local genome region into H1, H2, and H3 segments. The light green denotes the read depth of each base pair, and the grey box shows the average CN of segments. The middle layer shows gene annotation. (b) The VIT and SV breakpoints split the virus genome into 19 segments (V1 - V19). The top layer shows functional annotation. (c) List of VITs, SVs, and the segments they connected with. The head-to-head (hh) and tail-to-tail(tt) FBIs are colored light orange and light green, respectively. The reverse complementary segments have a red border. (d) CIRCOS diagram of the complex BFB. The outermost track shows the local genome regions involved with the BFB event. The second outermost track illustrates the input region CNs, and the third track indicates the resolved CNs by Ambigram. Besides, the third track shows the resolved BFB paths, in which the circle and triangle points refer to the 5' end and 3' end, respectively. The innermost part represents all the SVs involved with the BFB event. (e) Ambigram resolved the BFB path. This complex BFB event consists of two stages. In the first stage, reverse segment $\overline{V18}$ is integrated with segment H1 on chr16, and the HBV-integration local genomic map undergoes five BFB cycles. The first two cycles occur when chromosomal duplication happens, and segments $\overline{V1}$ and V18 are fused with their reverse complements, respectively. Then the third BFB cycle starts when the breakage occurs at reverse segment $\overline{V4}$. A sister chromatid is reproduced, followed by the fusion between segments $\overline{V4}$ and V2 on the chromatid duplication. Moreover, another breakage occurs at segment V10, and a sister chromatid is replicated. Then segments V10 and $\overline{V15}$ are fused. Finally, the last BFB cycle fuses segments V9 and $\overline{V9}$ through a virtual FBI inferred by Ambigram. In the second stage, another HBV integration happens between reverse segment $\overline{H3}$ and segment V16, replacing the head of the BFB path. (f) FuseSV claims the local genomic map of virus integration on chr16 is the deletion of the H2 segment by inserting short linear HBV segments. The left viral SVs might form possible circular HBV ecDNAs with deletion, tandem duplication, and inversion. Note that (a-c) and (e) are adapted from FuseSV's original paper [6].

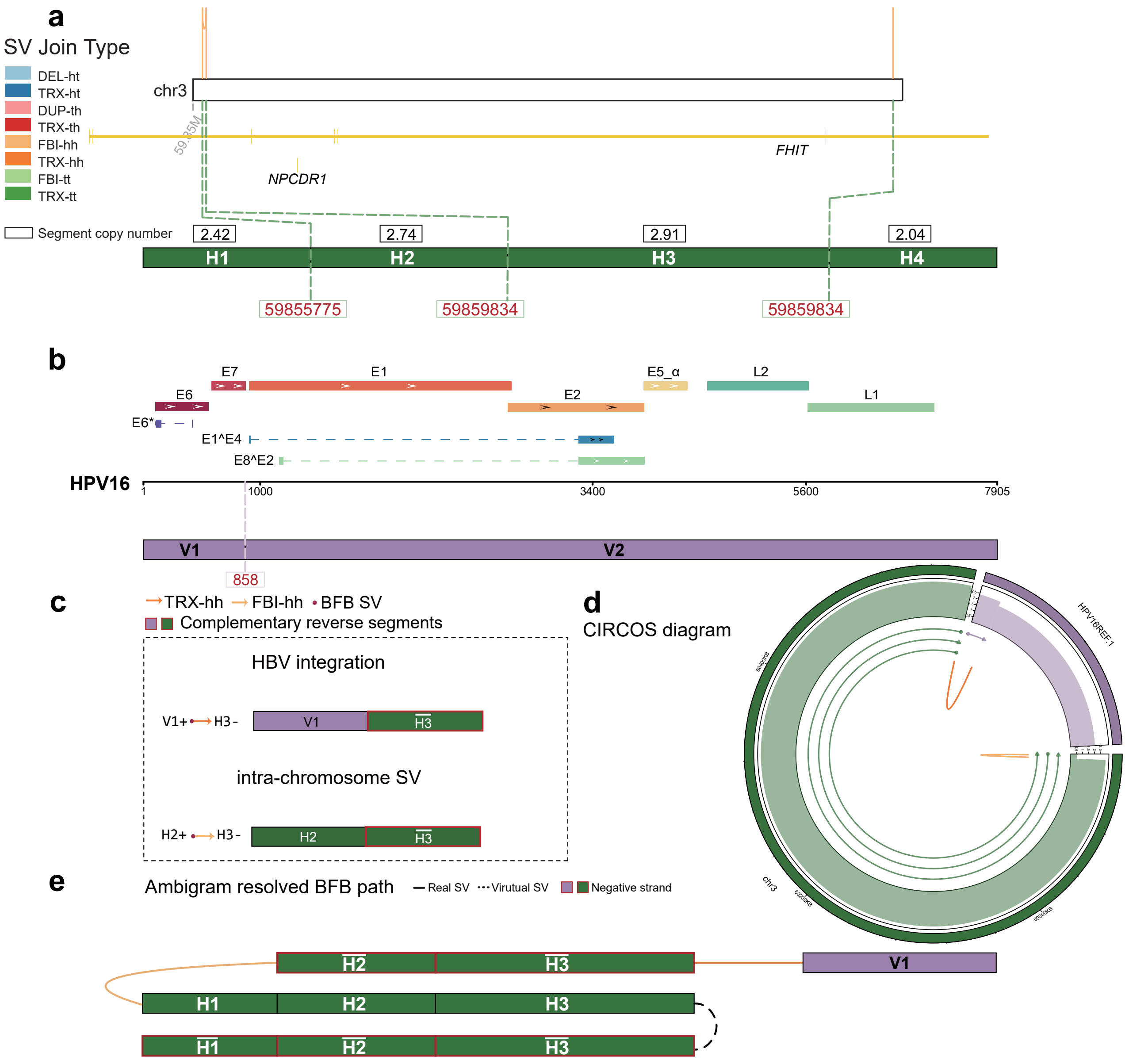

Supplementary Figure 34: Complex BFB involving HPV integration and gene *FHIT* on chr3 of HELA cell line.

(a) The VIT and SV breakpoints split the local genome region into H1, H2, H3, and H4 segments. The vertical lines above show the positions of VIT and SV on chr3, and the middle layer shows gene annotation. The black box shows the average CN of segments. (b) The VIT breakpoint splits the virus genome into V1 and V2 segments. The top layer shows functional annotation. (c) List of VITs, SVs, and the segments they connected with. The head-to-head (hh) and tail-to-tail (tt) FBIs are colored light orange and light green, respectively. The reverse complementary segments have a red border. (d) CIRCOS diagram of the complex BFB. The outermost track shows the local genome regions involved with the BFB event. The second outermost track illustrates the input region CNs, and the third track indicates the resolved CNs by Ambigram. Besides, the third track shows the resolved BFB paths, in which the circle and triangle points refer to the 5' end and 3' end, respectively. The innermost part represents all the SVs involved with the BFB event. (e) Ambigram resolved the BFB path. In the complex BFB event, segment V1 is integrated with reverse segment $\overline{H3}$ on chr3, and the HBV-integration local genomic map undergoes two BFB cycle. The first BFB cycle occurs when the breakage happens at reverse segment $\overline{H2}$, and then segments $\overline{H2}$ and H1 are fused. The second BFB cycle fuses segments H3 and $\overline{H3}$ through a virtual FBI.

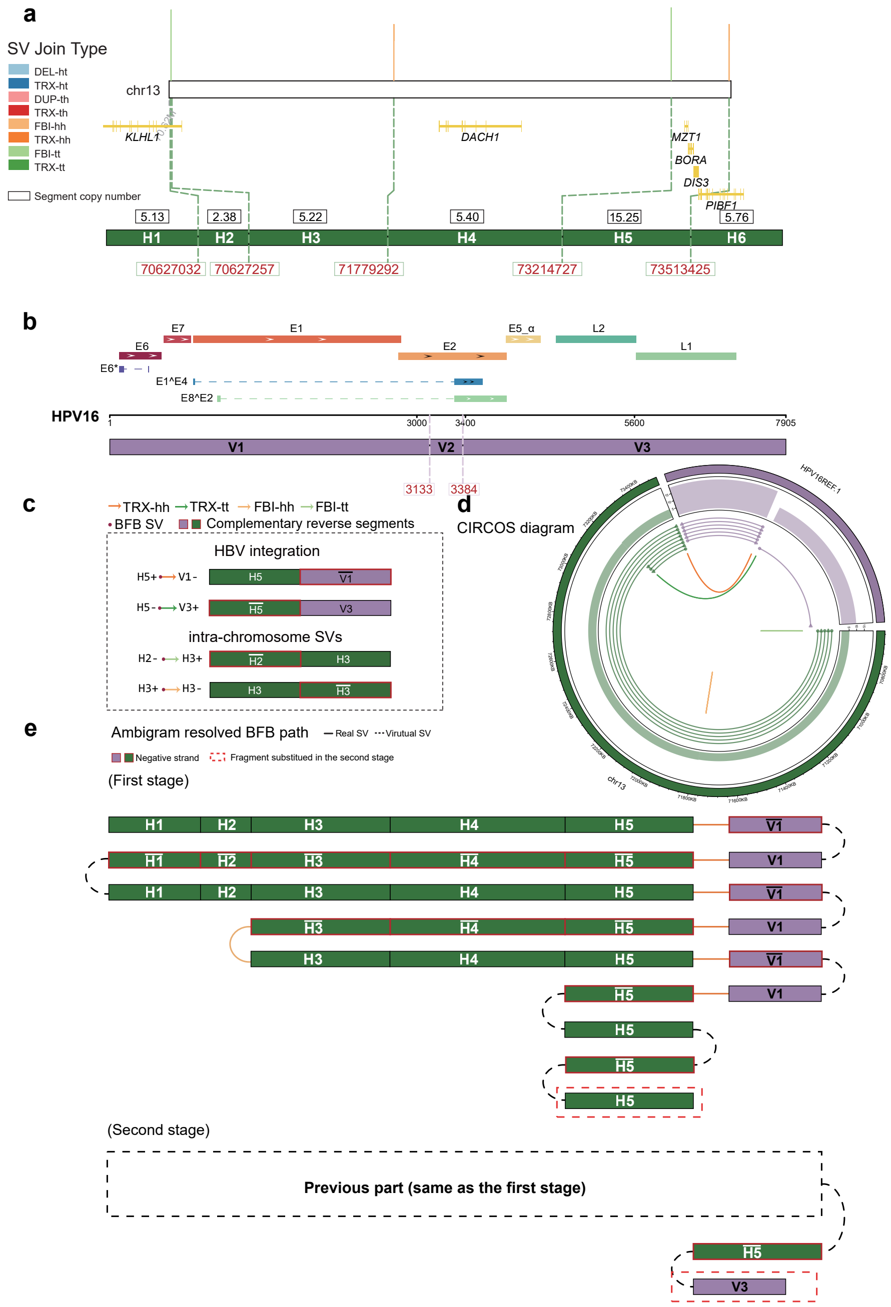

Supplementary Figure 35: Complex BFB involving HPV integration on chr13 of SIHA cell line. (a) The VIT and SV breakpoints split the local genome region into six segments. The vertical lines above show the positions of VITs and SVs on chr13, and the middle layer shows gene annotation. The black box shows the average CN of segments. (b) The VIT breakpoints split the virus genome into V1, V2, and V2 segments. The top layer shows functional annotation. (c) List of VITs, SVs, and the segments they connected with. The head-to-head (hh) translocation and FBIs are colored orange and light orange, respectively. The reverse complementary segments have a red border. (d) CIRCOS diagram of the complex BFB. The outermost track shows the local genome regions involved with the BFB event. The second outermost track illustrates the input region CNs, and the third track indicates the resolved CNs by Ambigram. Besides, the third track shows the resolved BFB paths, in which the circle and triangle points refer to the 5' end and 3' end, respectively. The innermost part represents all the SVs involved with the BFB event. (e) Ambigram resolved the BFB path. This complex BFB event consists of two stages. In the first stage, segment H5 on chr13 is integrated with reverse segment $\overline{V1}$, and the HBV integration undergoes five BFB cycles. The first two cycles occur when chromosomal duplication happens, and reverse segments $\overline{V1}$ and $\overline{H1}$ are fused with their reverse complements, respectively. Then the third BFB cycle starts when the breakage occurs at reverse segment $\overline{H3}$. A sister chromatid is reproduced, followed by the fusion between reverse segment $\overline{H3}$ and segment H3 on the chromatid duplication. Furthermore, the last two cycles fuse segments $\overline{H5}$ and H5 with their reverse complements, respectively, indicating the end of the first stage. In the second stage, another HBV integration happens between reverse segment $\overline{H5}$ and segment V3, replacing the tail of the BFB path.

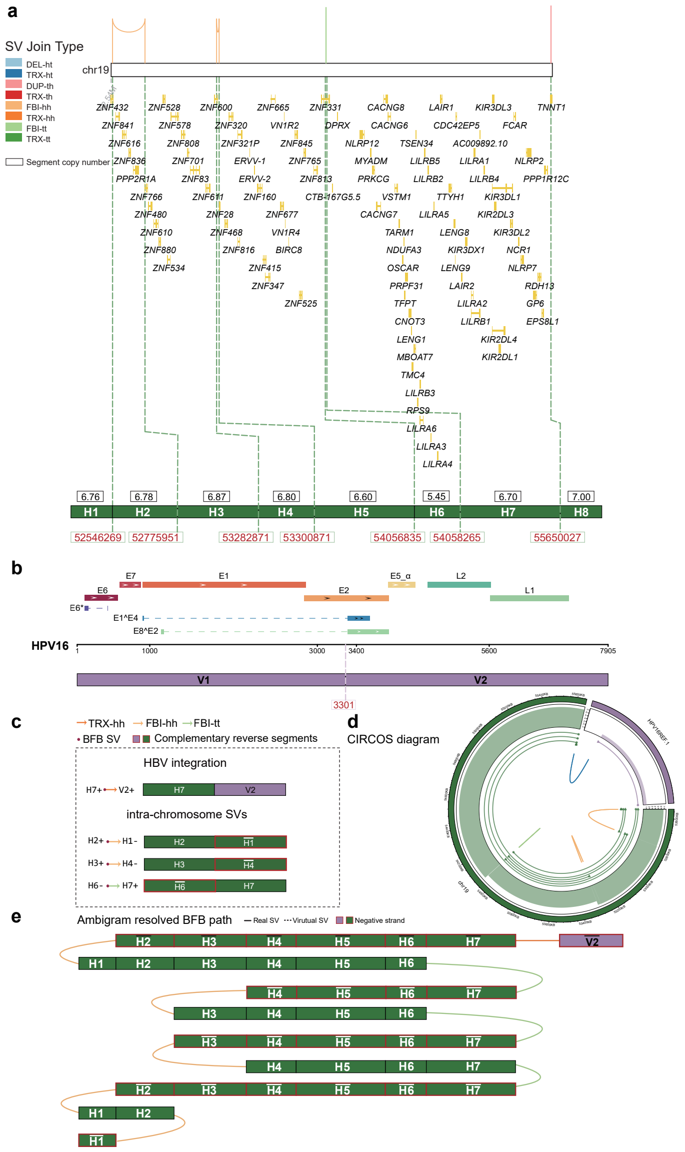

Supplementary Figure 36: Complex BFB involving HPV integration on chr19 of cervical cancer sample CRR046045.
(a) The VIT and SV breakpoints split the local genome region into eight segments. The vertical lines above show the positions of VIT and SVs on chr19, and the middle layer shows gene annotation. The black box shows the average CN of segments. (b) The VIT breakpoint splits the virus genome into V1 and V2 segments. The top layer shows functional annotation. (c) List of VIT, SVs, and the segments they connected to. The head-to-head (hh) and tail-to-tail (tt) FBIs are colored light orange and light green, respectively. The reverse complementary segments have a red border. (d) CIRCOS diagram of the complex BFB. The outermost track shows the local genome regions involved with the BFB event. The second outermost track illustrates the input region CNs, and the third track indicates the resolved CNs by Ambigram. Besides, the third track shows the resolved BFB paths, in which the circle and triangle points refer to the 5' end and 3' end, respectively. The innermost part represents all the SVs involved with the BFB event. (e) Ambigram resolved the BFB path. In the complex BFB event, reverse segment $\overline{H7}$ on chr19 is integrated with reverse segment $\overline{V2}$, and the HBV-integration local genomic map undergoes six BFB cycles. The first BFB cycle occurs when the breakage happens at reverse segment $\overline{H2}$, which is fused with segment H1 on the sister chromatid. Then the second BFB cycle starts when breakage happens on segment H6 and the segment is fused with reverse segment $\overline{H7}$ on the sister chromatid derived from chromosomal duplication. The third BFB cycle starts when another breakage occurs at reverse segment $\overline{H4}$. A sister chromatid is reproduced, followed by the fusion between reverse segment $\overline{H4}$ and segment H3 on the chromatid duplication. Moreover, the fourth BFB cycle occurs when another breakage happens at segment H6. Then segment H6 is fused with reverse segment $\overline{H7}$ on the sister chromatid. Furthermore, the fifth cycle is similar to the first cycle, leading to reverse segment $\overline{H2}$ being fused with segment H1 on the sister chromatid. Finally, another breakage occurs at segment H2, and a sister chromatid is replicated. The final fusion between segments H2 and $\overline{H1}$ contributes to a stable state.

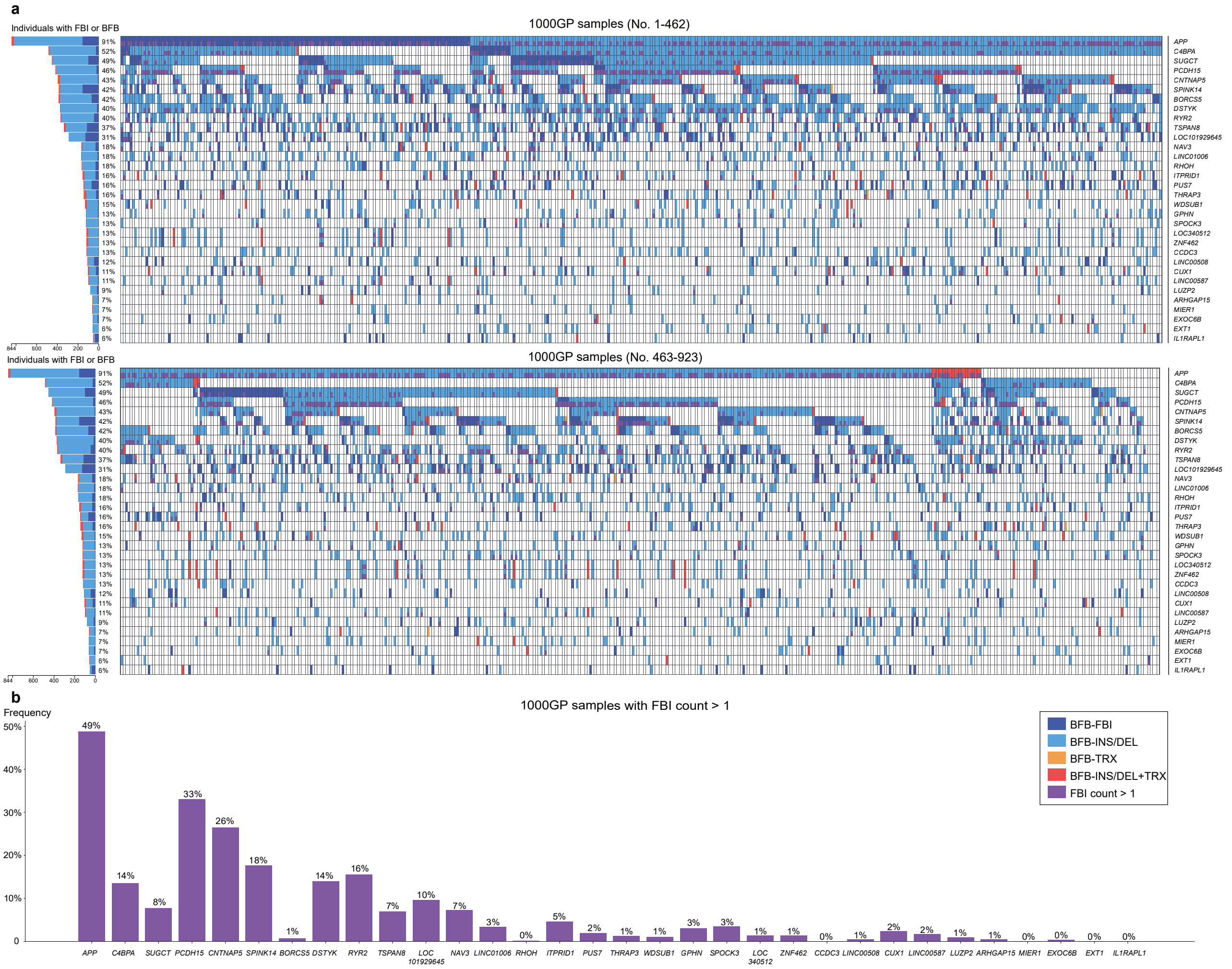

Supplementary Figure 37: BFB analysis in 923 healthy genomes from 1000GP.
(a) The recurrent FBI landscape in 923 healthy genomes from 1000GP. The landscape is plotted by `https://bio.oviz.org/demo-project/analyses/landscape` [18]. (b) The frequency of genes carrying BFB event (FBI count > 1) in 923 healthy genomes from 1000GP.

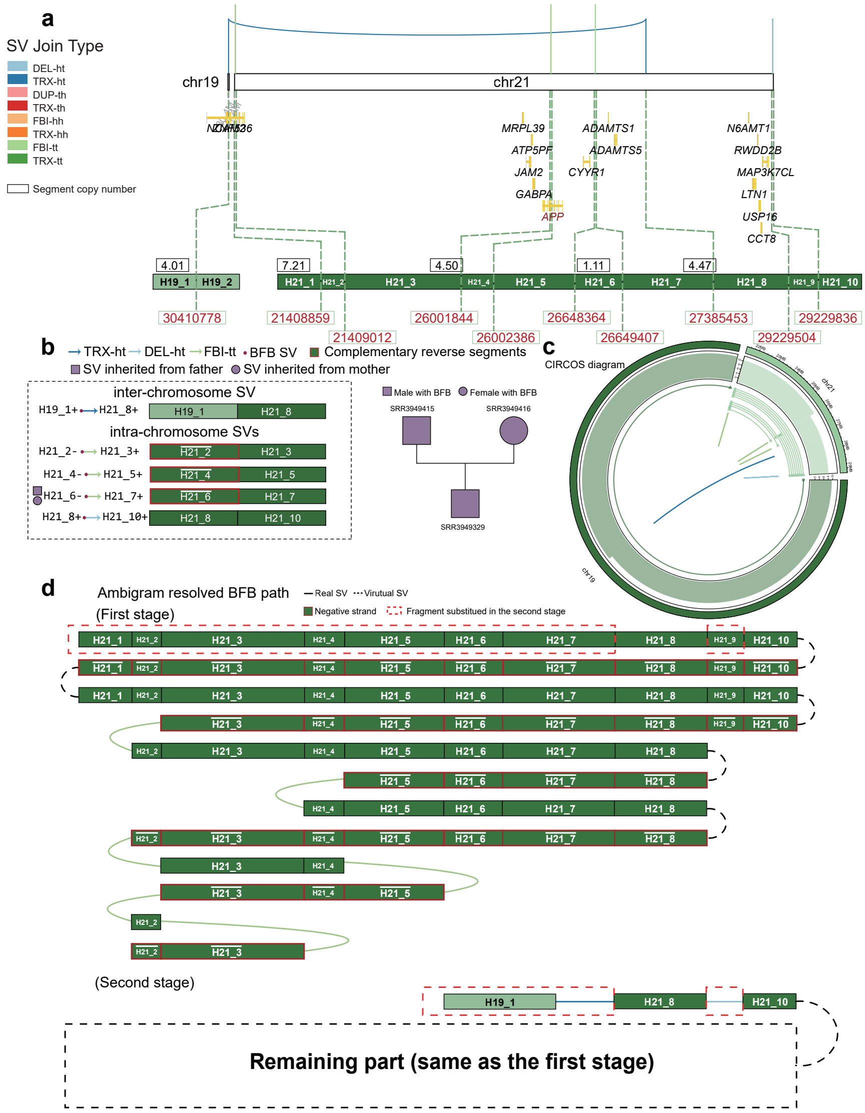

Supplementary Figure 38: Recurrent complex BFB involving chr21 and chr19 of CHD sample SRR3949329. (a) The SV breakpoints split the local genome regions of chr21 and chr19 into ten and two segments, respectively. The vertical lines above show the positions of SVs, and the middle layer shows gene annotation. The black box shows the average CN of segments. (b) List of SVs and the segments connected by them. The head-to-tail (ht) translocation, head-to-tail (ht) deletion, and tail-to-tail (tt) FBIs are colored blue, light blue, and light orange, respectively. The reverse complementary segments have a red border. The family tree on the right shows the inherited relationship between parents and the child, and samples colored purple undergo BFB events in the local region. The SV labeled with a purple square and circle is the FBI inherited from the father sample SRR3949415 and mother sample SRR3949416 to the proband sample SRR3949329. (c) CIRCOS diagram of the complex BFB. The outermost track shows the local genome regions involved with the BFB event. The second outermost track illustrates the input region CNs, and the third track indicates the resolved CNs by Ambigram. Besides, the third track shows the resolved BFB paths, in which the circle and triangle points refer to the 5' end and 3' end, respectively. The innermost part represents all the SVs involved with the BFB event. (d) Ambigram resolved the BFB path. This complex BFB event consists of two stages. In the first stage, the local region on chr21 undergoes seven BFB cycles. The first two cycles occur when chromosomal duplication happens, and segment H21_10 and reverse segment $\overline{H21\_1}$ are fused with reverse complements, respectively. Then another breakage occurs at reverse segment $\overline{H21\_3}$, and a sister chromatid is replicated, followed by the fusion between reverse segment $\overline{H21\_3}$ and segment H21_2. Following that, the fourth BFB cycle fuses segment H21_8 and its reverse complement through a virtual FBI. Moreover, the fifth BFB cycle occurs when another breakage happens on reverse segment $\overline{H21\_5}$, fused with segment H21_4 on the sister chromatid. Then the sixth BFB cycle starts when the breakage occurs at segment H21_4. A sister chromatid is reproduced, and segment H21_4 is fused with reverse segment $\overline{H21\_5}$ on the chromatid duplication. Finally, another breakage occurs at segment H21_2, and a sister chromatid is replicated. The fusion between segment H21_2 and reverse segment $\overline{H21\_3}$ indicates the end of the first stage. In the second stage, a translocation connects segments H19_1 and H21_8, and then another SV happens between segments H21_8 and H21_10, deleting segment H21_9 on the BFB path.

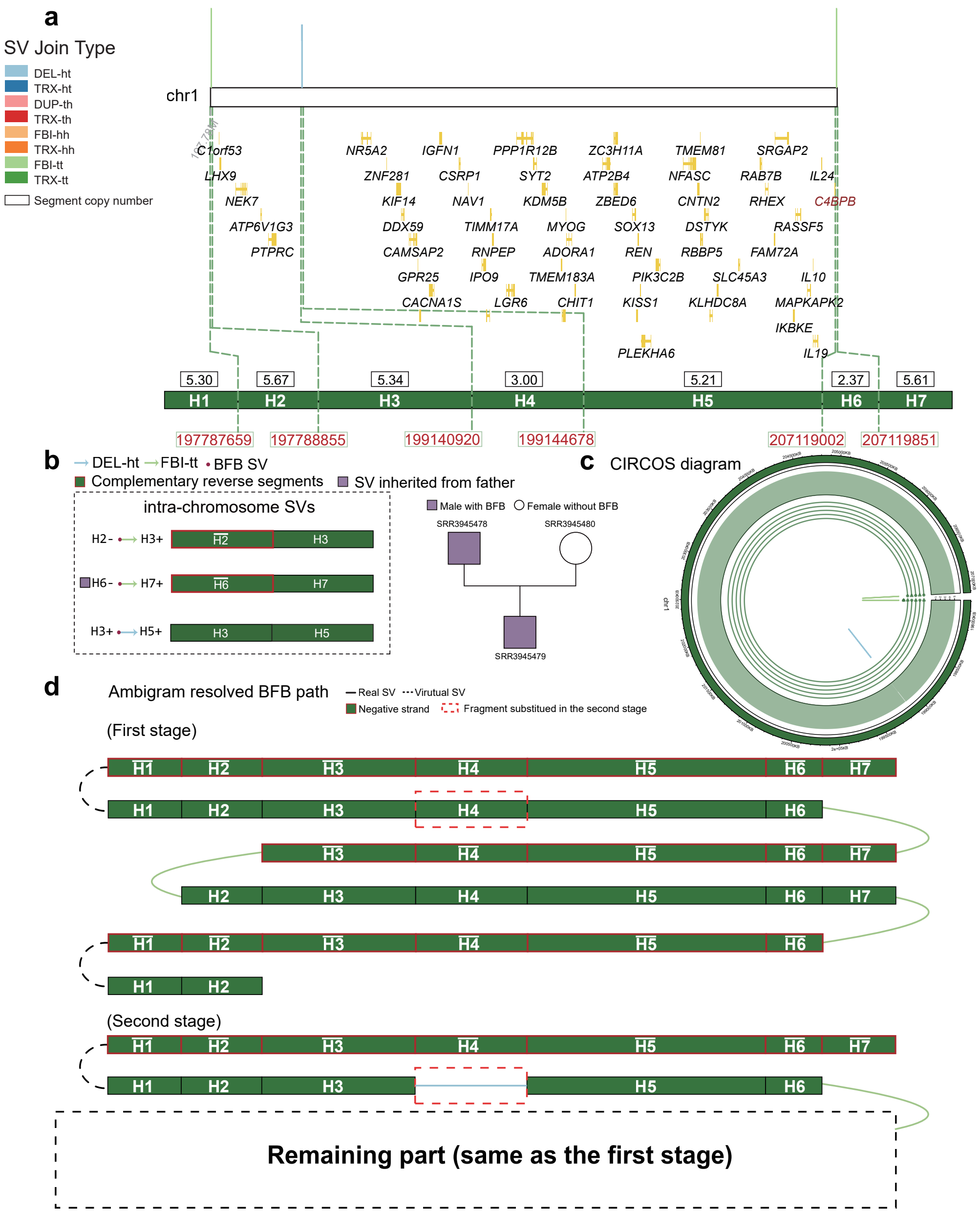

Supplementary Figure 39: Recurrent complex BFB involving chr1 of CHD sample SRR3945479. (a) The SV breakpoints split the local genome region into seven segments. The vertical lines above show the positions of SVs on chr1, and the middle layer shows gene annotation. The black box shows the average CN of segments. (b) List of SVs and the segments connected by them. The head-to-tail (ht) deletion and tail-to-tail (tt) FBIs are colored light blue and light green, respectively. The reverse complementary segments have a red border. The family tree on the right shows the inherited relationship between parents and the child, and samples colored purple undergo BFB events in the local region. In contrast, the sample without color does not have signs of a BFB event. The SV labeled with a purple square is the FBI inherited from the father sample SRR3945478 to the proband sample SRR3945479. (c) CIRCOS diagram of the complex BFB. The outermost track shows the local genome regions involved with the BFB event. The second outermost track illustrates the input region CNs, and the third track indicates the resolved CNs by Ambigram. Besides, the third track shows the resolved BFB paths, in which the circle and triangle points refer to the 5' end and 3' end, respectively. The innermost part represents all the SVs involved with the BFB event. (d) Ambigram resolved the BFB path. This complex BFB event consists of two stages. In the first stage, the local region on chr1 undergoes three BFB cycles. The first BFB cycle fuses segments $\overline{H1}$ and H1, and the second cycle starts when the breakage occurs at segment H6. A sister chromatid is reproduced, followed by the fusion between segment H6 and reverse segment $\overline{H7}$ on the chromatid duplication. Then another breakage occurs at reverse segment $\overline{H3}$, and a sister chromatid is replicated. The final BFB cycle fuses reverse segment $\overline{H3}$ and segment H2, and another breakage on segment H2 indicates the end of the first stage. In the second stage, another SV happens between segment H3 and segment H5, deleting segment H4 on the BFB path.

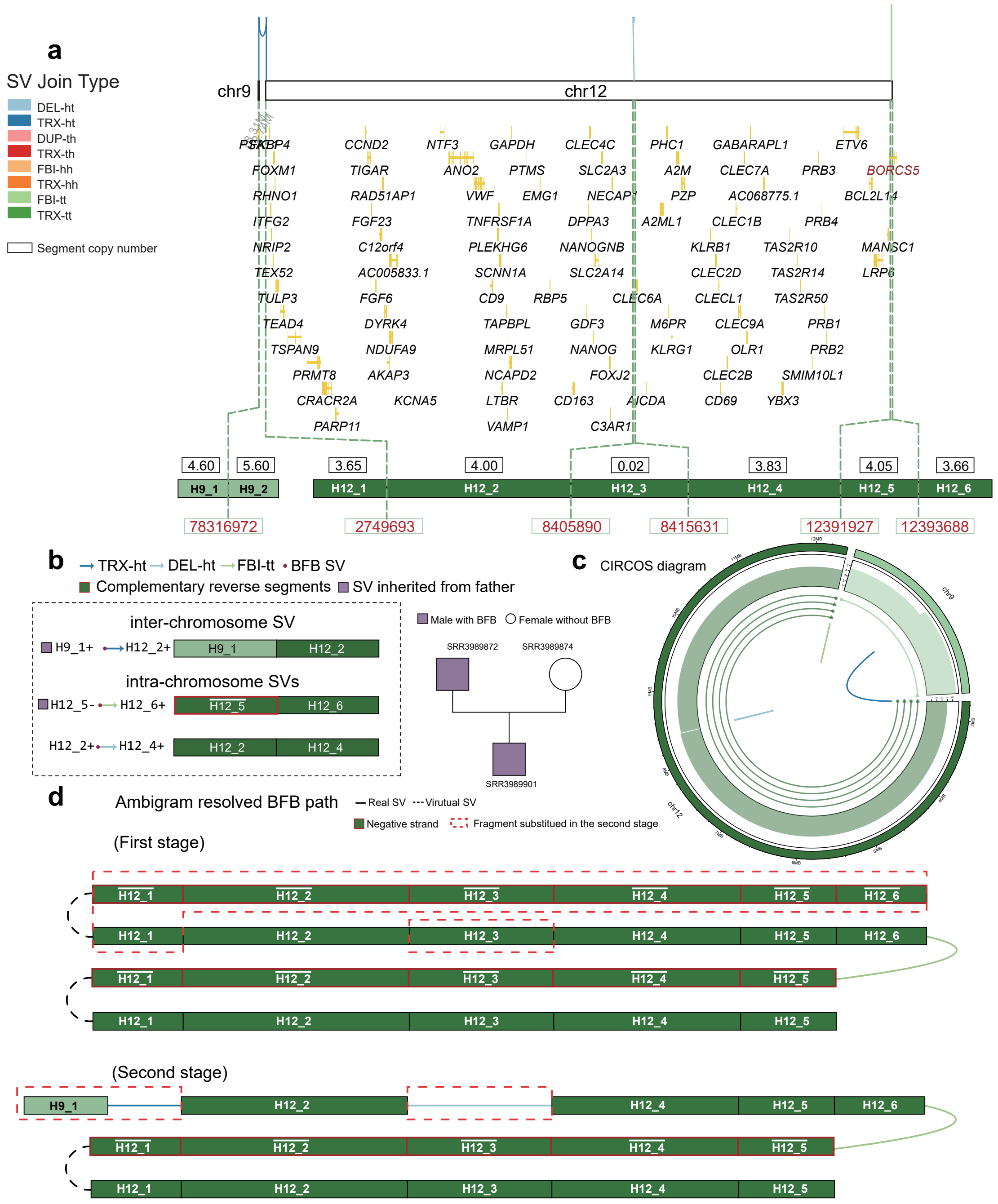

Supplementary Figure 40: Recurrent complex BFB involving chr12 and chr9 of CHD sample SRR3989901. (a) The SV breakpoints split the local genome regions of chr12 and chr9 into six and two segments, respectively. The vertical lines above show the positions of SVs, and the middle layer shows gene annotation. The black box shows the average CN of segments. (b) List of SVs and the segments connected by them. The head-to-tail (ht) translocation, head-to-tail (ht) deletion, and tail-to-tail (tt) FBI are colored blue, light blue, and light orange, respectively. The reverse complementary segments have a red border. The family tree on the right shows the inherited relationship between parents and the child, and samples colored purple undergo BFB events in the local region. In contrast, the sample without color does not have signs of a BFB event. The SV labeled with a purple square is the FBI inherited from the father sample SRR3989872 to the proband sample SRR3989901. (c) CIRCOS diagram of the complex BFB. The outermost track shows the local genome regions involved with the BFB event. The second outermost track illustrates the input region CNs, and the third track indicates the resolved CNs by Ambigram. Besides, the third track shows the resolved BFB paths, in which the circle and triangle points refer to the 5' end and 3' end, respectively. The innermost part represents all the SVs involved with the BFB event. (d) Ambigram resolved the BFB path. This complex BFB event consists of two stages. In the first stage, the local region on chr12 undergoes two BFB cycles. The first cycle occurs when reverse segment $\overline{H12\_1}$ is fused with segment H12_1 on the sister chromatid. Then another breakage occurs at segment H12_6, and a sister chromatid is replicated. The final BFB cycle fuses segments H12_6 and $\overline{H12\_5}$, and the final breakage on segment H12_5 indicates the end of the first stage. In the second stage, a translocation happens between segment H9_1 and segment H12_2, and another SV occurs between segment H12_2 and segment H12_4, deleting segment H12_3 on the BFB path.

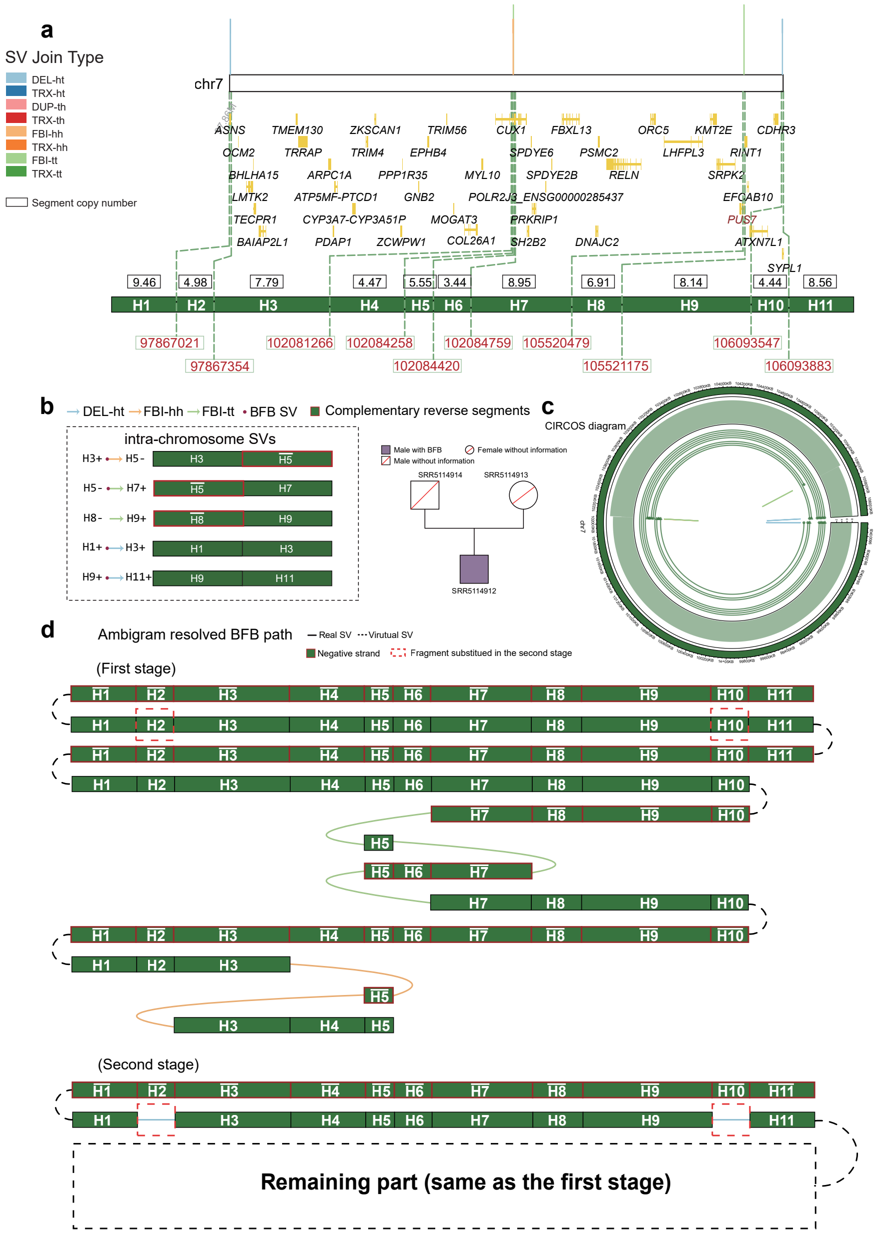

Supplementary Figure 41: Recurrent complex BFB involving chr7 of CHD sample SRR5114912. (a) The SV breakpoints split the local genome regions of chr7 into eleven segments. The vertical lines above show the positions of SVs, and the middle layer shows gene annotation. The black box shows the average CN of segments. (b) List of SVs and the segments connected by them. The head-to-tail (ht) deletion, head-to-head (hh) FBI, and tail-to-tail (tt) FBIs are colored light blue, light yellow, and light orange, respectively. The reverse complementary segments have a red border. The family tree on the right shows the inherited relationship between parents and the child, and the sample colored purple undergo BFB events in the local region. Still, there is not any information about its parents in the dataset. (c) CIRCOS diagram of the complex BFB. The outermost track shows the local genome regions involved with the BFB event. The second outermost track illustrates the input region CNs, and the third track indicates the resolved CNs by Ambigram. Besides, the third track shows the resolved BFB paths, in which the circle and triangle points refer to the 5' end and 3' end, respectively. The innermost part represents all the SVs involved with the BFB event. (d) Ambigram resolved the BFB path. This complex BFB event consists of two stages. In the first stage, the local region on chr7 undergoes seven BFB cycles. The first two cycles occur when chromosomal duplication happens, and segments $\overline{H1}$ and H11 are fused with their reverse complements, respectively. Besides, the third BFB cycle fuses segments H10 and $\overline{H10}$ through a virtual FBI. Then breakage occurs at reverse segment $\overline{H7}$, and a sister chromatid is replicated, followed by the fusion between segments $\overline{H7}$ and H5. Moreover, the fifth BFB cycle occurs when segment H5 has fused with reverse segment $\overline{H7}$ on the sister chromatid. Then the sixth BFB cycle starts when the breakage occurs at segment H3. A sister chromatid is reproduced, and segment H3 is fused with reverse segment $\overline{H5}$ on the chromatid duplication. Finally, another similar BFB cycle fuses segments $\overline{H5}$ and H3, and the breakage on segment H5 indicates the end of the first stage. In the second stage, an SV connects segments H1 and H3, while another SV spans from segment H9 to H11, deleting segments H2 and H10 on the BFB path.

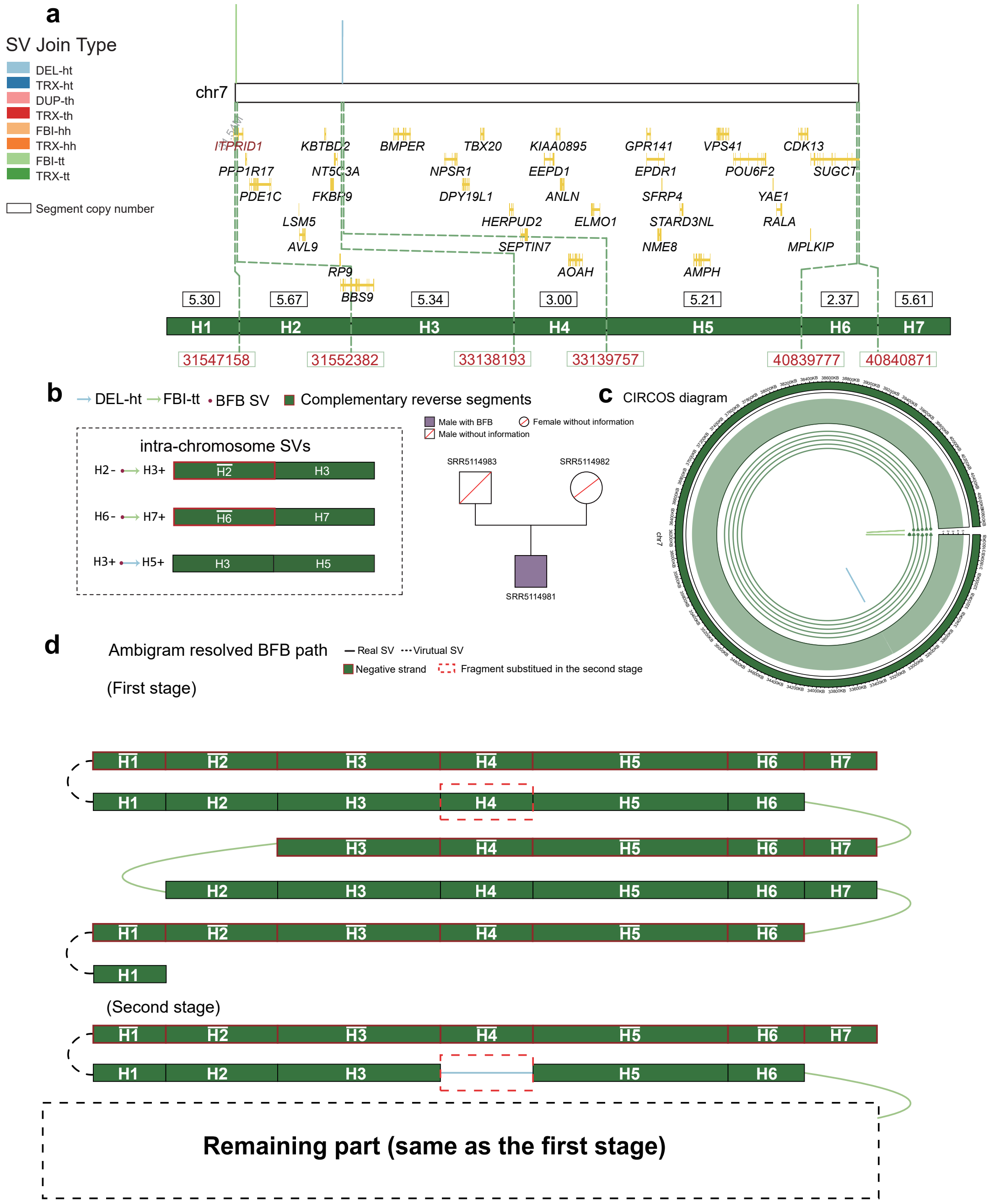

Supplementary Figure 42: Recurrent complex BFB involving chr7 of CHD sample SRR5114981. (a) The SV breakpoints split the local genome region into seven segments. The vertical lines above show the positions of SVs on chr7, and the middle layer shows gene annotation. The black box shows the average CN of segments. (b) List of SVs and the segments connected by them. The head-to-tail (ht) deletion and tail-to-tail (tt) FBIs are colored light blue and light green, respectively. The reverse complementary segments have a red border. The family tree on the right shows the inherited relationship between parents and the child, and the sample colored purple undergoes BFB events in the local region. Still, there is not any information about its parents in the dataset. (c) CIRCOS diagram of the complex BFB. The outermost track shows the local genome regions involved with the BFB event. The second outermost track illustrates the input region CNs, and the third track indicates the resolved CNs by Ambigram. Besides, the third track shows the resolved BFB paths, in which the circle and triangle points refer to the 5' end and 3' end, respectively. The innermost part represents all the SVs involved with the BFB event. (d) Ambigram resolved the BFB path. This complex BFB event consists of two stages. In the first stage, the local region on chr7 undergoes three BFB cycles. The first BFB cycle starts when reverse segment $\overline{H1}$ is fused with segment H1. Then another breakage occurs at segment H6. A sister chromatid is reproduced, followed by the fusion between segment H6 and reverse segment $\overline{H7}$ on the chromatid duplication. Finally, another breakage occurs at reverse segment $\overline{H3}$, and a sister chromatid is replicated. Reverse segment $\overline{H3}$ and segment H2 are fused, and the final breakage on segment H1 indicates the end of the first stage. In the second stage, another SV happens between segment H3 and segment H5, deleting segment H4 on the BFB path.

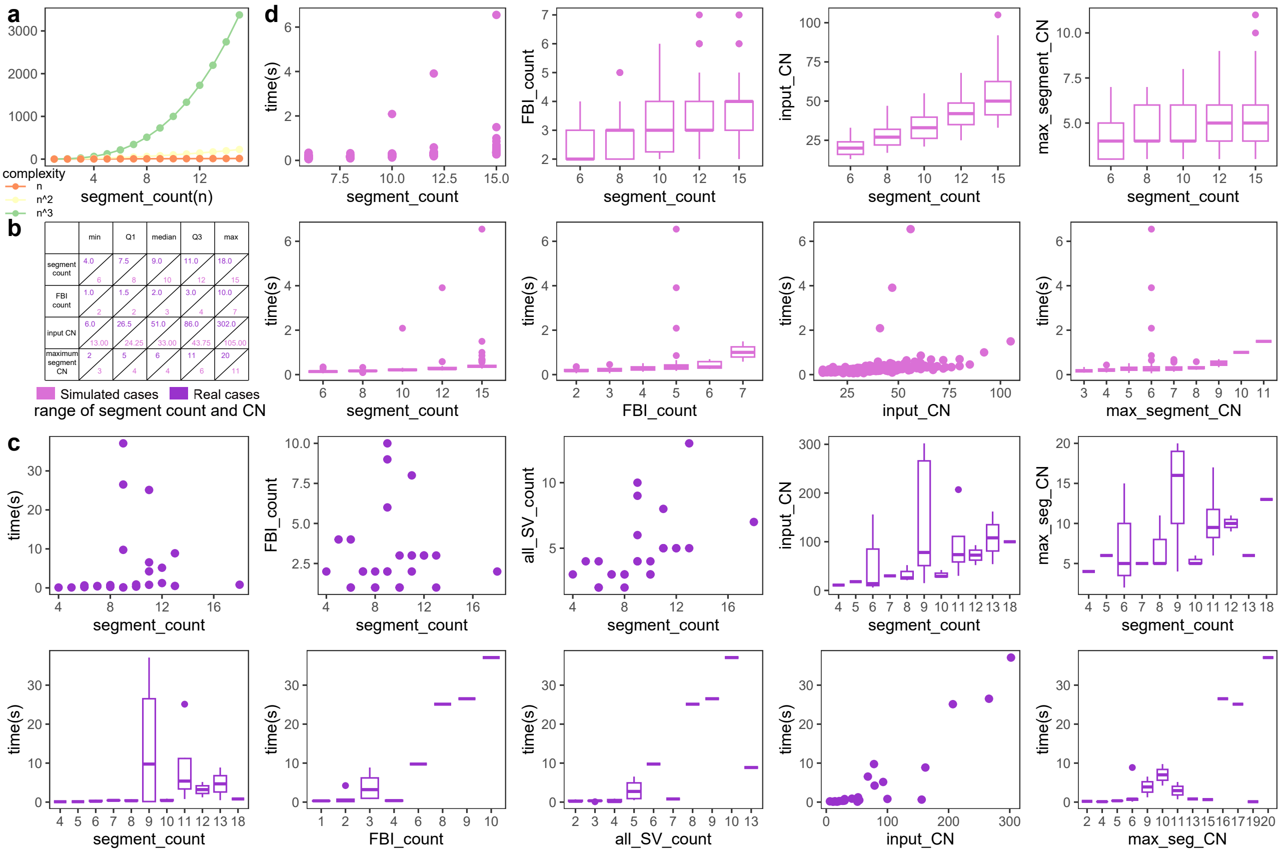

Supplementary Figure 43: Running time evaluation.

(a) The scatterplot of time complexity $O(n^3)$. (b) The quantile of segment counts, FBI count, input CN, and maximum segment CN in real BFB trials and 410 simulated BFB paths. (c) The running time of $n_1 = 27$ real BFB trials. (d) The running time of $n_2 = 410$ simulated BFB paths. We run Ambigram on the platform Ubuntu 20.04 with 12th Gen Intel(R) Core(TM) i7-12700F (20 CPUs) and 32 GB RAM. Box plots indicate the median (middle line), 25th, 75th percentile (box), and 5th and 95th percentile (whiskers) as well as outliers (single points).

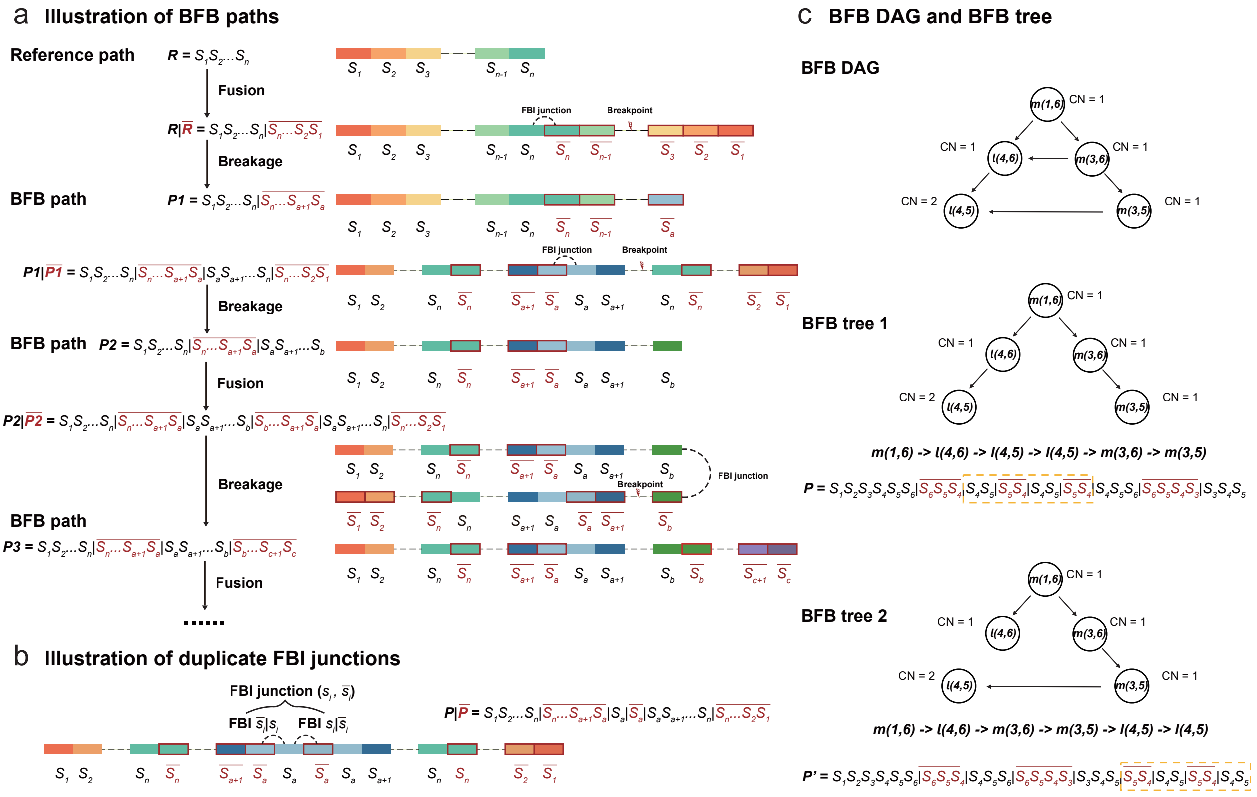

Supplementary Figure 44: Illustration of BFB paths.
(a) Starting with a reference path, each BFB path is obtained by a sequence of BFB cycles that consist of fusion and breakage. In each BFB cycle, a local genomic map is fused with its sister chromatid by an FBI junction, and breakage happens at another FBI breakpoint, contributing to a BFB path. (b) Two consecutive FBIs $s_a|\overline{s_a}$ and $\overline{s_a}|s_a$ happen on the same segment s_a. While the FBI breakpoints are different, we consider them as the same FBI junction $(s, \overline{s_a})$ as they contribute equal CN increase to segment s_a. (c) A BFB path can be equivalently represented by a BFB tree. A child mono-chain is the right child vertex of its parent mono-chain, while a loop is the left child vertex of its parent entity. Through the preorder traversal, entities in a BFB tree can compose a BFB path. Given sequencing data, we can construct a BFB DAG by connecting all pairs of parent and child entities. Since sequencing data cannot determine the exact position for each copy of a loop, a loop with multiple copies is collapsed into one vertex. A BFB tree is built upon the parent-child relationship between entities, so a BFB tree is a sub-tree in a BFB DAG. We can extract multiple BFB trees from a BFB DAG to derive several BFB paths.

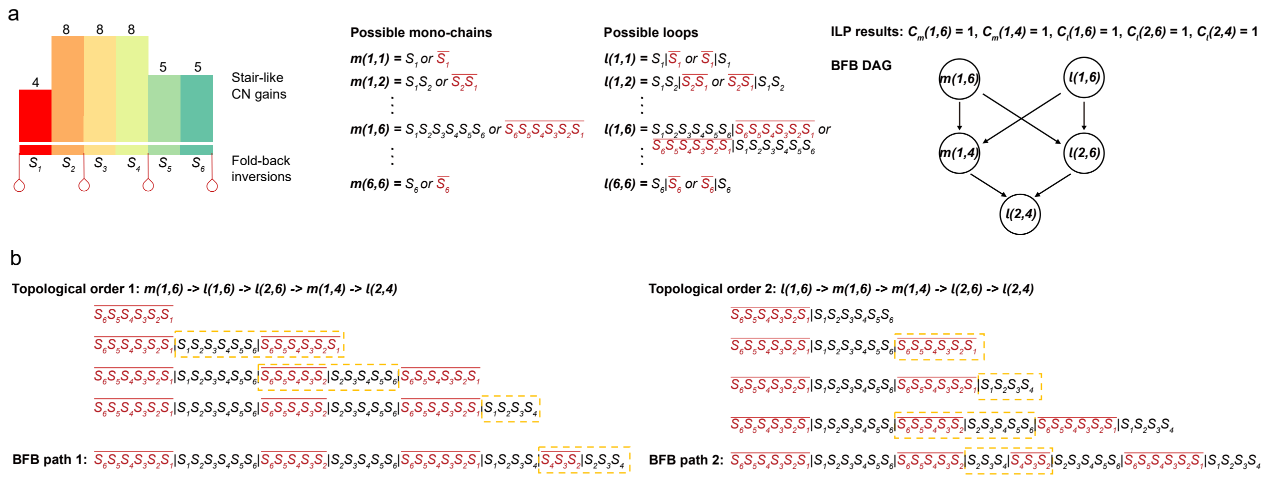

Supplementary Figure 45: One example of perfect BFB.
(a) There are six segments with a stair-like CN pattern and four FBIs. Ambigram first list 21 mono-chains and 21 loops that are possible entities of a BFB path. Then Ambigram uses ILP to adjust errors between observed CNs of segments and estimated CNs of entities and find five entities (two mono-chains and three loops) with CN larger than 0. Finally, Ambigram constructs a DAG consisting of the five entities. (b) Based on the DAG, Ambigram finds two topological orders that derive two different BFB paths. Ambigram keeps a temporary path that the following entities will extend, starting with the first entity. In each step, Ambigram tries to put a new entity into an appropriate position on the temporary path (labeled by dashed yellow rectangles). When all the entities are integrated into one path, the algorithm will stop and output the resultant BFB path.

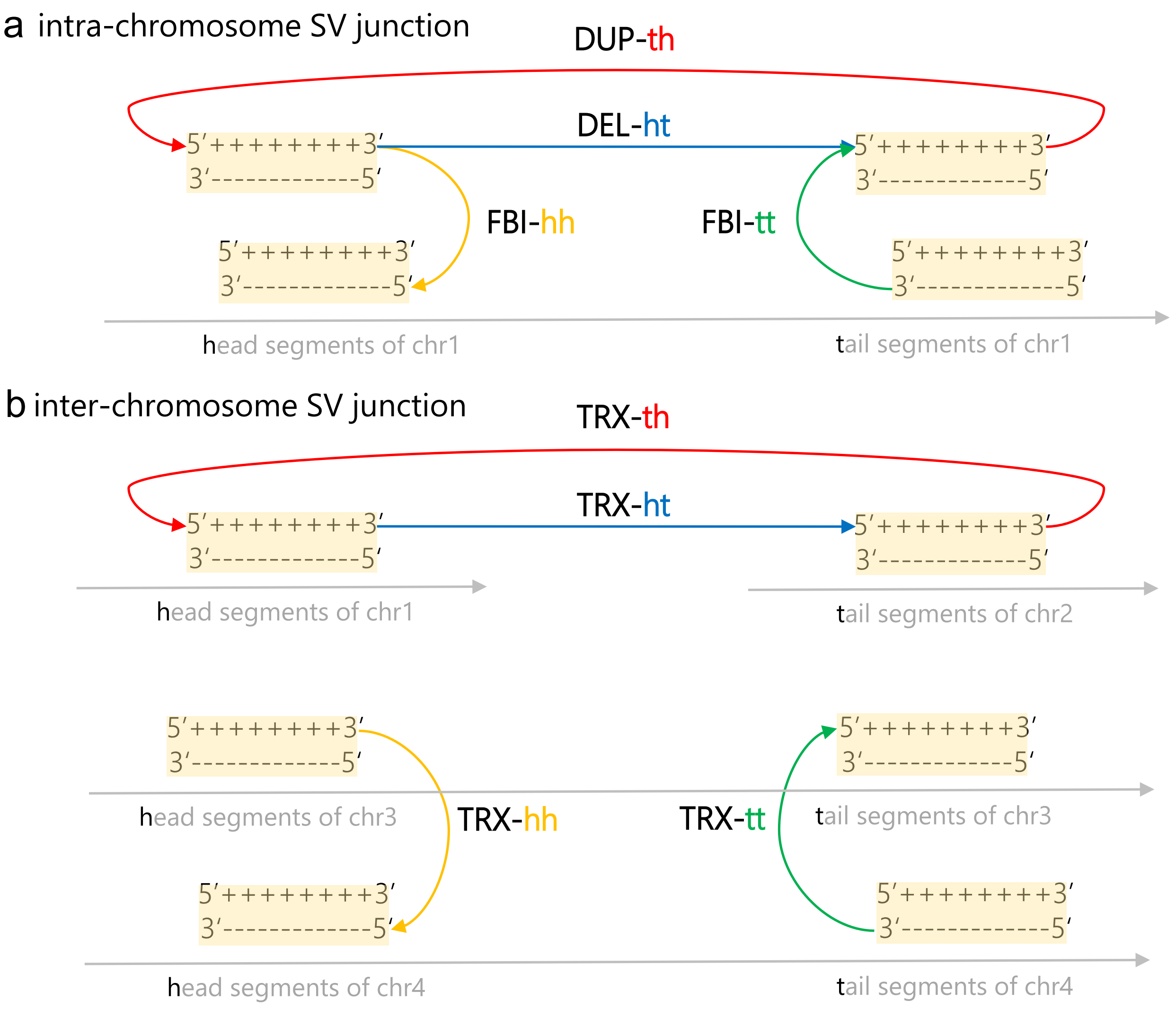

Supplementary Figure 46: The illustration of SV junction type.
(a) The intra-chromosome SV junction can be head-to-tail deletion (DEL-ht), tail-to-head duplication (DUP-th), head-to-head fold-back inversion (FBI-hh), and tail-to-tail fold-back inversion (FBI-tt). (b) The inter-chromosome SV junction can be head-to-tail translocation (TRX-ht), tail-to-head translocation (TRX-th), head-to-head translocation (TRX-hh), and tail-to-tail translocation (TRX-tt).

Supplementary References

[1] Andrew D Rouillard, Gregory W Gundersen, Nicolas F Fernandez, Zichen Wang, Caroline D Monteiro, Michael G McDermott, and Avi Ma'ayan. The harmonizome: a collection of processed datasets gathered to serve and mine knowledge about genes and proteins. *Database*, 2016, 2016.

[2] Viraj Deshpande, Jens Luebeck, Nam-Phuong D Nguyen, Mehrdad Bakhtiari, Kristen M Turner, Richard Schwab, Hannah Carter, Paul S Mischel, and Vineet Bafna. Exploring the landscape of focal amplifications in cancer using ampliconarchitect. *Nature communications*, 10(1):1–14, 2019.

[3] CD Greenman, SL Cooke, J Marshall, MR Stratton, and PJ Campbell. Modeling the evolution space of breakage fusion bridge cycles with a stochastic folding process. *Journal of mathematical biology*, 72(1-2):47–86, 2016.

[4] Shay Zakov, Marcus Kinsella, and Vineet Bafna. An algorithmic approach for breakage-fusion-bridge detection in tumor genomes. *Proceedings of the National Academy of Sciences*, 110(14):5546–5551, 2013.

[5] Wing-Kin Sung, Hancheng Zheng, Shuyu Li, Ronghua Chen, Xiao Liu, Yingrui Li, Nikki P Lee, Wah H Lee, Pramila N Ariyaratne, Chandana Tennakoon, et al. Genome-wide survey of recurrent hbv integration in hepatocellular carcinoma. *Nature genetics*, 44(7):765–769, 2012.

[6] Wenlong Jia, Chang Xu, and Shuai Cheng Li. Resolving complex structures at oncovirus integration loci with conjugate graph. *Briefings in Bioinformatics*, 22(6):bbab359, 2021.

[7] Heng Li. wgsim-read simulator for next generation sequencing. *Github repository*, 2011.

[8] Heng Li. Aligning sequence reads, clone sequences and assembly contigs with bwa-mem. *arXiv preprint arXiv:1303.3997*, 2013.

[9] Jeremiah A Wala, Pratiti Bandopadhayay, Noah F Greenwald, Ryan O'Rourke, Ted Sharpe, Chip Stewart, Steve Schumacher, Yilong Li, Joachim Weischenfeldt, Xiaotong Yao, et al. Svaba: genome-wide detection of structural variants and indels by local assembly. *Genome research*, 28(4):581–591, 2018.

[10] Yukiteru Ono, Kiyoshi Asai, and Michiaki Hamada. Pbsim: Pacbio reads simulator—toward accurate genome assembly. *Bioinformatics*, 29(1):119–121, 2013.

[11] Fritz J Sedlazeck, Philipp Rescheneder, Moritz Smolka, Han Fang, Maria Nattestad, Arndt Von Haeseler, and Michael C Schatz. Accurate detection of complex structural variations using single-molecule sequencing. *Nature methods*, 15(6):461–468, 2018.

[12] Ruibang Luo, Fritz J Sedlazeck, Charlotte A Darby, Stephen M Kelly, and Michael C Schatz. Lrsim: a linked-reads simulator generating insights for better genome partitioning. *Computational and structural biotechnology journal*, 15:478–484, 2017.

[13] Patrick Marks, Sarah Garcia, Alvaro Martinez Barrio, Kamila Belhocine, Jorge Bernate, Rajiv Bharadwaj, Keith Bjornson, Claudia Catalanotti, Josh Delaney, Adrian Fehr, et al. Resolving the full spectrum of human genome variation using linked-reads. *Genome research*, 29(4):635–645, 2019.

[14] Xiaoyu Chen, Ole Schulz-Trieglaff, Richard Shaw, Bret Barnes, Felix Schlesinger, Morten Källberg, Anthony J Cox, Semyon Kruglyak, and Christopher T Saunders. Manta: rapid detection of structural variants and indels for germline and cancer sequencing applications. *Bioinformatics*, 32(8):1220–1222, 2016.

[15] Markus Mayrhofer, Sebastian DiLorenzo, and Anders Isaksson. Patchwork: allele-specific copy number analysis of whole-genome sequenced tumor tissue. *Genome biology*, 14(3):1–10, 2013.

[16] Guillaume Marçais, Arthur L Delcher, Adam M Phillippy, Rachel Coston, Steven L Salzberg, and Aleksey Zimin. Mummer4: A fast and versatile genome alignment system. *PLoS computational biology*, 14(1):e1005944, 2018.

[17] Shay Zakov and Vineet Bafna. Reconstructing breakage fusion bridge architectures using noisy copy numbers. *Journal of Computational Biology*, 22(6):577–594, 2015.

[18] Wenlong Jia, Hechen Li, Shiying Li, Lingxi Chen, and Shuai Cheng Li. Oviz-bio: a web-based platform for interactive cancer genomics data visualization. *Nucleic Acids Research*, 2020.
