# Supplementary material for: Deciphering complex breakage-fusion-bridge genome rearrangements with Ambigram
Source: Nat Commun. 2023 Sep 8;14:5528. doi: 10.1038/s41467-023-41259-w (PMC10491683; doi:10.1038/s41467-023-41259-w)
Supplement: Supplementary file 2 — Reporting Summary [file 41467_2023_41259_MOESM2_ESM.pdf]

nature portfolio

Corresponding author(s): Shuai Cheng Li

Last updated by author(s): Aug 4, 2023

Reporting Summary

Nature Portfolio wishes to improve the reproducibility of the work that we publish. This form provides structure for consistency and transparency in reporting. For further information on Nature Portfolio policies, see our Editorial Policies and the Editorial Policy Checklist.

Statistics

For all statistical analyses, confirm that the following items are present in the figure legend, table legend, main text, or Methods section.

n/a	Confirmed	
☐	☒	The exact sample size (n) for each experimental group/condition, given as a discrete number and unit of measurement
☐	☒	A statement on whether measurements were taken from distinct samples or whether the same sample was measured repeatedly
☐	☒	The statistical test(s) used AND whether they are one- or two-sided<br>*Only common tests should be described solely by name; describe more complex techniques in the Methods section.*
☒	☐	A description of all covariates tested
☐	☒	A description of any assumptions or corrections, such as tests of normality and adjustment for multiple comparisons
☐	☒	A full description of the statistical parameters including central tendency (e.g. means) or other basic estimates (e.g. regression coefficient) AND variation (e.g. standard deviation) or associated estimates of uncertainty (e.g. confidence intervals)
☐	☒	For null hypothesis testing, the test statistic (e.g. F, t, r) with confidence intervals, effect sizes, degrees of freedom and P value noted<br>*Give P values as exact values whenever suitable.*
☒	☐	For Bayesian analysis, information on the choice of priors and Markov chain Monte Carlo settings
☒	☐	For hierarchical and complex designs, identification of the appropriate level for tests and full reporting of outcomes
☒	☐	Estimates of effect sizes (e.g. Cohen's d, Pearson's r), indicating how they were calculated

Our web collection on statistics for biologists contains articles on many of the points above.

Software and code

Policy information about availability of computer code

Data collection	All data were collected by downloading the raw or processed data directly from public platforms or their papers.
Data analysis	The preprocessing tools are summarized in Supplementary Table S3. For data analysis, we used wgsim v1.0, PBSIM v1.0.3, LRSim v1.0, BWA-MEN v0.7.17, NGMLR v0.2.7, Long Ranger v2.2.2, MUMmer v4.0.0, SvABA v1.1.0, Sniffles v1.0.12, Manta v1.6.0, Patchwork v1.1.2, and Ubuntu 20.04. The code of Ambigram is available at https://github.com/deepomicslab/Ambigram.

For manuscripts utilizing custom algorithms or software that are central to the research but not yet described in published literature, software must be made available to editors and reviewers. We strongly encourage code deposition in a community repository (e.g. GitHub). See the Nature Portfolio guidelines for submitting code & software for further information.

Data

Policy information about availability of data

All manuscripts must include a data availability statement. This statement should provide the following information, where applicable:

- Accession codes, unique identifiers, or web links for publicly available datasets
- A description of any restrictions on data availability
- For clinical datasets or third party data, please ensure that the statement adheres to our policy

The COLO829 PE, 10x, PB, and ONT data are downloaded from PRJEB27698 https://www.ebi.ac.uk/ena/browser/view/PRJEB27698. The HCC827 PE and OM data

are retrieved from Luebeck et al. (2020). The PD4875 data is obtained from Greenman et al. (2016). The PD3641 data are obtained from Zakov et al. (2013). COLO829 single-cell data are downloaded from GSE151409 https://0-www-ncbi-nlm-nih-gov.brum.beds.ac.uk/geo/query/acc.cgi?acc=GSE151409. mkn45 single-cell data are downloaded from PRJNA498809 https://www.ncbi.nlm.nih.gov/bioproject/?term=PRJNA498809. The 101T, 260T, and 261T HCC data are obtained from Jia et al. (2021). The S0007T1 HCC data is obtained from the China National GeneBank DataBase (CNGBdb) with accession number CNP0003155 https://db.cngb.org/search/project/CNP0003155/. HELA data and SIHA data are downloaded from SRP048769 https://www.ncbi.nlm.nih.gov/Traces/study/?acc=SRP048769&o=acc_s\%3Aa; CRR046045 data is downloaded from NGDC with accession code CRX040585 https://ngdc.cncb.ac.cn/gsa/browse/CRA001401/CRX040585. The 1000GP data are obtained from ftp://ftp.sra.ebi.ac.uk/vol1/run/}~\cite{byrska2021high. The CHD data are downloaded from dbGaP with accession codes phs001194.v3.p2 https://www.ncbi.nlm.nih.gov/projects/gap/cgi-bin/study.cgi?study_id=phs001194.v3.p2. The processed data in this work are freely available at https://github.com/deepomicslab/Ambigram_paper or https://zenodo.org/badge/latestdoi/428970777}~\cite{li_chaohui_2023_8202100. GRCh37 and GRCh38 reference genomes are downloaded from https://www.ncbi.nlm.nih.gov/datasets/genome/GCF_000001405.26/. The complete T2T genome is downloaded from https://www.ncbi.nlm.nih.gov/datasets/genome/GCA_009914755.3/.

Research involving human participants, their data, or biological material

Policy information about studies with human participants or human data. See also policy information about sex, gender (identity/presentation), and sexual orientation and race, ethnicity and racism.

Reporting on sex and gender	No new data was generated for this study. All results are based on published data which have been studied in their original publications. Therefore, no reporting on sex and gender in this study.
Reporting on race, ethnicity, or other socially relevant groupings	No new data was generated for this study. All results are based on published data which have been studied in their original publications. Therefore, no reporting on population characteristics in this study.
Population characteristics	No new data was generated for this study. All results are based on published data which have been studied in their original publications. Therefore, no reporting on population characteristics in this study.
Recruitment	No new data was generated for this study. All results are based on published data which have been studied in their original publications. Therefore, no reporting on population characteristics in this study.
Ethics oversight	No new data was generated for this study. All results are based on published data which have been studied in their original publications. Therefore, no reporting on population characteristics in this study.

Note that full information on the approval of the study protocol must also be provided in the manuscript.

Field-specific reporting

Please select the one below that is the best fit for your research. If you are not sure, read the appropriate sections before making your selection.

☒ Life sciences ☐ Behavioural & social sciences ☐ Ecological, evolutionary & environmental sciences

For a reference copy of the document with all sections, see nature.com/documents/nr-reporting-summary-flat.pdf

Life sciences study design

All studies must disclose on these points even when the disclosure is negative.

Sample size	A collection of 14 datasets from previously pulished studies were used. No changes to the size of datasets (numbers of cells or numbers of genes) were made. For those datasets, we selected 27 real BFB events for analysis. We did this because we wanted to focus on dataset sizes that are representative of typical CSV datasets. We simulated 6 instances to test the performance of Ambigram on deciphering various complex BFB events. We also simulated 410 instances to measure the efficiency of Ambigram under varying conditions.
Data exclusions	No data was excluded. Filtering and quality control of samples is described in the Methods.
Replication	All the computational experiments were replicated two times on Ububtu 20.04 systems. All attempts at replication were successful.
Randomization	There is no randomization involved in this study. Our algorithm detect the complex BFB events for each sample without random allocation inside the algorithm.
Blinding	No new data was generated for this study. All results are based on published data which have been studied in their original publications. Therefore, blinding from investigator is not possible when we reanalyzed the collected data. The computational algorithm do not include the training step, hence all samples were used to generate the unbiased results without group allocation.

Reporting for specific materials, systems and methods

We require information from authors about some types of materials, experimental systems and methods used in many studies. Here, indicate whether each material, system or method listed is relevant to your study. If you are not sure if a list item applies to your research, read the appropriate section before selecting a response.

Materials & experimental systems

n/a	Involved in the study
☒	☐ Antibodies
☒	☐ Eukaryotic cell lines
☒	☐ Palaeontology and archaeology
☒	☐ Animals and other organisms
☒	☐ Clinical data
☒	☐ Dual use research of concern
☒	☐ Plants

Methods

n/a	Involved in the study
☒	☐ ChIP-seq
☒	☐ Flow cytometry
☒	☐ MRI-based neuroimaging
